# Selectfluor and TBAX (Cl, Br) Mediated Oxidative Chlorination and Bromination of Olefins

**DOI:** 10.1021/acs.orglett.2c02627

**Published:** 2022-09-21

**Authors:** Ziya Dağalan, Ramazan Koçak, Arif Daştan, Bilal Nişancı

**Affiliations:** Department of Chemistry, Faculty of Sciences, Ataturk University, Erzurum 25240, Turkey

## Abstract

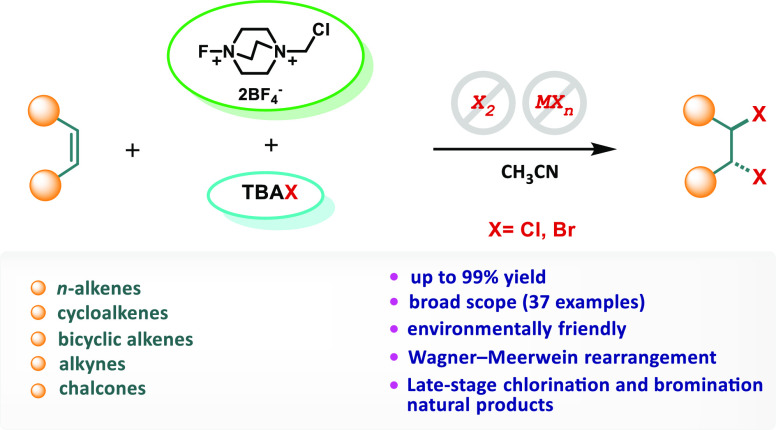

Herein, we report the first metal-free and molecular halogen reagent-free dihomohalogenation methodology by using Selectfluor as an oxidant and tetrabutylammonium bromide/chloride salts as a halogen source. This effective strategy provides various fluorine-free halogenated products easily in quantitative yields from alkenes, alkynes, and natural products.

Halogenation reactions have gained great importance since their discovery, and halides are important in organic synthesis as they are used in the synthesis of various valuable compounds.^1,2^ Among the halides, chlorides and bromides attract more attention than iodides and fluorides owing to their great commercial importance.^3^ On account of chlorine gas or bromine being extremely toxic, researchers are seeking to develop new halogenation strategies.^4,5^ In addition, conducting chemistry without using metals is also crucial in terms of medicinal chemistry^6^ and late-stage functionalization (LSF) of natural products and pharmaceuticals.^7^

Selectfluor is not only a well-known, safe, easily soluble, easy-to-handle, stable solid, and reactive electrophilic fluorination reagent^8,9^ but is also a robust and efficient “fluorine-free” green oxidant in organic synthesis.^10−12^ Selectfluor has been used effectively as an oxidant in transition metal-catalyzed cross-coupling reactions,^13^ selective oxidations,^14^ regioselective ring openings,^15^ Diels–Alder cycloadditions,^16,17^ carbene transfer reactions,^18^ and other organic transformations^19,20^ hitherto. Moreover, oxidative monochlorination of some reactive heterocycles, such as aminopyridines and aminodiazines, was efficiently performed using metal salts with the help of Selectfluor.^21^

Interestingly, tetrabutylammonium bromide and chloride salts have not yet been discovered in oxidative dihomohalogenation reactions until now. To our delight, in this work, we report Selectfluor-mediated metal-free dihomobromination and dihomochlorination methodologies using tetrabuthylammonium bromide/chloride salts under mild reaction conditions with short reaction times.

First, we used benzonorbornadiene **1k** as a test molecule to optimize the halogenation conditions (Table 1). Studies to determine the solvent of the reaction (Table 1, entries 1–4) revealed that acetonitrile was the best choice for bromination (Table 1, entry 4) within 5 min. Next, it should be noted that oxidative bromination does not occur even with stirring for 24 h without Selectfluor (Table 1, entry 5). The amounts of reagents were gradually increased in order to obtain a quantitative yield (Table 1, entries 6 and 7), and the best result was achieved using 1.2 equiv of Selectfluor and 2.4 equiv of tetrabutylammonium bromide salt (Table 1, entry 7). From the oxidative chlorination point of view, an 18% yield was obtained at room temperature in a 1 h reaction (Table 1, entry 8). In an effort to increase the yield, the reaction temperature was set to 100 °C, and 80% yield was obtained as a result of the 1 h reaction (Table 1, entry 9), while a 2 h reaction led to the synthesis of dicholoro benzonorbornadiene quantitatively (Table 1, entry 10). As a result of detailed optimization experiments, the presented oxidative bromination/chlorination protocol was performed when 1.2 equiv of Selectfluor and 2.4 equiv of TBAB in 2 mL of CH_3_CN was used in 5 min at room temperature for bromination (Table 1, entry 7) and 2 h at 100 °C for chlorination (Table 1, entry 10).

**Table 1:**
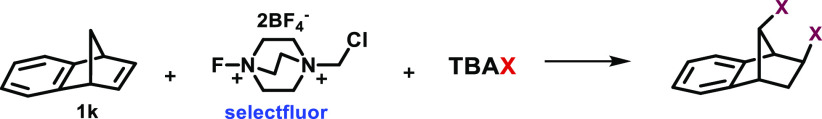

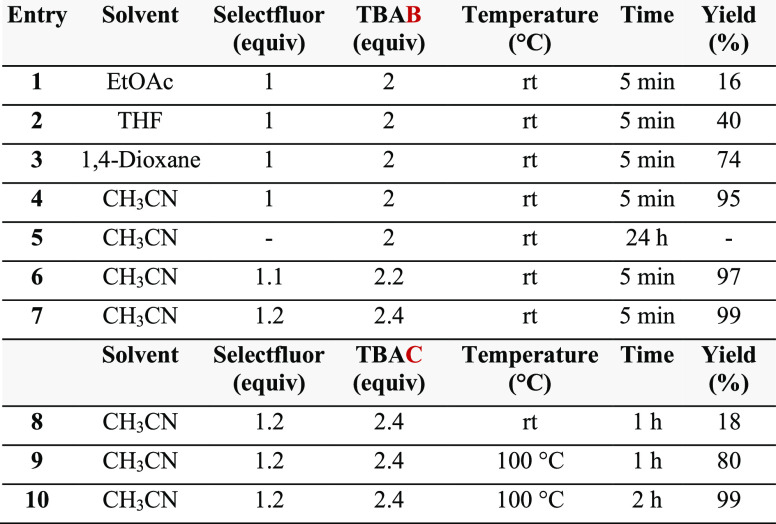
Optimizing the Conditions for the Bromination and Chlorination of Benzonorbornadiene^a^

a Reaction conditions: Benzonorbornadiene (**1k**) (0.5 mmol), Selectfluor (209 mg, 0.6 mmol), and TBAC (333 mg, 1.2 mmol) or TBAB (386 mg, 1.2 mmol), 2 mL of CH_3_CN, 5 min and room temperature for bromination, 2 h and 100 °C for chlorination. Conversions were calculated by ^1^H NMR with 1,3-dinitrobenzene as an internal standard.

After obtaining the optimum oxidative halogenation conditions from bicyclic **1k**, reactions of different unsaturated analogues (26 examples) were examined to extend the scope of the developed methodology (Scheme 1). Under these circumstances, many dihalogenated structures were synthesized in good to excellent yields (75%–99%). Oxidative halogenation of the aliphatic alkene **1a** enabled the synthesis of **2a** and **3a** in yields of 92% and 98%, respectively. The corresponding halogenation products were obtained in high yields similarly from the halogenation of the cyclic alkenes **1b** and **1c**. Styrene **1d** and its derivatives with an activated (**1e**) and a deactivated double bond (**1f**) also worked efficiently under this oxidative system. Oxidative chlorination of polycyclic hydrocarbon **1g** resulted in 85% yield, while the bromination occurred with 96%. In the case of alkynes (**1h**), the corresponding dihalogens were obtained (**2h**, **3h**) selectively. Dibenzosuberenone provided the related dichloro (**2i**) and dibromo (**3i**) compounds quantitatively in 24 h. As expected, the oxidative chlorination of a deactivated double bond is especially difficult. Halogenation of chalcone **1j** enabled the synthesis of the dichloro compound (**2j**) in 75% yield and the brominated one (**3j**) in 93% yield in 24 h. Oxidation of **1l** resulted in the rearrangement products **2l** (93%) and **3l** (97%). Finally, another bicyclic structure, **1m**, was investigated and the chlorination product was obtained in 88% yield and the bromination product in 97% yield. In general, when the reaction times were extended, elimination products were also formed.

**Scheme 1:**
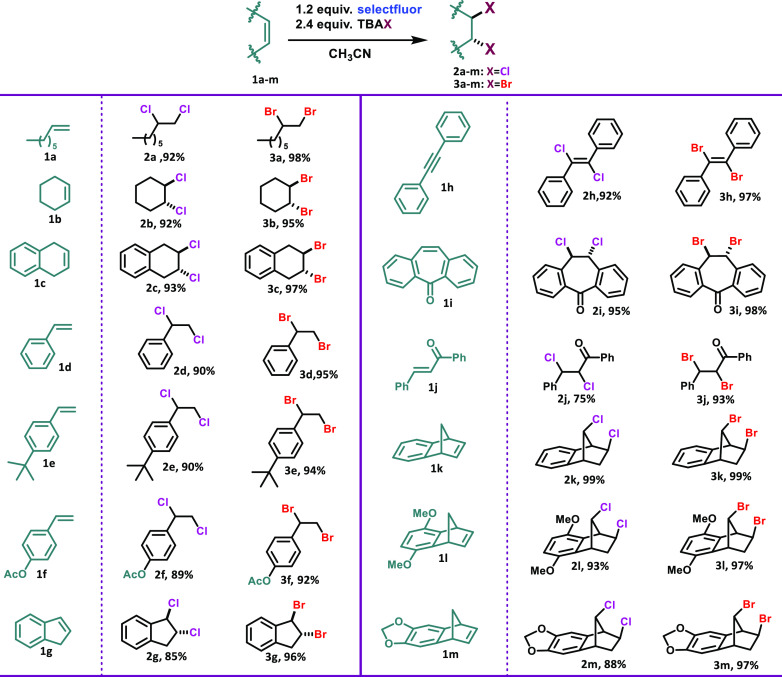
Oxidative Chlorination and Bromination of Olefins with Selectfluor and TBAX (Cl, Br) All reactions were carried out using 0.5 mmol of olefin, 0.6 mmol of Selectfluor, and 1.2 mmol of TBAX (Cl, Br) in 2 mL of CH_3_CN. For chlorination: 100 °C, 2 h (**1i** and **1j**: 24 h). For bromination: rt, 5 min (**1i** and **1j**: 24 h). Conversions were calculated by ^1^H NMR with 1,3-dinitrobenzene as internal standard.

LSF strategies are currently receiving great interest in both the drug discovery and chemical biology fields because they enable the efficient derivatization of natural products.^7,22^ Oxidative bromination and chlorination studies were carried out on 5 different natural products to show that the present method allows derivatization of natural products efficiently in high yields (Scheme 2). When both (+) and (−) enantiomers of camphene were halogenated separately, Wagner–Meerwein rearrangement products dichloro **2n** and **2o** (80%) and dibromo **3n** and **3o** (90%) were obtained. By bromination of carvone enantiomers, dibromo compounds **3p**–**3q** and **3r**–**3s** were obtained in a 1:1 diastereomeric mixture (92%), as expected. However, when the carvone enantiomers were chlorinated, **2p** (60%) and **2q** (62%) were formed by elimination following the chlorination. From the halogenation of cholesterol under general reaction conditions, dibromo **3t** was obtained in 88% yield, but dichloro **2r** could not be isolated (Table 2). The successful transformation of cholesterol to the dibromide **3t** showed that the developed method could be used for a late-stage functionalization reaction. All of the natural products used in the present study were chiral compounds. Because halogenation and Wagner–Meerwein rearrangement do not cause racemization and the initial enantiomeric excess is preserved in the resulting products, no extra analysis was required for the determination of enantiomeric excess.

**Table 2:**
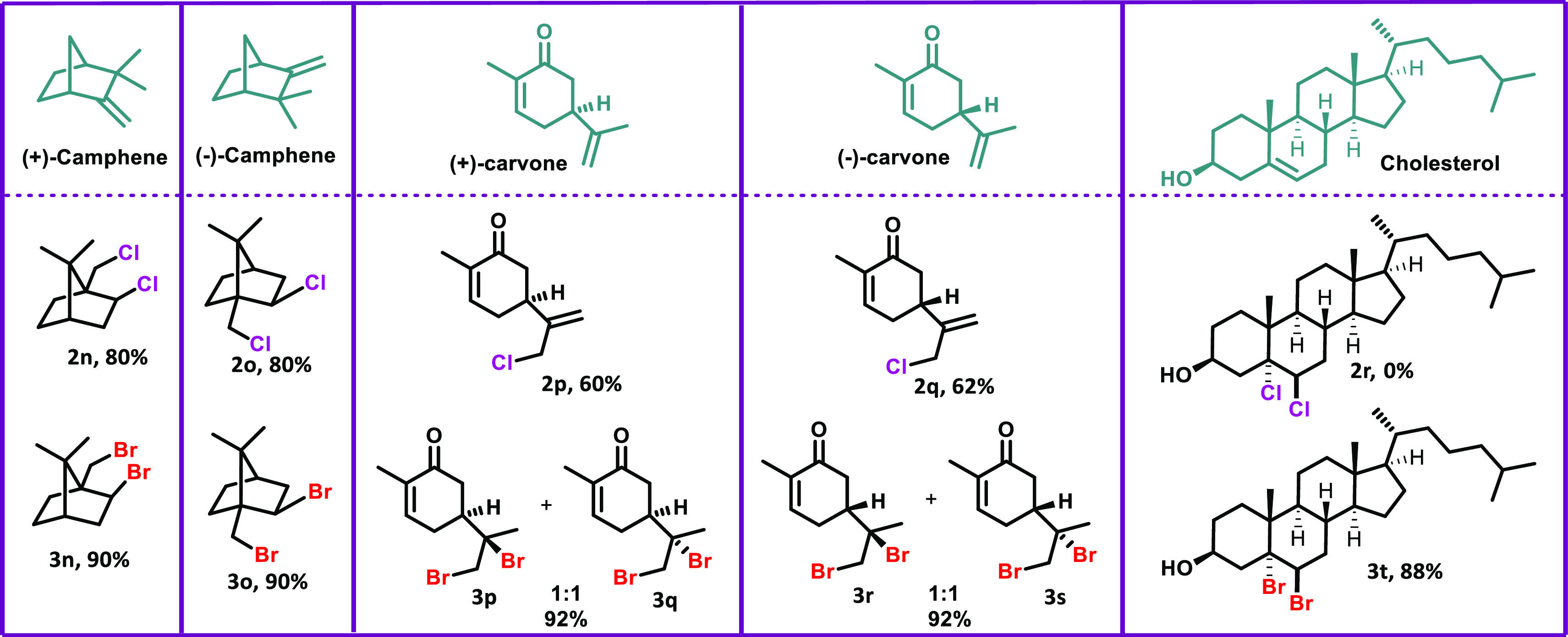
Oxidative Halogenation of Natural Products^a^

a All reactions were carried out using 0.5 mmol of natural product, 0.6 mmol of Selectfluor, and 1.2 mmol of TBAX (Cl, Br) in 2 mL of CH_3_CN. For chlorination: 100 °C, 2 h. For bromination: rt, 5 min. Conversions were calculated by ^1^H NMR with 1,3-dinitrobenzene as internal standard.

**Scheme 2:**
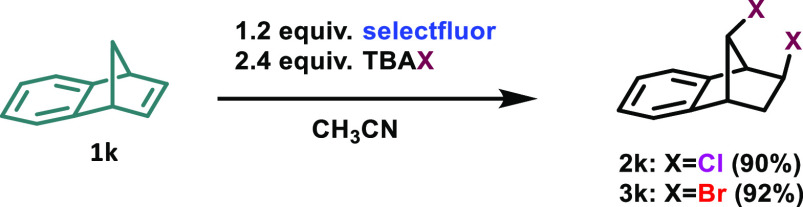
Scale-Up Experiments Reaction conditions: Benzonorbornadiene (1g, 7.03 mmol), Selectfluor (2.94 g, 8.44 mmol), TBAX (Cl, Br) (16.87 mmol), CH_3_CN (20 mL). For chlorination: 100 °C, 2 h. For bromination: rt, 5 min.

Scale-up experiments were performed in order to put forth the practicality of the oxidative halogenation methodology by using benzonorbornadiene (1 g, 7.03 mmol, 14-fold increase) as a test molecule under the optimized reaction conditions. As can be clearly seen from Scheme 2, the isolated yields of **2k** (1.35 g, 90% yield) and **3k** (1.95 g, 92%) are quite satisfactory.

For the oxidative halogenation, we propose the mechanism given in Scheme 3. The Wagner Meerwein rearrangement during the bromination of benzonorbornadiene and its derivatives strongly showed that the reaction proceed via cationic intermediates. In this mechanism, first Selectfluor oxidizes halogen anion X^–^ to halogen cation X^+^. Then the alkene attacks X^+^. Classical halogenation occurs in alkenes, cyclic alkenes, acetylenes, and chalcones by attack of X^–^ on the positive charge center. On the other hand, in bicyclic alkenes, halogenation occurs by attack of X^–^ via Wagner–Meerwein rearrangement.^23^

**Scheme 3:**
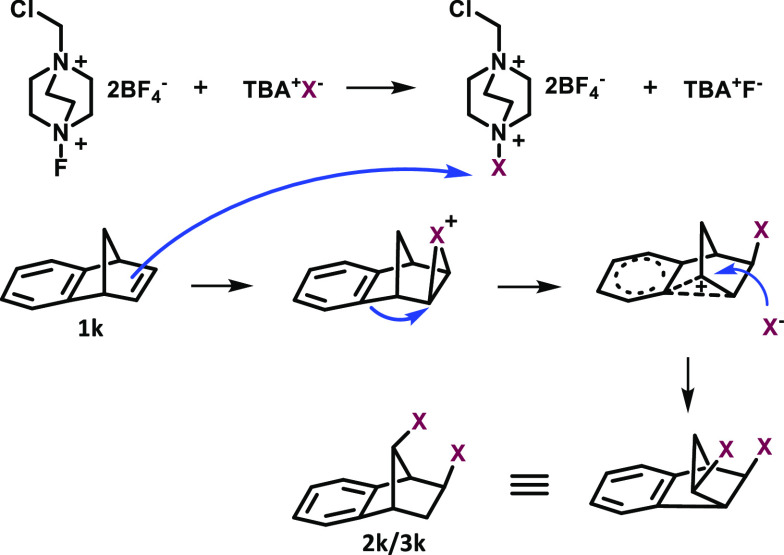
Proposed Mechanism for Halogenation of Bicyclic Alkenes by Wagner–Meerwein Rearrangement

In summary, we succeeded in developing the first metal- and molecular halogen-free dihalogenation of several olefins by using TBAX (Br, Cl) as a halogen source and Selectfluor as an oxidant. Both the olefin functionalization (26 examples) and the LSF experiments (11 examples) of natural products worked perfectly with the yields mostly reaching over 95%. The major advantages of the presented methodology over the existing ones are that (i) there is no need for the molecular chlorine gas and bromine reagent, which are extremely toxic, (ii) it does not require the presence of any metal, (iii) it enables the synthesis of corresponding halogenated products with high yields and selectivity, (iv) it allows derivatization of natural chiral molecules, and (v) it uses a safe solvent in mild conditions. Therefore, we think that this oxidative halogenation protocol will create a new perspective for the dihomohalogenation of olefins and natural products.

## Supporting Information Available

Experimental procedures, NMR, and HR-MS spectra of products (PDF)
