# Supplementary material for: Selectfluor and TBAX (Cl, Br) Mediated Oxidative Chlorination and Bromination of Olefins
Source: Org Lett. 2022 Sep 21;24(45):8261–4. doi: 10.1021/acs.orglett.2c02627 (PMC9680025; doi:10.1021/acs.orglett.2c02627)
Supplement: Supplementary file 1 — ol2c02627_si_001.pdf [file ol2c02627_si_001.pdf]

Supporting Information

Selectfluor and TBAX (Cl, Br) mediated oxidative chlorination and bromination of olefins

Ziya Dağalan, Ramazan Koçak *, Arif Daştan and Bilal Nişancı *

Department of Chemistry, Faculty of Science, Atatürk University,25240 Erzurum, Turkey.

e-mail: ramazan.kocak@atauni.edu.tr ; bnisanci@atauni.edu.tr

Table of Contents

Experimental S2

General procedure..........S2

References..........S10

^{1}H NMR, ^{13}C NMR, and HRMS Spectra..........S12

Experimental

General

Compounds **1c**, **1k**, **1l** and **1m** were synthesized following the procedures reported in the literature.[1-3] All chemicals and solvents, purchased from Sigma-Aldrich, were used without further purification. Reactions that require heating were carried out under oil bath conditions. Reactions were monitored by thin layer chromatography using Merck TLC Silica gel 60 F_{254} and the plates were inspected by 254 nm or 365 nm UV-ligth and/or by acquiring ^{1}H-NMR spectra. Column chromatography was performed over Merck Silica gel 60F (70-230 mesh ASTM). The ^{1}H- and ^{13}C-NMR spectra were recorded on a Varian-400 or a Bruker-400 spectrometer in $CDCl_3$ using tetramethylsilane as the interrnal reference. All spectra were recorded at 25 °C and coupling constants (*J* values) are given in Hz. Chemical shifts are given in parts per million (ppm). Abbreviations used to define the multiplicities are as follows: s = singlet; d = doublet; dd = doublet of doublets; m = multiplet. Mass spectra of unknown compounds (**2i**, **2l-m** and **3l-3m**) were recorded on an Agilent Technologies 6530 Accurate-Mass Q-TOF-LC/MS.

General procedure

For Chlorination

Olefine (**1**) (0,5 mmol), Selectfluor (209 mg, 0,6 mmol) and TBAC (333 mg, 1.2 mmol) were dissolved in 2 mL CH_3CN in an ACE pressure tube. The reaction mixture was stirred at 100 °C for 2 hours (for **1i** and **1j**: 24 h). The mixture was cooled to RT and the solvent was evaporated under reduced pressure. The crude reaction mixture was purified by column chromatography on silica gel (*n*- hexane/EtOAc (9:1)).

For Bromination

Olefine (**1**) (0,5 mmol), Selectfluor (218 mg, 0,6 mmol) and TBAB (386 mg, 1.2 mmol) were dissolved in 2 mL CH_3CN in an ACE pressure tube. The reaction mixture was stirred at room temperature for 5 minutes (for **1i** and **1j**: 24 h). The mixture was cooled to RT and the solvent was evaporated under reduced pressure. The crude reaction mixture was purified by column chromatography on silica gel (*n*-hexane/EtOAc (9:1)).

1,2-Dichlorooctane (2a)[4]

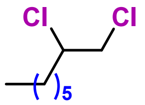

Colorless oil (91 mg, 92%). ^{1}H-NMR (400 M Hz, $CDCl_3$) δ 4.11 – 3.97 (m, 1H), 3.76 (dd, *J* = 11.3, 5.2 Hz, 1H), 3.65 (dd, *J* = 11.3, 7.4 Hz, 1H), 2.07 – 1.92 (m, 1H), 1.76 – 1.64 (m, 1H), 1.37 – 1.16 (m, 8H), 0.89 (t, *J* = 6.7 Hz, 3H). ^{13}C NMR (101 MHz, $CDCl_3$) δ 61.4, 48.4, 35.2, 31.7, 28.8, 25.9, 22.7, 14.2.

***trans*-1,2-Dichlorocyclohexane (2b)**[4]

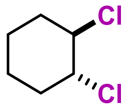

Colorless oil (70 mg, 92%). ^{1}H NMR (400 MHz, $CDCl_3$) δ 4.13 – 3.95 (m, 2H), 2.42 – 2.21 (m, 2H), 1.92 – 1.66 (m, 4H), 1.51 – 1.35 (m, 2H). ^{13}C NMR (101 MHz, $CDCl_3$) δ 63.3, 33.5, 23.2.

***trans*-2,3-Dichloro-1,2,3,4-tetrahydronaphthalene (2c)**[5]

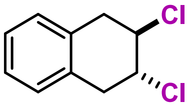

Colorless oil (93 mg, 93%). ^{1}H NMR (400 MHz, $CDCl_3$) δ 7.23 – 7.16 (AA' part of AA'BB' system, 2H), 7.14 – 7.07 (BB' part of AA'BB' system, 2H), 4.50 – 4.44 (m, 2H), 3.67 (dd, A part of AB system, *J* = 18.2, 3.2 Hz, 2H), 3.13 (bd, B part of AB system, *J* = 18.2, 2H). ^{13}C NMR (101 MHz, $CDCl_3$) δ 131.3, 129.0, 126.9, 58.0, 34.8.

(1,2-Dichloroethyl)benzene (2d)[6]

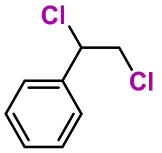

Colorless oil (78 mg, 90%). ^{1}H NMR (400 MHz, $CDCl_3$) δ 7.48 – 7.33 (m, 5H), 5.01 (dd, *J* = 7.9, 6.6 Hz, 1H), 4.01 (dd, *J* = 11.3, 6.6 Hz, 1H), 3.94 (dd, *J* = 11.3, 7.9 Hz, 1H). ^{13}C NMR (101 MHz, $CDCl_3$) δ 138.1, 129.3, 129.0, 127.5, 61.9, 48.5.

1-(*tert*-Butyl)-4-(1,2-dichloroethyl)benzene (2e)[6]

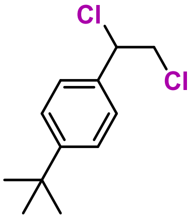

Colorless oil (104 mg, 90%). ^{1}H NMR (400 MHz, $CDCl_3$) δ 7.41 (d, *J* = 8.4 Hz, 2H), 7.34 (d, *J* = 8.4 Hz, 2H), 5.00 (t, *J* = 7.2 Hz, 1H), 4.02 – 3.90 (m, 2H), 1.33 (s, 3H). ^{13}C NMR (101 MHz, $CDCl_3$) δ 152.4, 135.1, 127.2, 125.9, 62.0, 48.6, 34.8, 31.4.

4-(1,2-Dichloroethyl)phenyl acetate 2(f)[7]

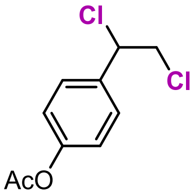

Colorless oil (103 mg, 89%). ^{1}H NMR (400 MHz, $CDCl_3$) δ 7.42 (d, *J* = 8.7 Hz, 2H), 7.12 (d, *J* = 8.7 Hz, 2H), 5.11 – 4.90 (m, 1H), 3.97 (dd, *J* = 11.4, 6.6 Hz, 1H), 3.89 (dd, *J* = 11.4, 7.8 Hz, 1H), 2.29 (s, 3H). ^{13}C NMR (101 MHz, $CDCl_3$) δ 169.2, 151.1, 135.5, 128.6, 122.0, 61.2, 48.4, 21.1.

***trans*-1,2-Dichloro-2,3-dihydro-1H-indene (2g)[6]**

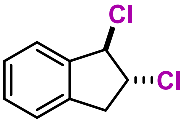

Colorless oil (79 mg, 85%). ^{1}H NMR (400 MHz, $CDCl_3$) δ 7.48 – 7.43 (m, 1H), 7.40 – 7.23 (m, 3H), 5.35 (d, *J* = 2.8 Hz, 1H), 4.66 (dt, *J* = 6.1, 3.2 Hz, 1H), 3.71 (dd, *J* = 16.8, 6.1 Hz, 1H), 3.19 (dd, *J* = 16.8, 3.2 Hz, 1H). ^{13}C NMR (101 MHz, $CDCl_3$) δ 140.0, 129.8, 128.1, 125.6, 125.2, 67.8, 64.6, 40.9 (2C signal overlaps).

(*E*)-1,2-Dichloro-1,2-diphenylethene (2h)[8]

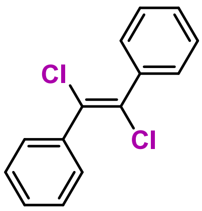

White solid (114 mg, 92%). Mp: 92-94 ˚C. ^{1}H NMR (400 MHz, $CDCl_3$) δ 7.66 – 7.59 (m, 4H), 7.50 – 7.36 (m, 6H). ^{13}C NMR (101 MHz, $CDCl_3$) δ 137.7, 129.3, 129.2, 128.4, 127.9.

***trans*-10,11-Dichloro-10,11-dihydro-5H-dibenzo[a,d][7]annulen-5-one (2i)**

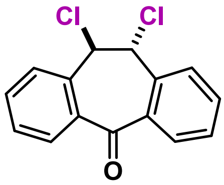

Pale yellow solid (131 mg, 95%). Mp: 182-184 ˚C. ^{1}H NMR (400 MHz, $CDCl_3$) δ 8.05 (dd, *J* = 7.7, 1.1 Hz, 2H), 7.57 (dt, *J* = 7.5, 1.4 Hz, 2H), 7.50 (dt, *J* = 7.5, 1.2 Hz, 2H), 7.43 (dd, *J* = 7.5, 1.1 Hz, 2H), 5.58 (s, 2H). ^{13}C NMR (101 MHz, $CDCl_3$) δ 192.8, 137.9, 135.4, 132.9, 131.5, 131.2, 129.8, 62.4. HRMS (Q-TOF): *m/z* $[M + H]^+$ calcd for $C_{15}H_{11}Cl_2O$: 277.0182, found: 277.0182.

2,3-Dichloro-1,3-diphenylpropan-1-one (2j)[7]

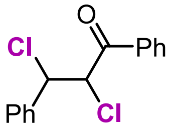

White solid (104 mg, 75%). Mp: 115-117 ˚C. ^{1}H NMR (400 MHz, $CDCl_3$) δ 8.15 – 8.02 (m, 2H), 7.71 – 7.63 (m, 1H), 7.60 – 7.50 (m, 4H), 7.51 – 7.37 (m, 3H), 5.51 (d, *J* = 10.6 Hz, 1H), 5.48 (d, *J* = 10.6 Hz, 1H). ^{13}C NMR (101 MHz, $CDCl_3$) δ 191.5, 137.2, 134.9, 134.4, 129.5, 129.2 (2C), 128.9, 128.5, 60.2, 57.2.

(1R(S),2S(R),4R(S),9R(S))-2,9-dichloro-1,2,3,4-tetrahydro-1,4-methanonaphthalene (2k)[9]

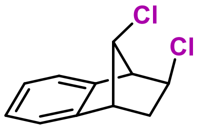

White solid (105 mg, 99%). Mp: 88-90 ˚C. ^{1}H NMR (400 MHz, $CDCl_3$) δ 7.25 – 7.12 (m, 4H), 4.12 – 4.08 (m, 1H), 3.86 (ddd, *J* = 7.9, 4.3, 1.1 Hz, 1H), 3.66 – 3.63 (m, 1H), 3.53 – 3.49 (m, 1H), 2.70 (dt, A part of AB system, *J* = 13.2, 4.0 Hz, 1H), 2.19 (dd, B part of AB system, *J* = 13.2, 7.9 Hz, 1H). ^{13}C NMR (101 MHz, $CDCl_3$) δ 144.0, 142.3, 128.0, 127.4, 122.1, 122.0, 66.4, 56.9, 56.9, 50.6, 36.4.

(1R(S),2S(R),4R(S),9R(S))-2,9-dichloro-5,8-dimethoxy-1,2,3,4-tetrahydro-1,4-methanonaphthalene (2l)

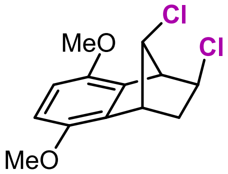

Brown oil (127 mg, 93%). ^{1}H NMR (400 MHz, $CDCl_3$) δ 6.65 (d, *J* = 9.0 Hz, 1H), 6.62 (d, *J* = 9.0 Hz, 1H), 4.08 – 4.04 (m, 1H), 3.87 – 3.82 (m, 2H), 3.78 (s, 3H), 3.76 (s, 3H), 3.70 – 3.66 (m, 1H), 2.65 (dt, A part of AB system, *J* = 13.2, 4.0 Hz, 1H), 2.18 (dd, B part of AB system, *J* = 13.2, 7.9 Hz, 1H). ^{13}C NMR (101

MHz, $CDCl_3$) δ 147.9, 147.7, 133.0, 131.0, 111.1, 110.2, 66.4, 56.6, 56.1, 55.9, 53.1, 47.1, 35.9. HRMS (Q-TOF): *m/z* $[M]^+$ calcd for $C_{13}H_{14}Cl_2O_2$: 272.0365, found: 272,0356.

(5R(S),6S(R),8R(S),10R(S))-6,10-dichloro-5,6,7,8-tetrahydro-5,8-methanonaphtho[2,3-d][1,3]dioxole (2m)

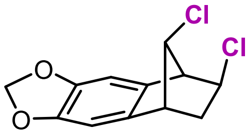

White solid (113 mg, 88%). Mp: 101-103 ˚C. ^1H NMR (400 MHz, $CDCl_3$) δ 6.73 (s, 1H), 6.69 (s, 1H), 5.92 (d, *J* = 1.3 Hz, 1H), 5.89 (d, *J* = 1.3 Hz, 1H), 4.09 – 4.04 (m, 1H), 3.80 (ddd, *J* = 7.9, 4.2, 1.2 Hz, 1H), 3.53 (s, 1H), 3.44 – 3.36 (m, 1H), 2.64 (dt, A part of AB system, *J* = 13.1, 3.9 Hz, 1H), 2.14 (dd, B part of AB system, *J* = 13.1, 8.0 Hz, 1H). HRMS (Q-TOF): *m/z* $[M\ ]^+$ calcd for $C_{12}H_{10}Cl_2O_2$: 256.0052, found: 256.0053.

(1S,2S,4S)-2-chloro-1-(chloromethyl)-7,7-dimethylbicyclo[2.2.1]heptane (2n)[10,11]

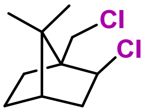

Colorless oil (77 mg, 80%). ^1H NMR (400 MHz, $CDCl_3$) δ 4.18 (dd, *J* = 8.5, 4.5 Hz, 1H), 3.95 (d, *J* = 10.8 Hz, 1H), 3.52 (d, *J* = 10.8 Hz, 1H), 2.29 – 2.18 (m, 1H), 2.08 (dd, *J* = 14.0, 8.6 Hz, 1H), 1.80 (m, 3H), 1.53 – 1.45 (m, 1H), 1.22 – 1.11 (m, 4H), 0.93 (s, 3H).

(1S,2R,4R)-2-chloro-1-(chloromethyl)-7,7-dimethylbicyclo[2.2.1]heptane (2o)[10,11]

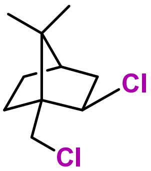

Colorless oil (77 mg, 80%). ^1H NMR (400 MHz, $CDCl_3$) δ 4.18 (dd, *J* = 8.5, 4.5 Hz, 1H), 3.95 (d, *J* = 10.8 Hz, 1H), 3.52 (d, *J* = 10.8 Hz, 1H), 2.29 – 2.18 (m, 1H), 2.08 (dd, *J* = 14.0, 8.6 Hz, 1H), 1.80 (m, 3H), 1.53 – 1.45 (m, 1H), 1.22 – 1.11 (m, 4H), 0.93 (s, 3H). ^{13}C NMR (101 MHz, $CDCl_3$) 64.3, 54.3, 48.8, 47.6, 46.1, 41.8, 33.0, 26.6, 21.1, 20.4.

(S)-5-(3-chloroprop-1-en-2-yl)-2-methylcyclohex-2-en-1-one (2p)[12]

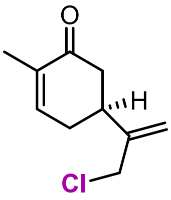

Colorless oil (55 mg, 60%). ^1H NMR (400 MHz, $CDCl_3$) δ 6.79 – 6.73 (m, 1H), 5.26 (s, 1H), 5.06 (d, *J* = 1.3 Hz, 1H), 4.09 (s, 2H), 3.07 – 2.88 (m, 1H), 2.72 – 2.19 (m, 4H), 1.82 – 1.77 (m, 3H). ^{13}C NMR (101 MHz, $CDCl_3$) δ 199.1, 146.8, 144.2, 135.8, 115.3, 47.1, 43.2, 38.0, 31.6, 15.8.

(R)-5-(3-chloroprop-1-en-2-yl)-2-methylcyclohex-2-en-1-one (2q)[12]

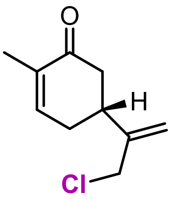

Colorless oil (57 mg, 62%). ^{1}H NMR (400 MHz, $CDCl_3$) δ 6.79 – 6.73 (m, 1H), 5.26 (s, 1H), 5.06 (d, *J* = 1.3 Hz, 1H), 4.09 (s, 2H), 3.07 – 2.88 (m, 1H), 2.72 – 2.19 (m, 4H), 1.82 – 1.77 (m, 3H). ^{13}C NMR (101 MHz, $CDCl_3$) δ 199.1, 146.8, 144.2, 135.8, 115.3, 47.1, 43.2, 38.0, 31.6, 15.8.

1,2-Dibromooctane (3a)[13]

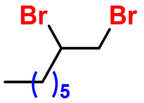

Pale yellow oil (133 mg, 98%). ^{1}H NMR (400 MHz, $CDCl_3$) δ 4.25 – 4.11 (m, 1H), 3.85 (dd, *J* = 10.3, 4.4 Hz, 1H), 3.63 (t, *J* = 9.9 Hz, 1H), 2.19 – 2.08 (m, 1H), 1.88 – 1.68 (m, 1H), 1.64 – 1.51 (m, 1H), 1.49 – 1.22 (m, 7H), 0.89 (t, *J* = 6.8 Hz, 3H). ^{13}C NMR (101 MHz, $CDCl_3$) δ 53.3, 36.5, 36.2, 31.7, 28.6, 26.9, 22.7, 14.2.

***trans*-1,2-Dibromocyclohexane (3b)[13]**

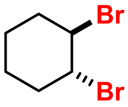

Colorless oil (115 mg, 95%). ^{1}H NMR (400 MHz, $CDCl_3$) δ 4.45 (s, 2H), 2.52 – 2.38 (m, 2H), 1.96 – 1.73 (m, 4H), 1.60 – 1.44 (m, 2H). ^{13}C NMR (101 MHz, $CDCl_3$) δ 55.3, 32.1, 22.5.

***trans*-2,3-Dibromo-1,2,3,4-tetrahydronaphthalene (3c)[14]**

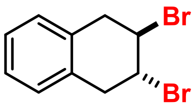

White solid (140 mg, 97%). Mp: 65-67 ˚C. ^{1}H NMR (400 MHz, $CDCl_3$) δ 7.25 – 7.19 (AA' part of AA'BB' system, 2H), 7.14 – 7.09 (BB' part of AA'BB' system, 2H), 4.81 – 4.72 (m, 2H), 3.99 (dd, A part of AB system, *J* = 17.8, 3.1 Hz, 2H), 3.29 (d, B part of AB system, *J* = 17.8 Hz, 2H). ^{13}C NMR (101 MHz, $CDCl_3$) δ 130.8, 129.2, 126.9, 49.3, 34.7.

(1,2-Dibromoethyl)benzene (3d)[13]

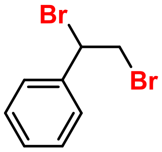

White solid (125 mg, 95%). Mp: 73-75 ˚C. ^{1}H NMR (400 MHz, $CDCl_3$) δ 7.48 – 7.31 (m, 5H), 5.15 (dd, *J* = 10.6, 5.5 Hz, 1H), 4.23 – 3.97 (m, 2H). ^{13}C NMR (101 MHz, $CDCl_3$) δ 138.8, 129.3, 129.0, 127.8, 77.5, 77.2, 76.8, 51.0, 35.2.

1-(tert-butyl)-4-(1,2-dibromoethyl)benzene (3e)[13]

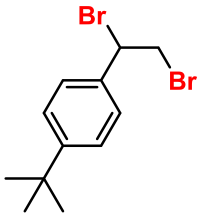

White solid (150 mg, 94%). Mp: 60-62 ˚C. ^{1}H NMR (400 MHz, $CDCl_3$) δ 7.40 (d, *J* = 8.5 Hz, 2H), 7.34 (d, *J* = 8.5 Hz, 2H), 5.17 (dd, *J* = 10.2, 5.7 Hz, 1H), 4.12 – 4.00 (m, 2H), 1.33 (s, 9H). ^{13}C NMR (101 MHz, $CDCl_3$) δ 152.4, 135.7, 127.4, 126.0, 51.4, 35.3, 34.9, 31.4.

4-(1,2-Dibromoethyl)phenyl acetate (3f)[15]

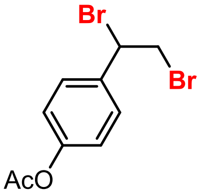

White solid (148, 92%). Mp: 90-92 ˚C. ^{1}H NMR (400 MHz, $CDCl_3$) δ 7.42 (d, *J* = 8.6 Hz, 2H), 7.12 (d, *J* = 8.6 Hz, 2H), 5.14 (dd, *J* = 10.7, 5.3 Hz, 1H), 4.07 (dd, *J* = 10.3, 5.3 Hz, 1H), 3.98 (t, *J* = 10.5 Hz, 1H), 2.31 (s, 3H). ^{13}C NMR (101 MHz, $CDCl_3$) δ 169.2, 151.1, 136.2, 129.0, 122.1, 50.2, 35.1, 21.3.

(1R,2R)-1,2-dibromo-2,3-dihydro-1H-indene (3g)[16]

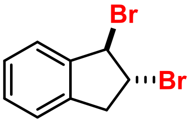

White solid (132 mg, 96%). Mp: 29-31 ˚C. ^{1}H NMR (400 MHz, $CDCl_3$) δ 7.49 – 7.45 (m, 1H), 7.38 – 7.28 (m, 3H), 5.63 (s, 1H), 4.91 – 4.80 (m, 1H), 3.82 (dd, *J* = 17.5, 5.2 Hz, 1H), 3.27 (d, *J* = 17.5 Hz, 1H). ^{13}C NMR (101 MHz, $CDCl_3$) δ 140.7, 129.9, 128.1, 125.9, 125.6, 57.9, 54.6, 41.6 (2C signal overlaps).

(*E*)-1,2-Dibromo-1,2-diphenylethene (3h)[17]

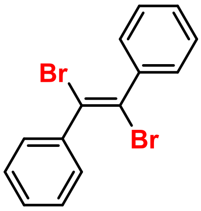

White solid (178 mg, 97%). Mp: 215-217 ˚C. ^{1}H NMR (400 MHz, $CDCl_3$) δ 7.58 – 7.49 (m, 4H), 7.48 – 7.32 (m, 6H). ^{13}C NMR (101 MHz, $CDCl_3$) δ 140.9, 129.2, 129.1, 128.5, 118.2.

***trans*-10,11-Dibromo-10,11-dihydro-5H-dibenzo[a,d][7]annulen-5-one (3i)[18]**

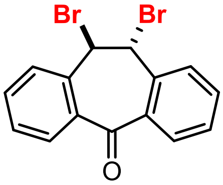

Colorless solid (180 mg, 98%). Mp: 204-206 ˚C. ^{1}H-NMR (400 MHz, $CDCl_3$): δ = 8.09 (dd, *J*=7.9Hz, *J*=1.5Hz, 2H), 7.57 (dt, *J*=7.3Hz, *J*=1.5Hz, 2H), 7.50 (dt, *J*=7.9Hz, *J*=1.5Hz, 2H), 7.41 (dd, *J*=7.3Hz, *J*=1.5Hz, 2H), 5.80 (s, 2H). ^{13}C-NMR (100 MHz, $CDCl_3$): δ = 192.3, 138.1, 136.8, 132.9, 131.6, 131.1, 129.7, 52.9.

2,3-Dibromo-1,3-diphenylpropan-1-one (3j)[4]

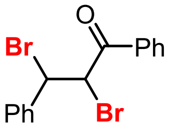

White solid (171 mg, 93%). Mp: 160-162 ˚C. ^{1}H NMR (400 MHz, $CDCl_3$) δ 8.17 – 8.06 (m, 2H), 7.72 – 7.62 (m, 1H), 7.60 – 7.51 (m, 4H), 7.47 – 7.36 (m, 3H), 5.84 (d, *J* = 11.4 Hz, 1H), 5.65 (d, *J* = 11.4 Hz, 1H). ^{13}C NMR (101 MHz, $CDCl_3$) δ 191.3, 138.4, 134.5, 134.4, 129.5, 129.2, 129.1, 129.0, 128.5, 49.9, 47.0.

(1R(S),2S(R),4R(S),9R(S))-2,9-dibromo-1,2,3,4-tetrahydro-1,4-methanonaphthalene (3k)[19]

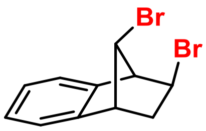

White solid (149 mg, 99%). Mp: 76-78 ˚C. ^{1}H NMR (400 MHz, $CDCl_3$) δ 7.25 – 7.12 (m, 4H), 4.18 – 4.13 (m, 1H), 3.80 (ddd, *J* = 8.0, 4.6, 1.3 Hz, 1H), 3.75 (bs, 1H), 3.54 – 3.49 (m, 1H), 2.87 (dt, A part of AB system, *J* = 13.4, 4.2 Hz, 1H), 2.21 (dd, B part of AB system, *J* = 13.4, 8.0 Hz, 1H). ^{13}C NMR (101 MHz, $CDCl_3$) δ 143.7, 143.1, 128.0, 127.4, 121.9, 121.5, 56.6, 55.7, 51.2, 45.2, 36.7.

(1R(S),2S(R),4R(S),9R(S))-2,9-dibromo-5,8-dimethoxy-1,2,3,4-tetrahydro-1,4-methanonaphthalene (3l)

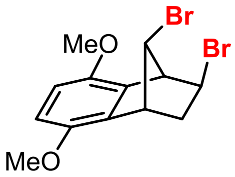

Pale yellow oil (175 mg, 97%). ^{1}H NMR (400 MHz, $CDCl_3$) δ 6.65 (d, *J* = 9.0 Hz, 1H), 6.61 (d, *J* = 9.0 Hz, 1H), 4.13 – 4.09 (m, 1H), 3.97 – 3.94 (m, 1H), 3.83 – 3.77 (m, 4H), 3.76 (s, 3H), 3.73 – 3.67 (m, 1H), 2.83 (dt, A part of AB system, *J* = 13.4, 4.1 Hz, 1H), 2.20 (dd, B part of AB system, *J* = 13.4, 8.0 Hz, 1H). ^{13}C NMR (101 MHz, $CDCl_3$) δ 147.8, 147.3, 132.7, 131.9, 111.1, 110.3, 56.1, 55.9, 55.7, 52.8, 47.7, 45.1, 36.2. HRMS (Q-TOF): *m/z* [M]$^+$ calcd for $C_{13}H_{14}Br_2O_2$: 359.9355, found: 359.9355.

(5R(S),6S(R),8R(S),10R(S))-6,10-dibromo-5,6,7,8-tetrahydro-5,8-methanonaphtho[2,3-d][1,3]dioxole (3m)

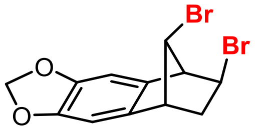

White solid (152 mg, 88%). Mp: 94-96 ˚C. ^{1}H NMR (400 MHz, $CDCl_3$) δ 6.73 (s, 1H), 6.69 (s, 1H), 5.93 (d, *J* = 1.3 Hz, 1H), 5.90 (d, *J* = 1.3 Hz, 1H), 4.14 – 4.10 (m, 1H), 3.74 (ddd, *J* = 8.0, 4.6, 1.3 Hz, 1H), 3.65 (bs, 1H), 3.44 – 3.37 (m, 1H), 2.82 (dt, A part of AB system, *J* = 13.2, 4.2 Hz, 1H), 2.16 (dd, B part of AB system, *J* = 13.2, 8.0 Hz, 1H). ^{13}C NMR (101 MHz, $CDCl_3$) δ 147.1, 146.6, 137.2, 136.2, 103.9, 103.3, 101.1, 56.5, 55.1, 51.1, 45.3, 36.8. HRMS (Q-TOF): *m/z* [M]$^+$ calcd for $C_{12}H_{10}Br_2O_2$: 343.9042, found: 343.9043.

(1S,2S,4S)-2-bromo-1-(bromomethyl)-7,7-dimethylbicyclo[2.2.1]heptane (3n)[20]

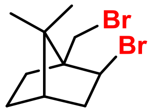

Brown solid (127 mg, 90%). Mp: 63-65 ˚C. ^{1}H NMR (400 MHz, $CDCl_3$) δ 4.26 (dd, *J* = 8.6, 4.6 Hz, 1H), 3.77 (d, *J* = 9.9 Hz, 1H), 3.48 (d, *J* = 9.9 Hz, 1H), 2.43 (ddd, *J* = 14.3, 7.9, 4.6 Hz, 1H), 2.16 (dd, *J* = 14.3, 8.6 Hz, 1H), 1.99 (t, *J* = 4.4 Hz, 1H), 1.93 (dd, *J* = 13.1, 4.4 Hz, 1H), 1.79 (dtd, *J* = 11.9, 7.5, 4.1 Hz, 1H), 1.61 – 1.51 (m, 2H), 1.21 (s, 3H), 0.94 (s, 3H).^{13}C NMR (101 MHz, $CDCl_3$) δ 56.8, 53.2, 49.5, 48.4, 42.2, 37.4, 34.6, 26.5, 21.1, 20.5.

(1S,2R,4R)-2-bromo-1-(bromomethyl)-7,7-dimethylbicyclo[2.2.1]heptane (3o)[20]

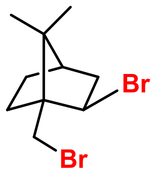

Brown solid (127 mg, 90%). Mp: 63-65 ˚C. ^{1}H NMR (400 MHz, $CDCl_3$) δ 4.26 (dd, *J* = 8.6, 4.6 Hz, 1H), 3.77 (d, *J* = 9.9 Hz, 1H), 3.48 (d, *J* = 9.9 Hz, 1H), 2.43 (ddd, *J* = 14.3, 7.9, 4.6 Hz, 1H), 2.16 (dd, *J* = 14.3, 8.6 Hz, 1H), 1.99 (t, *J* = 4.4 Hz, 1H), 1.93 (dd, *J* = 13.1, 4.4 Hz, 1H), 1.79 (dtd, *J* = 11.9, 7.5, 4.1 Hz, 1H), 1.61 – 1.51 (m, 2H), 1.21 (s, 3H), 0.94 (s, 3H). ^{13}C NMR (101 MHz, $CDCl_3$) δ 56.8, 53.2, 49.5, 48.4, 42.2, 37.4, 34.6, 26.5, 21.1, 20.5.

Mixture of (S)-5-((R)-1,2-dibromopropan-2-yl)-2-methylcyclohex-2-en-1-one (3p) and (S)-5-((S)-1,2-dibromopropan-2-yl)-2-methylcyclohex-2-en-1-one (3q)[4]

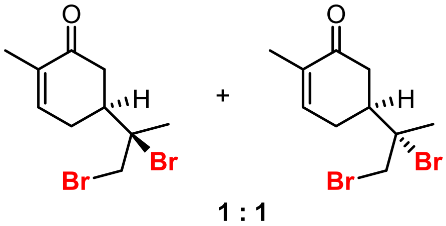

Colorless oil (142 mg, 92%). ^{1}H NMR (400 MHz, $CDCl_3$) δ 6.79 – 6.70 (m, 2H), 3.99 – 3.91 (m, 2H), 3.85 (d, *J* = 10.4 Hz, 1H), 3.81 (d, *J* = 10.5 Hz, 1H), 2.74 – 2.25 (m, 10H), 1.88 (s, 3H), 1.86 (s, 3H), 1.80 (s, 3H), 1.79 (s, 3H). ^{13}C NMR (101 MHz, $CDCl_3$) δ 198.6, 198.2, 143.8, 143.5, 135.5, 135.4, 71.2, 71.1, 42.4, 42.1, 40.8, 40.73, 40.68, 40.1, 28.9 (2C), 28.5, 28.0, 15.72, 15.71.

Mixture of (R)-5-((R)-1,2-dibromopropan-2-yl)-2-methylcyclohex-2-en-1-one (3r) and (R)-5-((S)-1,2-dibromopropan-2-yl)-2-methylcyclohex-2-en-1-one (3s)[4]

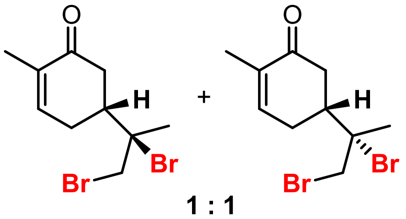

Colorless oil (142 mg, 92%). ^{1}H NMR (400 MHz, $CDCl_3$) δ 6.81 – 6.71 (m, 2H), 4.00 – 3.92 (m, 2H), 3.86 (d, *J* = 10.4 Hz, 1H), 3.82 (d, *J* = 10.5 Hz, 1H), 2.72 – 2.30 (m, 10H), 1.90 (s, 3H), 1.88 (s, 3H), 1.80 (s, 3H), 1.79 (s, 3H). ^{13}C NMR (101 MHz, $CDCl_3$) δ 198.6, 198.2, 143.8, 143.6, 135.5, 135.4, 71.2, 71.1, 42.4, 42.1, 40.8, 40.72, 40.67, 40.1, 28.9 (2C), 28.4, 28.0, 15.73, 15.71.

Dibromo-cholesterol (3t)[21]

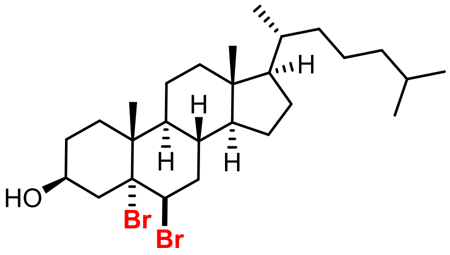

Brown viscous (88%). ^{1}H NMR (400 MHz, $CDCl_3$) δ 4.85 (dd, *J* = 12.5, 5.0 Hz, 1H), 4.24 – 4.15 (m, 1H), 3.16 (bs, 1H), 2.82 (d, *J* = 16.6 Hz, 1H), 2.49 (dd, *J* = 16.6, 4.5 Hz, 1H), 2.29 – 2.19 (m, 1H), 2.12 – 0.58

(m, 40H). ^{13}C NMR (101 MHz, $CDCl_3$) δ 89.8, 69.2, 56.2, 56.1, 55.2, 47.4, 45.8, 42.7, 41.9, 39.6, 39.5, 37.2, 36.7, 36.1, 35.8, 30.8, 30.2, 28.2, 28.0, 24.1, 23.8, 22.8, 22.6, 21.3, 20.4, 18.7, 12.2.

References

1. Agarwal, J., & Peddinti, R. K. (2019). Water-Mediated, Highly-Efficient and Improved Protocol for the Synthesis of Vesamicol, Its Analogues and β-Blockers through the Highly-Chemoselective Aminolysis of Epoxides. *ChemistrySelect*, *4*(26), 7745-7750.
2. Goll, J. M., & Fillion, E. (2008). Tuning the reactivity of palladium carbenes derived from diphenylketene. *Organometallics*, *27*(14), 3622-3625.
3. Sun, X., Bai, X. Y., Li, A. Z., & Li, B. J. (2021). Iridium-Catalyzed Asymmetric Hydroalkenylation of Norbornene Derivatives. *Organometallics*, *40*(14), 2182-2187.
4. Stodulski, M., Goetzinger, A., Kohlhepp, S. V., & Gulder, T. (2014). Halocarbocyclization versus dihalogenation: substituent directed iodine (III) catalyzed halogenations. *Chemical Communications*, *50*(26), 3435-3438.
5. Wedek, V., Van Lommel, R., Daniliuc, C. G., De Proft, F., & Hennecke, U. (2019). Organocatalytic, enantioselective dichlorination of unfunctionalized alkenes. *Angewandte Chemie International Edition*, *58*(27), 9239-9243.
6. Fu, N., Sauer, G. S., & Lin, S. (2017). Electrocatalytic radical dichlorination of alkenes with nucleophilic chlorine sources. *Journal of the American Chemical Society*, *139*(43), 15548-15553.
7. Lian, P., Long, W., Li, J., Zheng, Y., & Wan, X. (2020). Visible-Light-Induced Vicinal Dichlorination of Alkenes through LMCT Excitation of CuCl2. *Angewandte Chemie*, *132*(52), 23809-23814.
8. Sohmiya, H., Kimura, T., Fujita, M., & Ando, T. (1998). Solid-state organic reactions proceeding by pulverization: Oxidation and halogenation with iodosobenzene and inorganic solid-supports. *Tetrahedron*, *54*(45), 13737-13750.
9. Post, A. J., Nash, J. J., Love, D. E., Jordan, K. D., & Morrison, H. (1995). Photochemical Activation of Distal Functional-Groups in Polyfunctional Molecules-Photochemistry and Photophysics of the Syn-7-Chlorobenzonorbornenes and Anti-7-Chlorobenzonorbornenes. *Journal of the American Chemical Society*, *117*(17), 4930-4935.
10. Moreno-Dorado, F. J., Guerra, F. M., Manzano, F. L., Aladro, F. J., Jorge, Z. D., & Massanet, G. M. (2003). CeCl3/NaClO: a safe and efficient reagent for the allylic chlorination of terminal olefins. *Tetrahedron letters*, *44*(35), 6691-6693.
11. Lezina, O. M., Grebyonkina, O. N., Patov, S. A., Rubtsova, S. A., & Kutchin, A. V. (2014). Oxidative transformations of diisobornyl disulfide. *Russian Chemical Bulletin*, *63*(9), 2067-2073.
12. Borade, B. R., Dixit, R., & Kontham, R. (2020). Total Synthesis of Beshanzuenone D and Its Epimers and Abiespiroside A. *Organic Letters*, *22*(21), 8561-8565.
13. Martins, N. S., & Alberto, E. E. (2018). Dibromination of alkenes with LiBr and H 2 O 2 under mild conditions. *New Journal of Chemistry*, *42*(1), 161-167.
14. EŞSİZ, S., & DAŞTAN, A. (2019). 1, 2-Dibromotetrachloroethane: an efficient reagent for many transformations by modified Appel reaction. *Turkish Journal of Chemistry*, *43*(1), 150-156.
15. Yuan, Y., Yao, A., Zheng, Y., Gao, M., Zhou, Z., Qiao, J., ... & Lei, A. (2019). Electrochemical oxidative clean halogenation using HX/NaX with hydrogen evolution. *Iscience*, *12*, 293-303.
16. Ng, W. H., Shing, T. K., & Yeung, Y. Y. (2018). Mild and Efficient Vicinal Dibromination of Olefins Mediated by Aqueous Ammonium Fluoride. *Synlett*, *29*(04), 419-424.
17. Podgoršek, A., Eissen, M., Fleckenstein, J., Stavber, S., Zupan, M., & Iskra, J. (2009). Selective aerobic oxidative dibromination of alkenes with aqueous HBr and sodium nitrite as a catalyst. *Green Chemistry*, *11*(1), 120-126.
18. Koçak, R., & Daştan, A. (2021). Synthesis of dibenzosuberenone-based novel polycyclic π-conjugated dihydropyridazines, pyridazines and pyrroles. *Beilstein journal of organic chemistry*, *17*(1), 719-729.
19. Dastan, A., Demir, U., & Balci, M. (1994). Functionalization of benzonorbornadiene: high-temperature bromination and electrochemical oxidation. *The Journal of Organic Chemistry*, *59*(22), 6534-6538.

20. Zyk, N. V., Beloglazkina, E. K., Tyurin, V. S., & Zefirov, N. S. (1998). A New Method For Mıxed Halogenatıon. N-Chloroamıne-Phosphorus Bromıde System As A Synthetıc Equıvalent Of The Mıxed Halogen $Cl^{+}Br^{-}$. *Phosphorus, Sulfur, and Silicon and the Related Elements*, *139*(1), 107-122.
21. Fieser, L. F. (1953). Cholesterol and companions. VII. Steroid dibromides. *Journal of the American Chemical Society*, *75*(21), 5421-5422.

.

^{1}H NMR, ^{13}C NMR, and HRMS Spectra

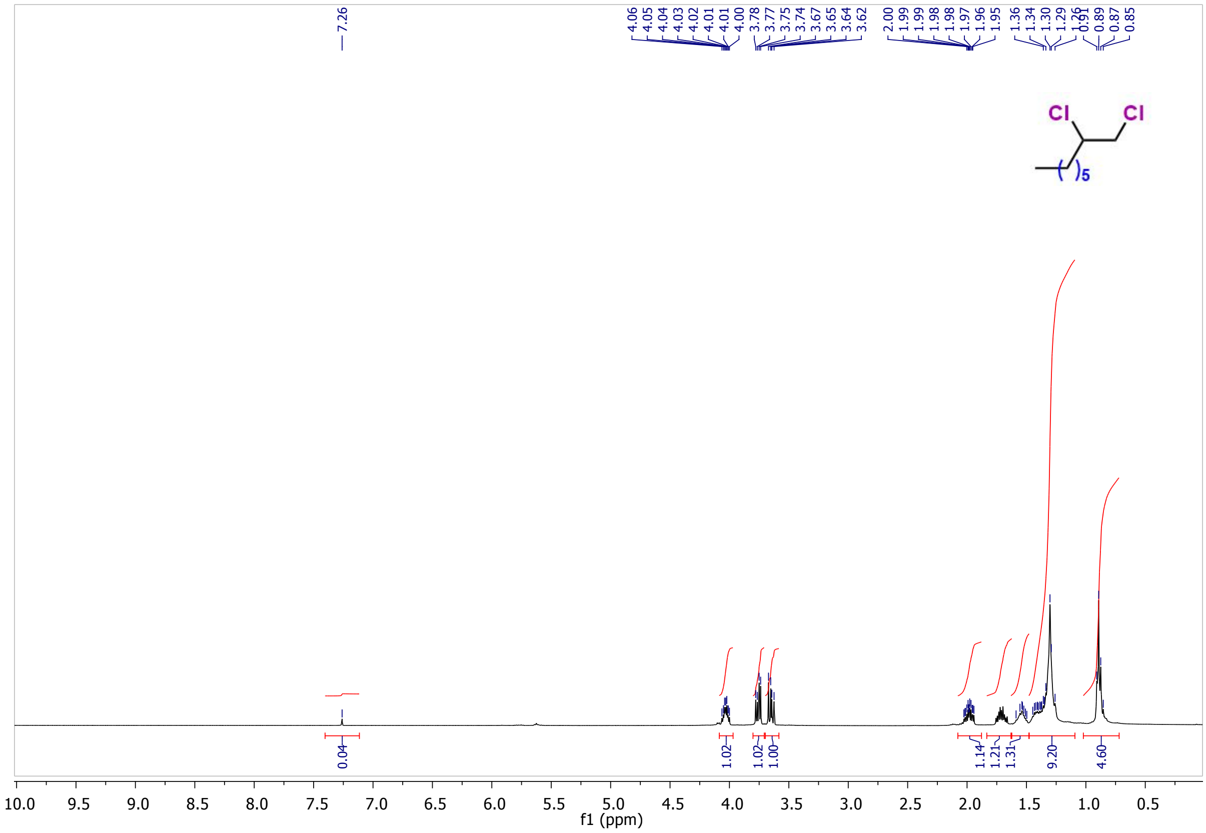

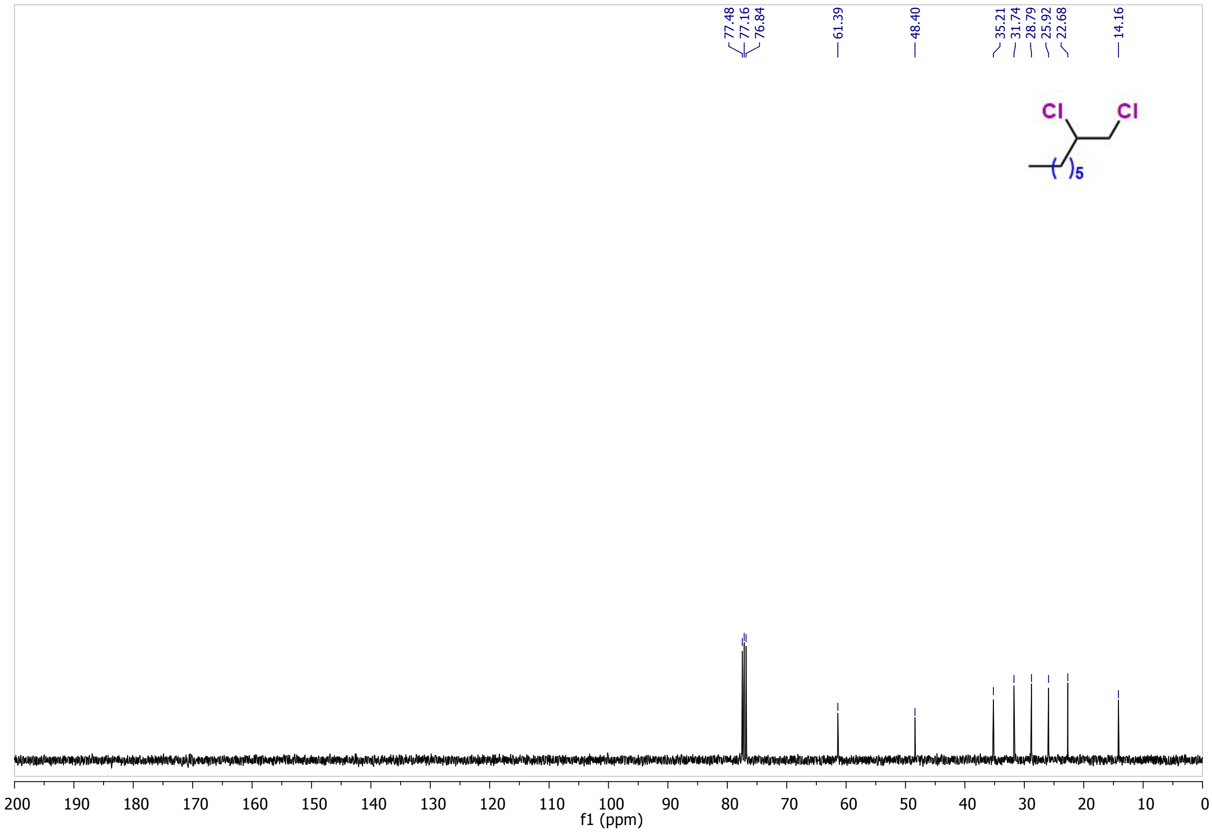

400 MHz ^{1}H-NMR (top) and 101 MHz ^{13}C-NMR (bottom) spectra of **2a** ($CDCl_3$)

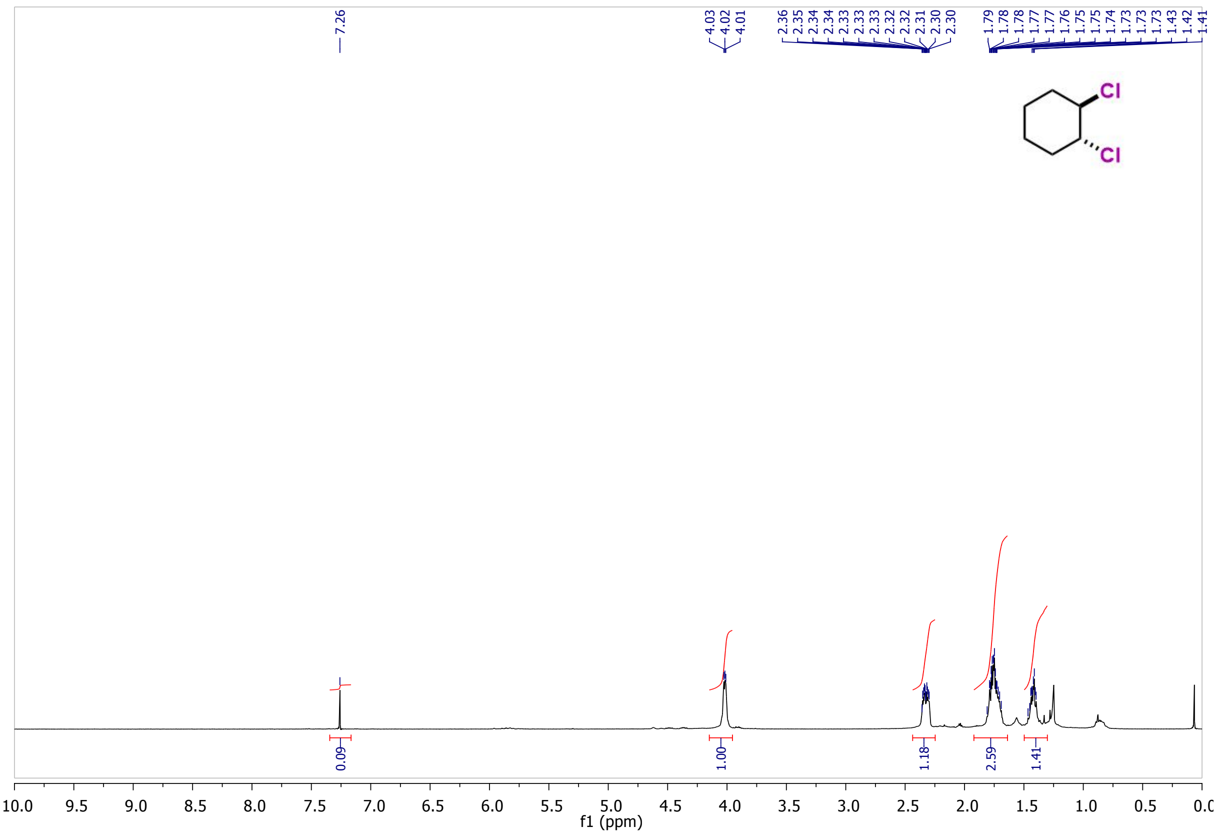

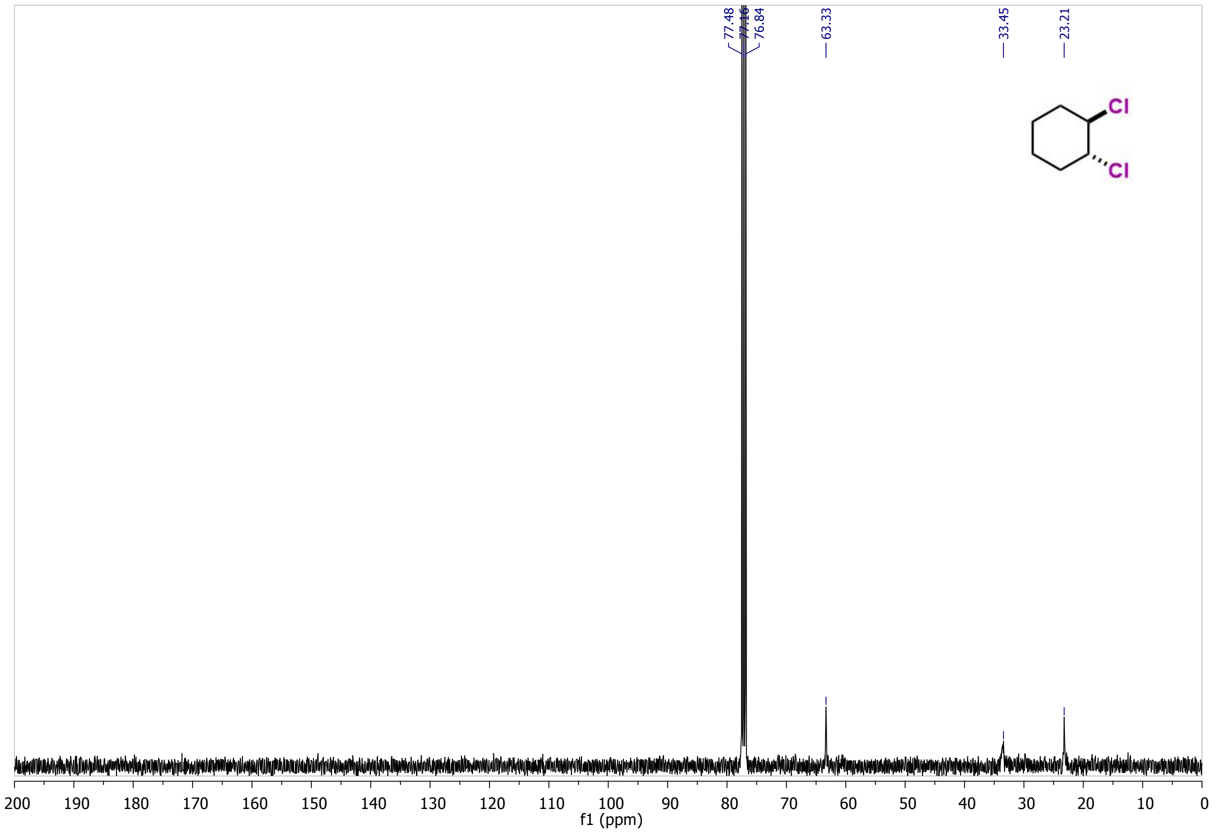

400 MHz ^{1}H-NMR (top) and 101 MHz ^{13}C-NMR (bottom) spectra of **2b** ($CDCl_3$)

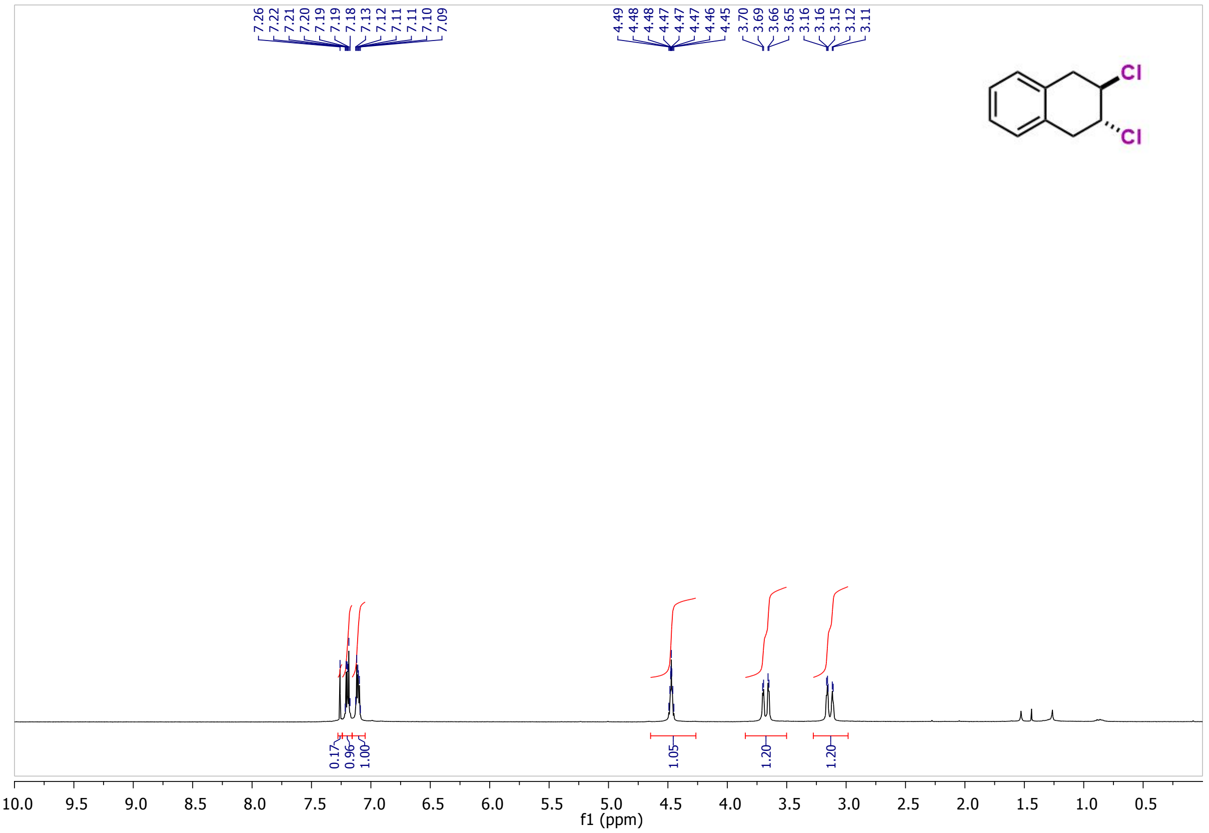

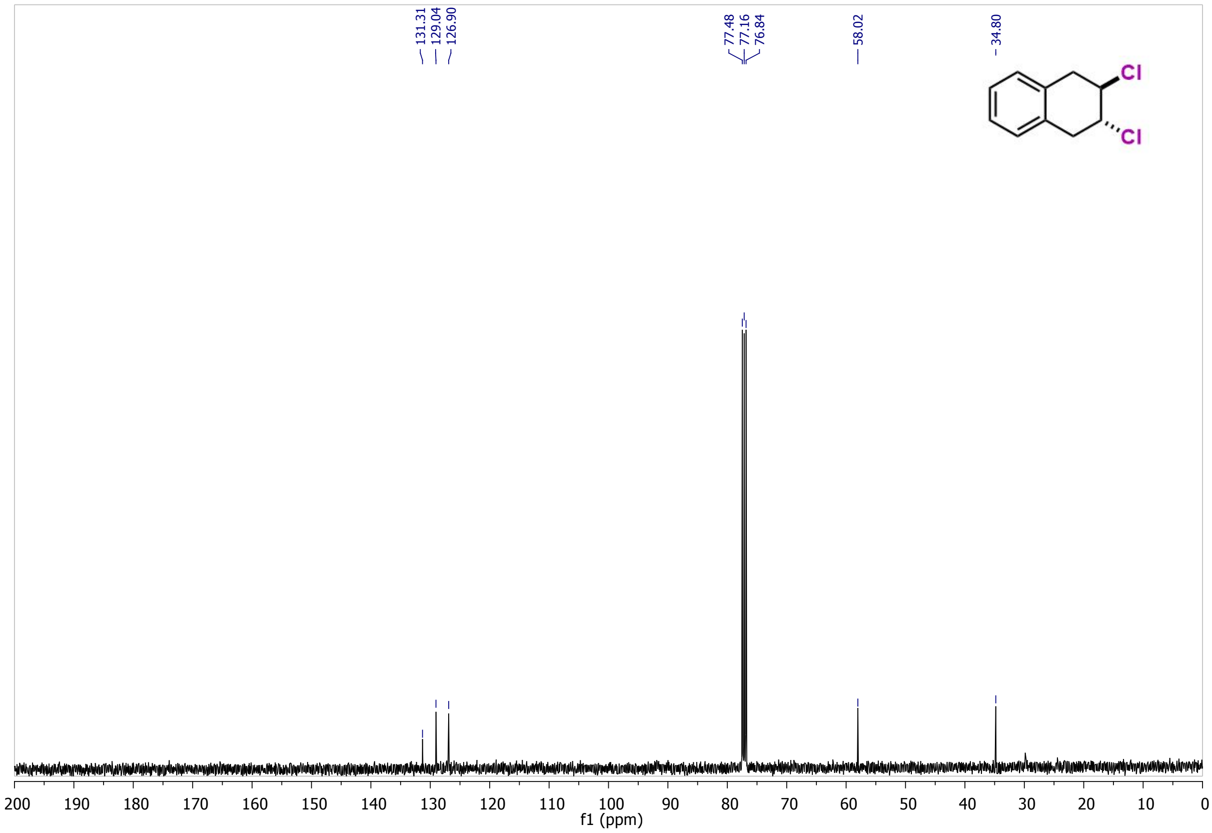

400 MHz ^{1}H-NMR (top) and 101 MHz ^{13}C-NMR (bottom) spectra of **2c** ($CDCl_3$)

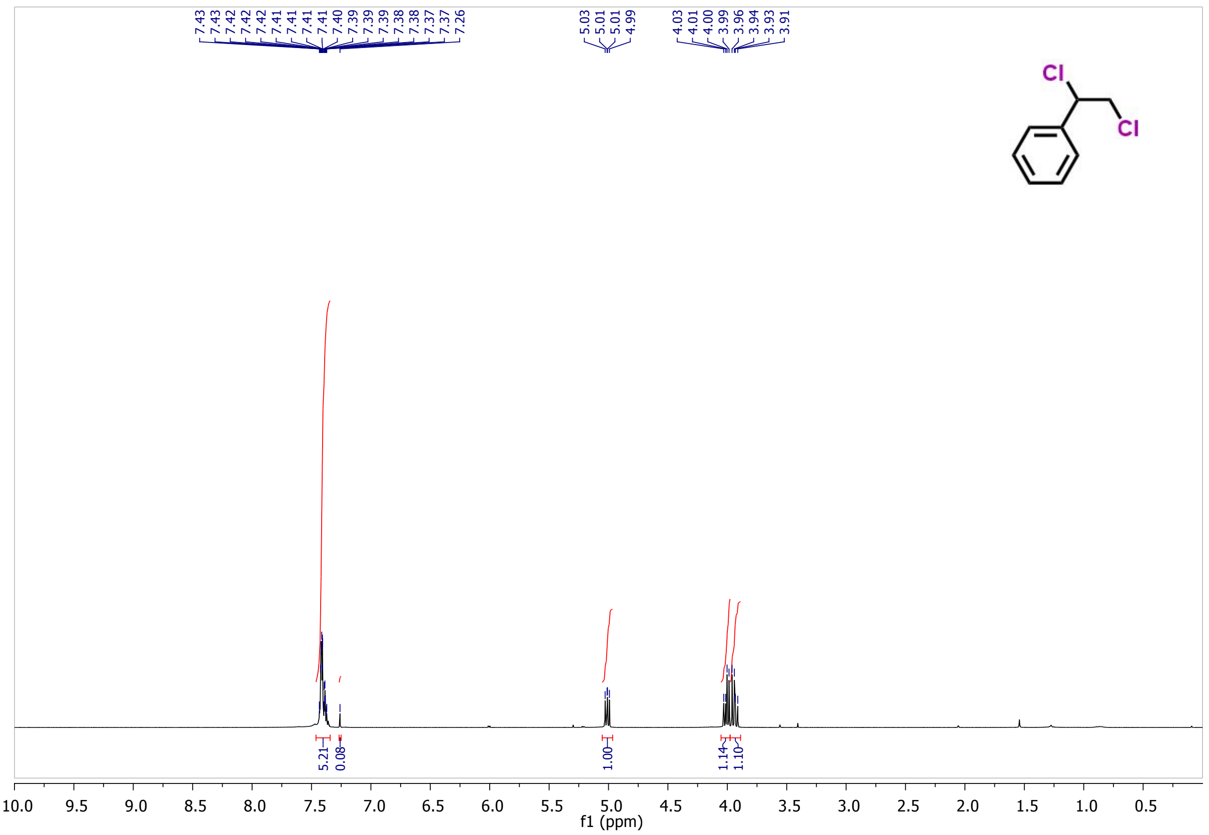

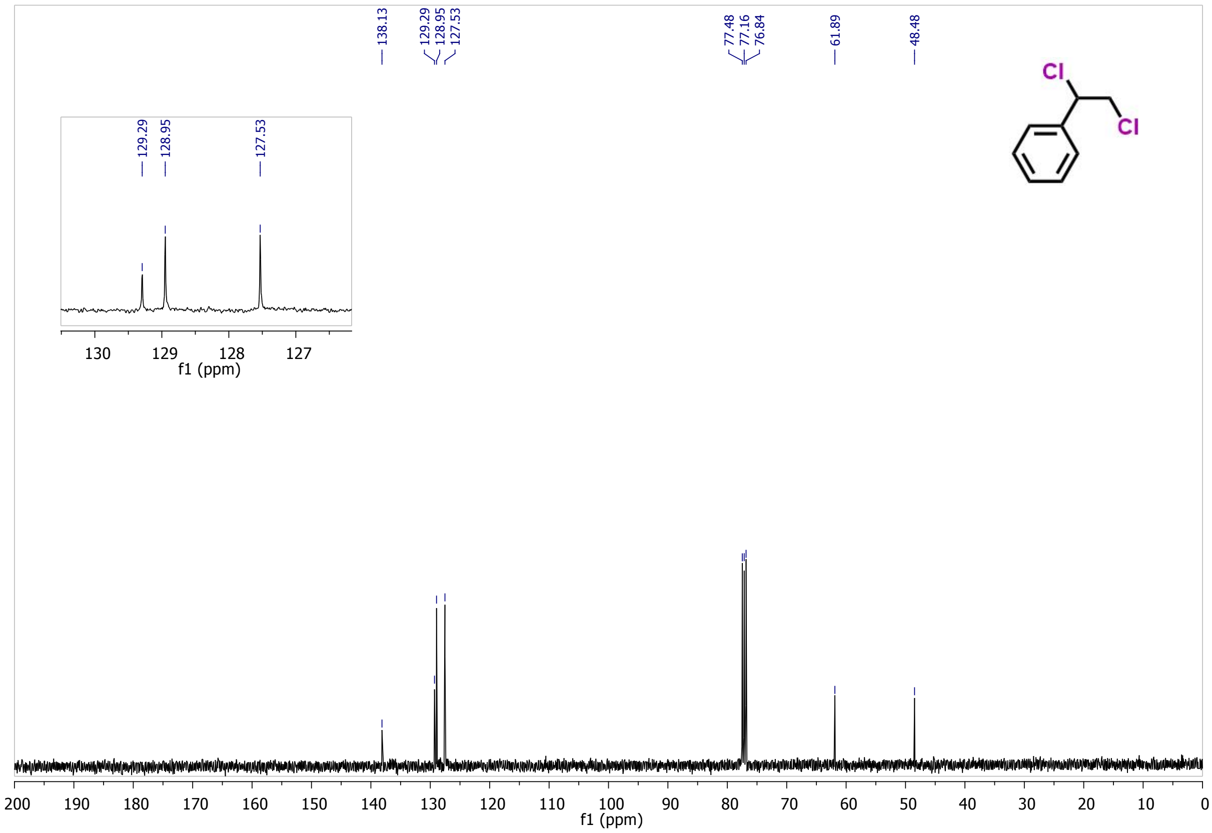

400 MHz ^{1}H-NMR (top) and 101 MHz ^{13}C-NMR (bottom) spectra of **2d** ($CDCl_3$)

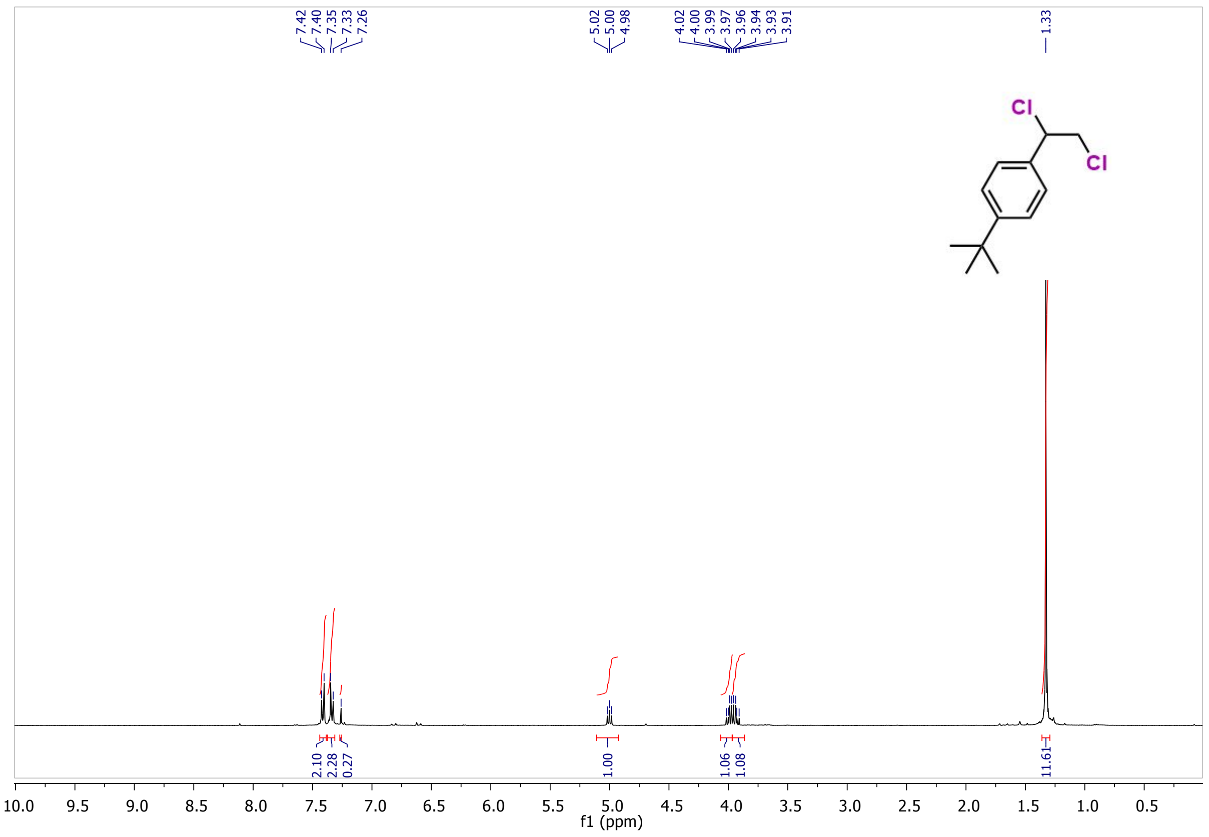

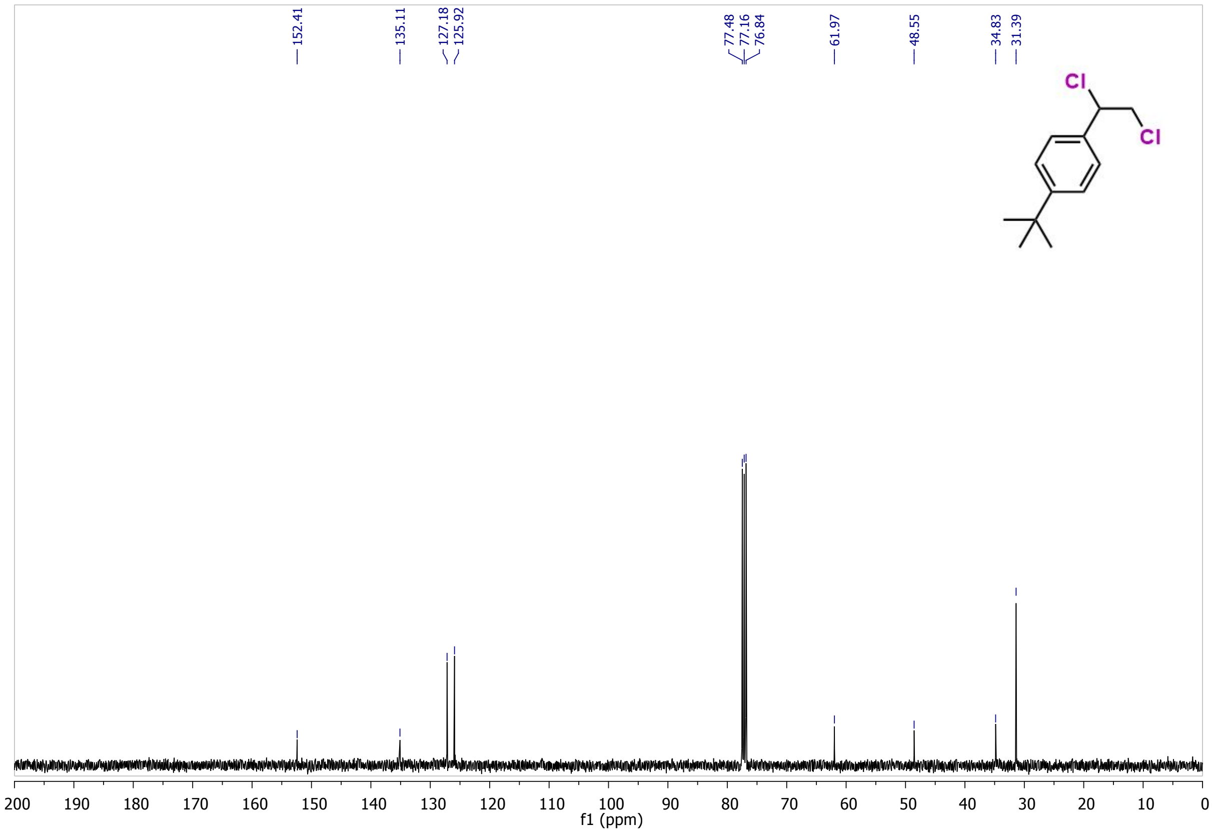

400 MHz ^{1}H-NMR (top) and 101 MHz ^{13}C-NMR (bottom) spectra of **2e** ($CDCl_3$)

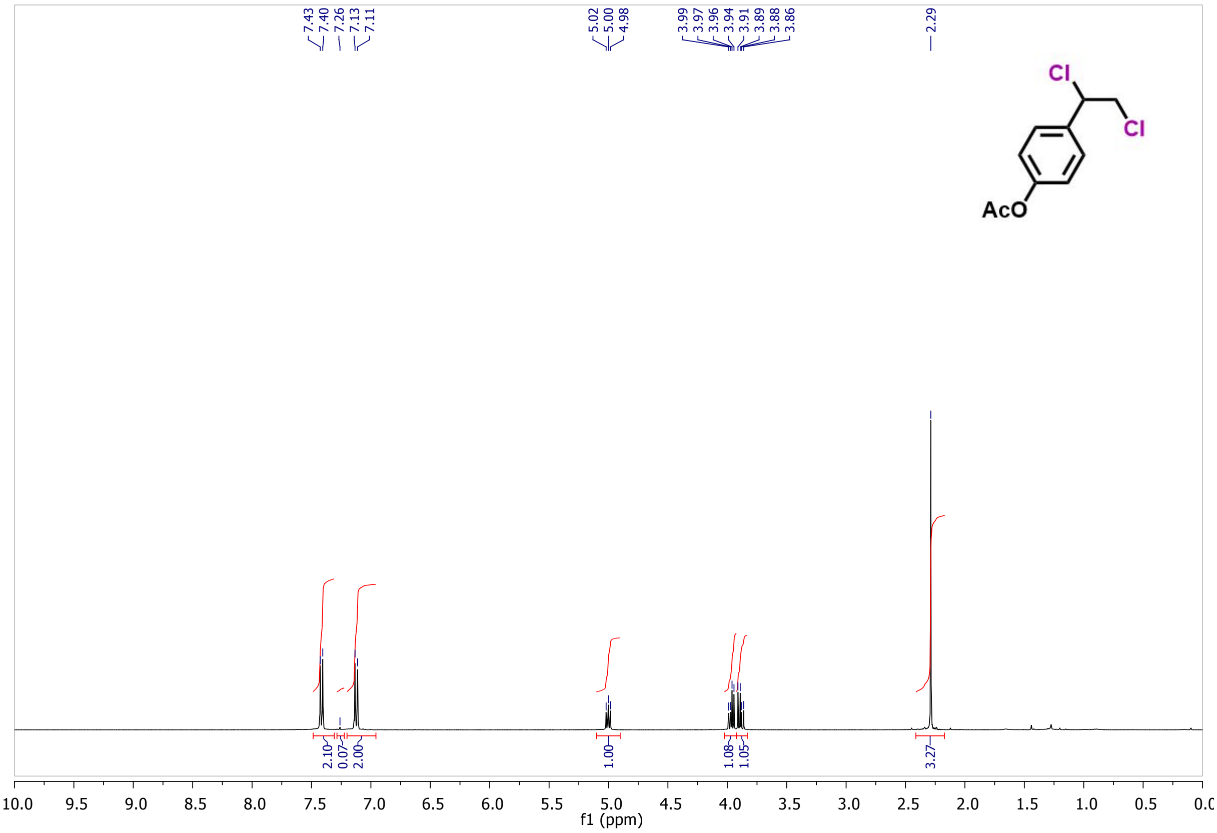

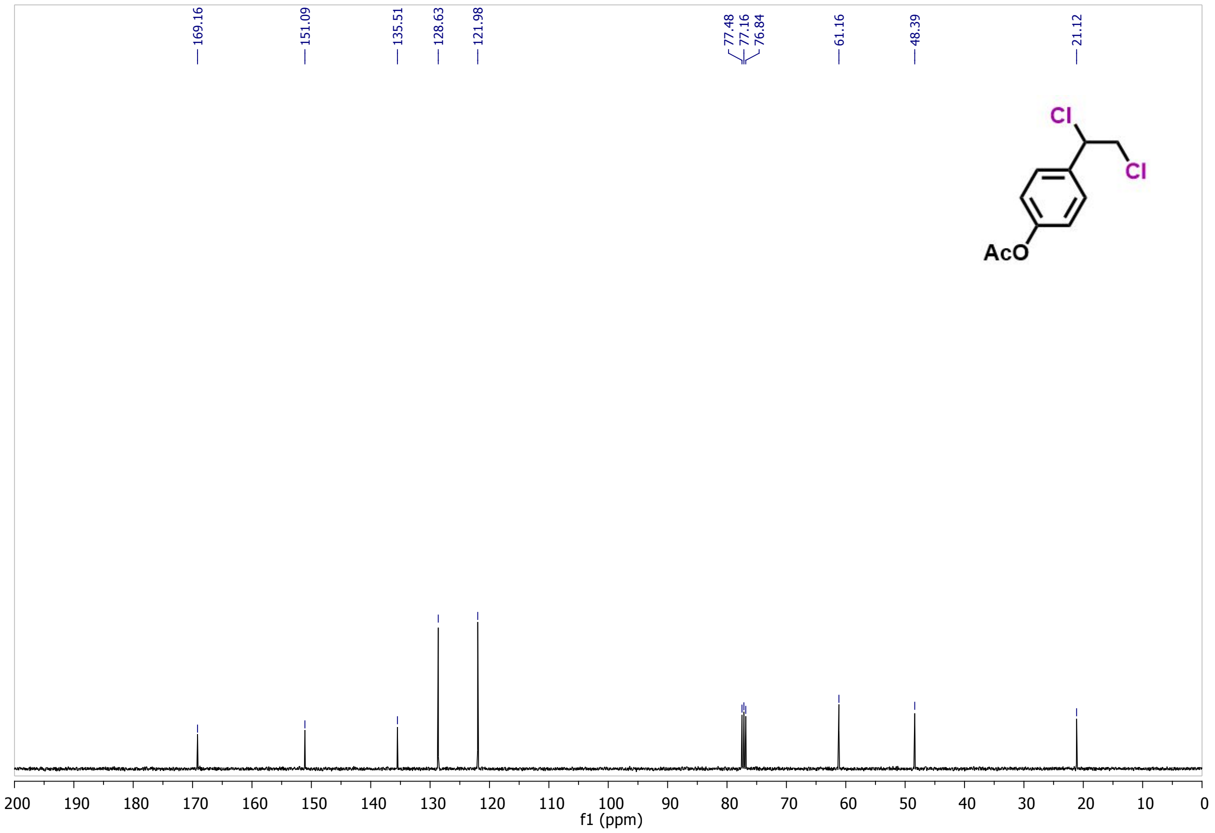

400 MHz ^{1}H-NMR (top) and 101 MHz ^{13}C-NMR (bottom) spectra of **2f** ($CDCl_3$)

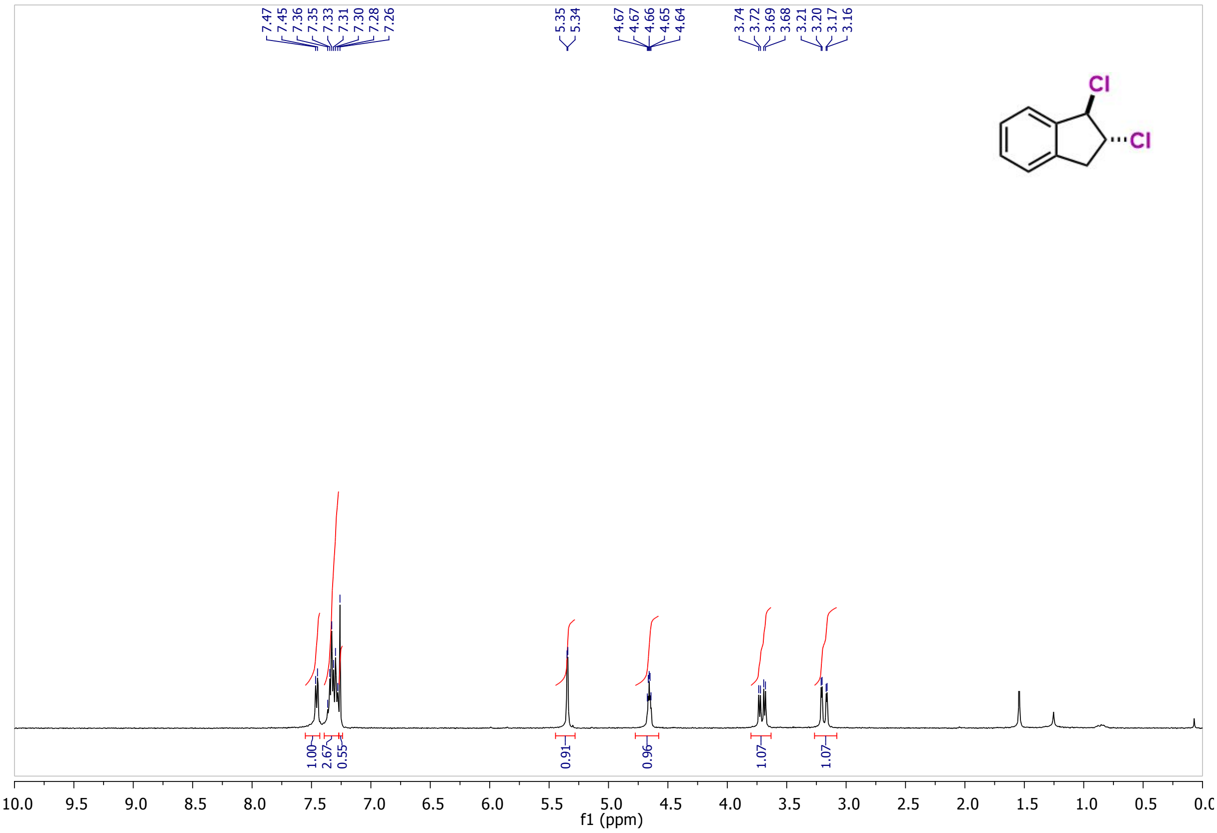

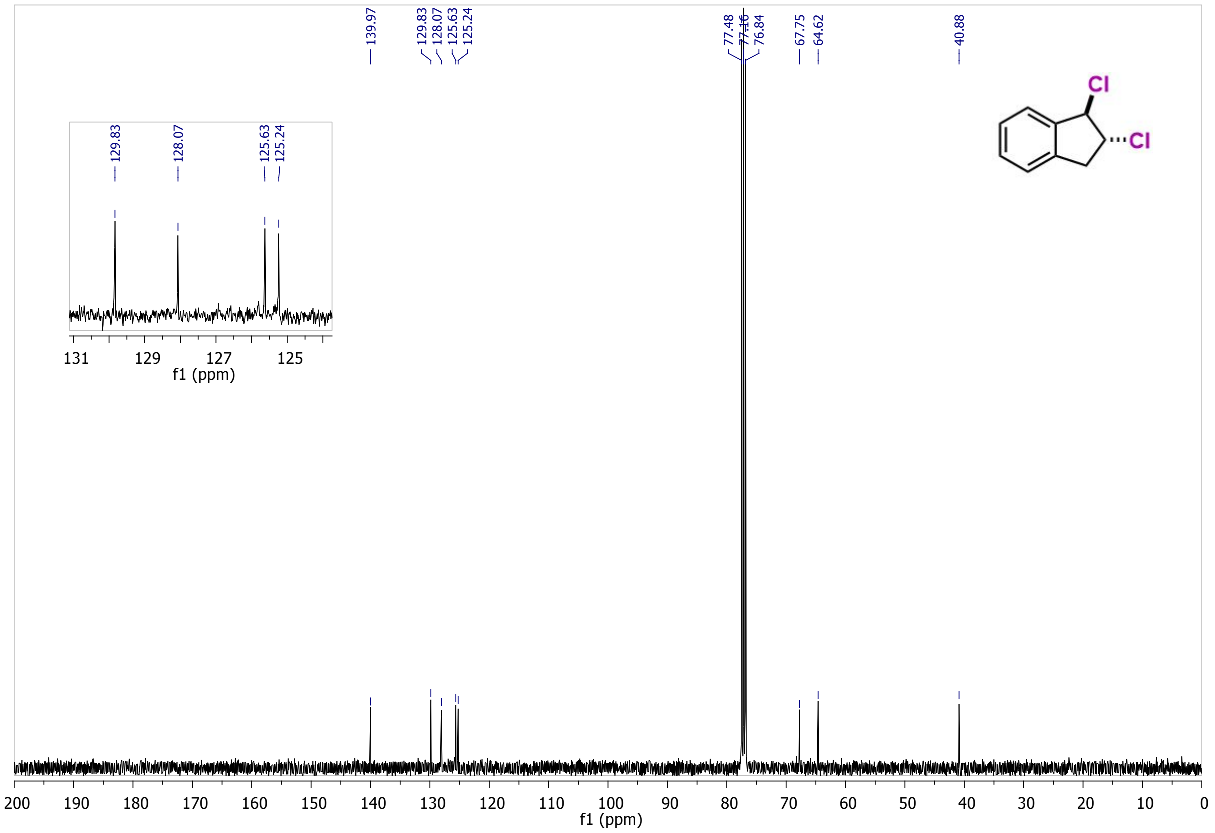

400 MHz ^{1}H-NMR (top) and 101 MHz ^{13}C-NMR (bottom) spectra of **2g** ($CDCl_3$)

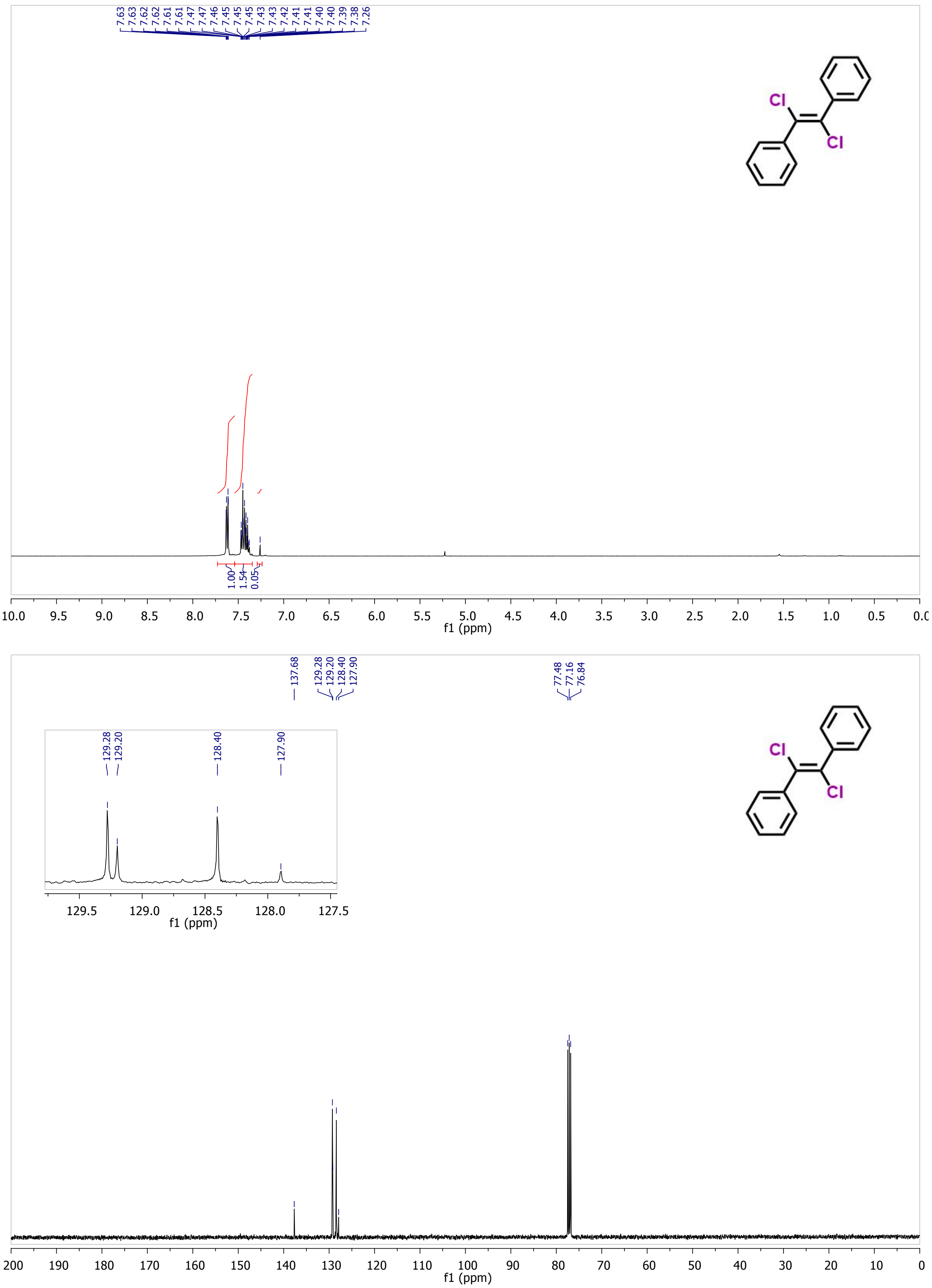

400 MHz ^{1}H-NMR (top) and 101 MHz ^{13}C-NMR (bottom) spectra of **2h** ($CDCl_3$)

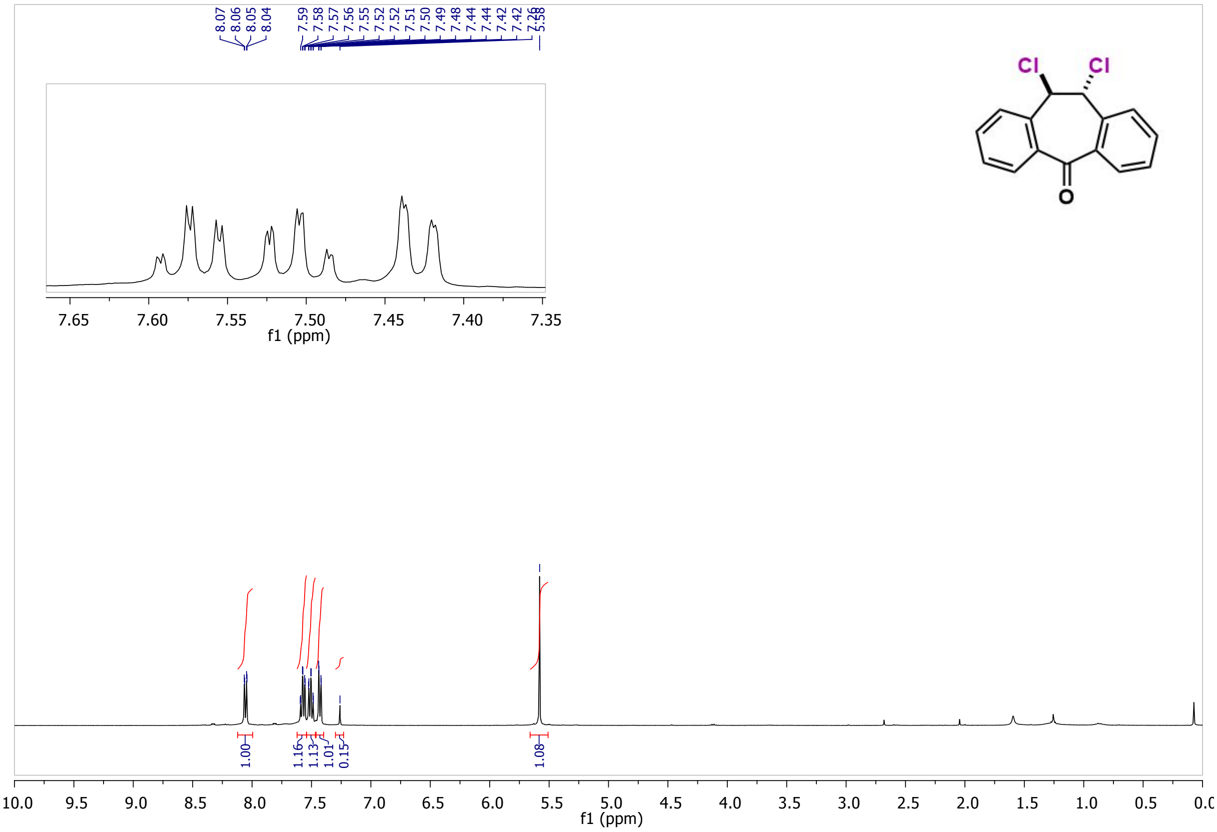

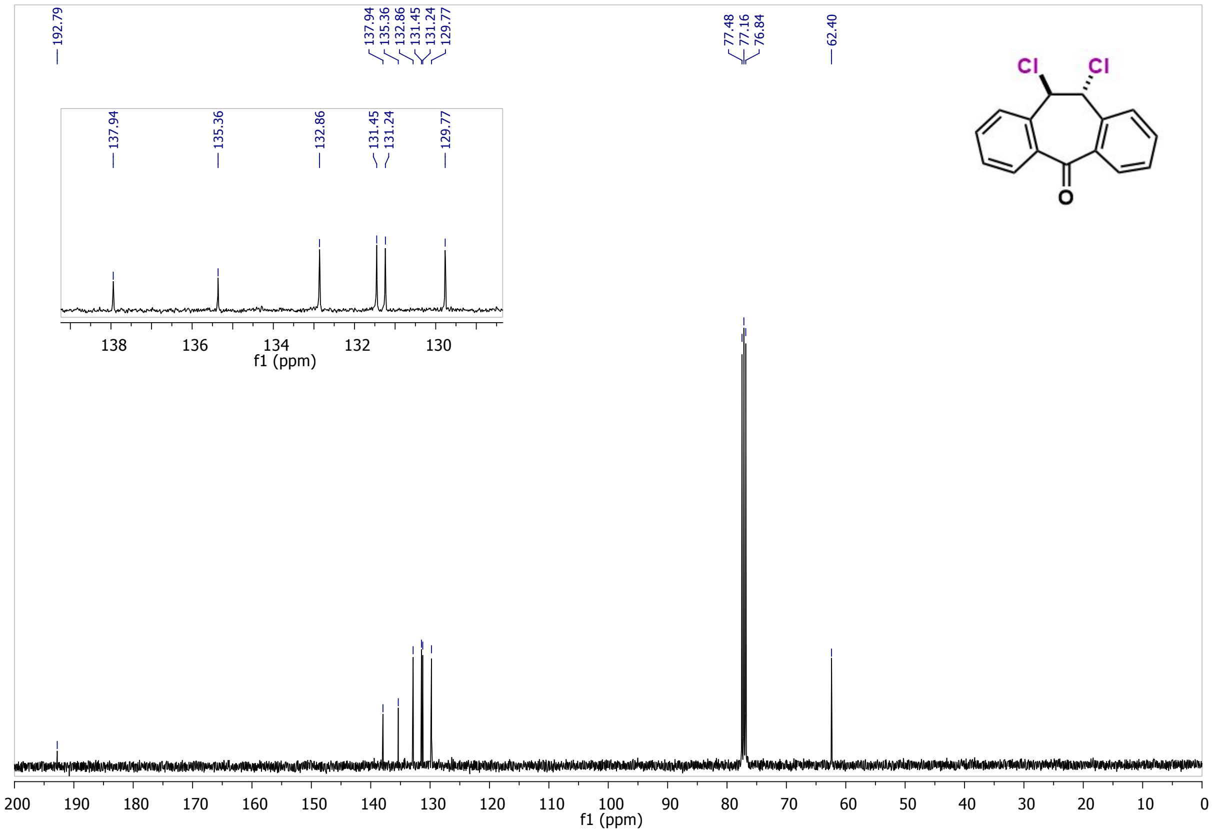

400 MHz ^{1}H-NMR (top) and 101 MHz ^{13}C-NMR (bottom) spectra of **2i** ($CDCl_3$)

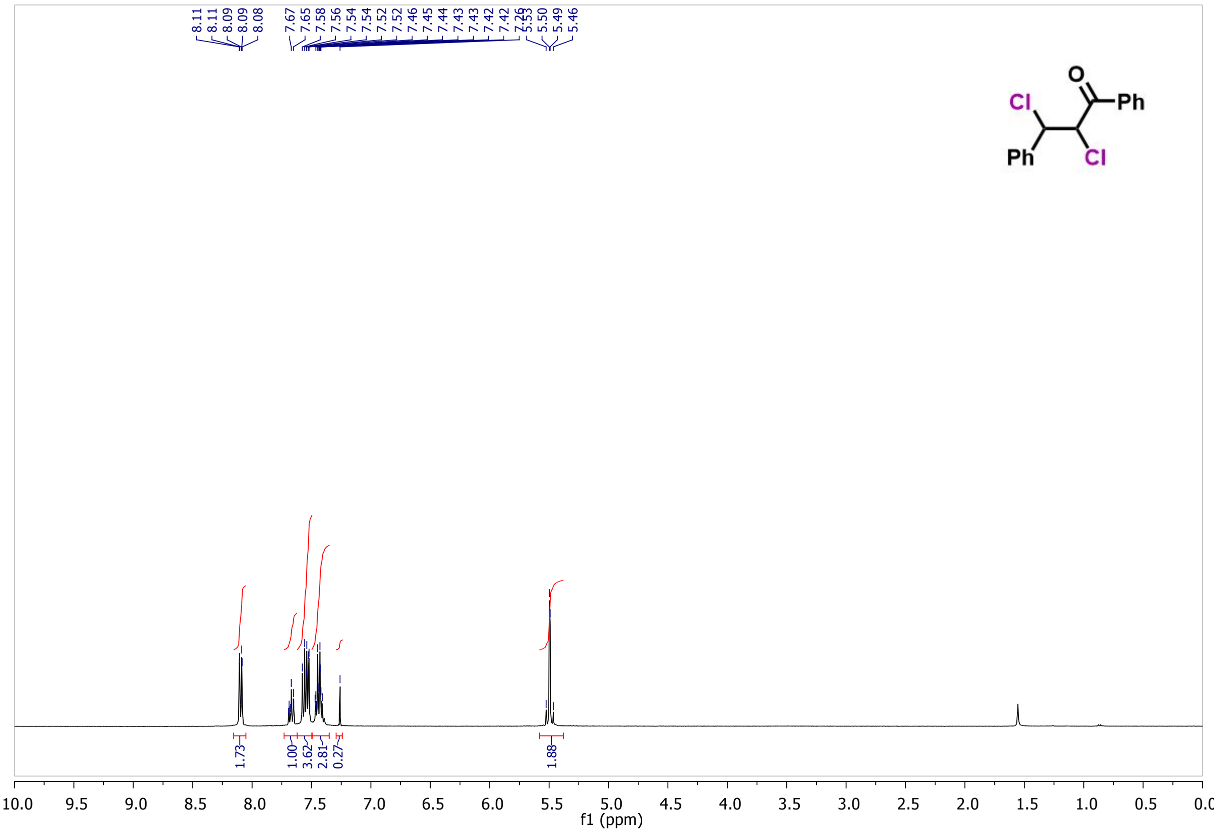

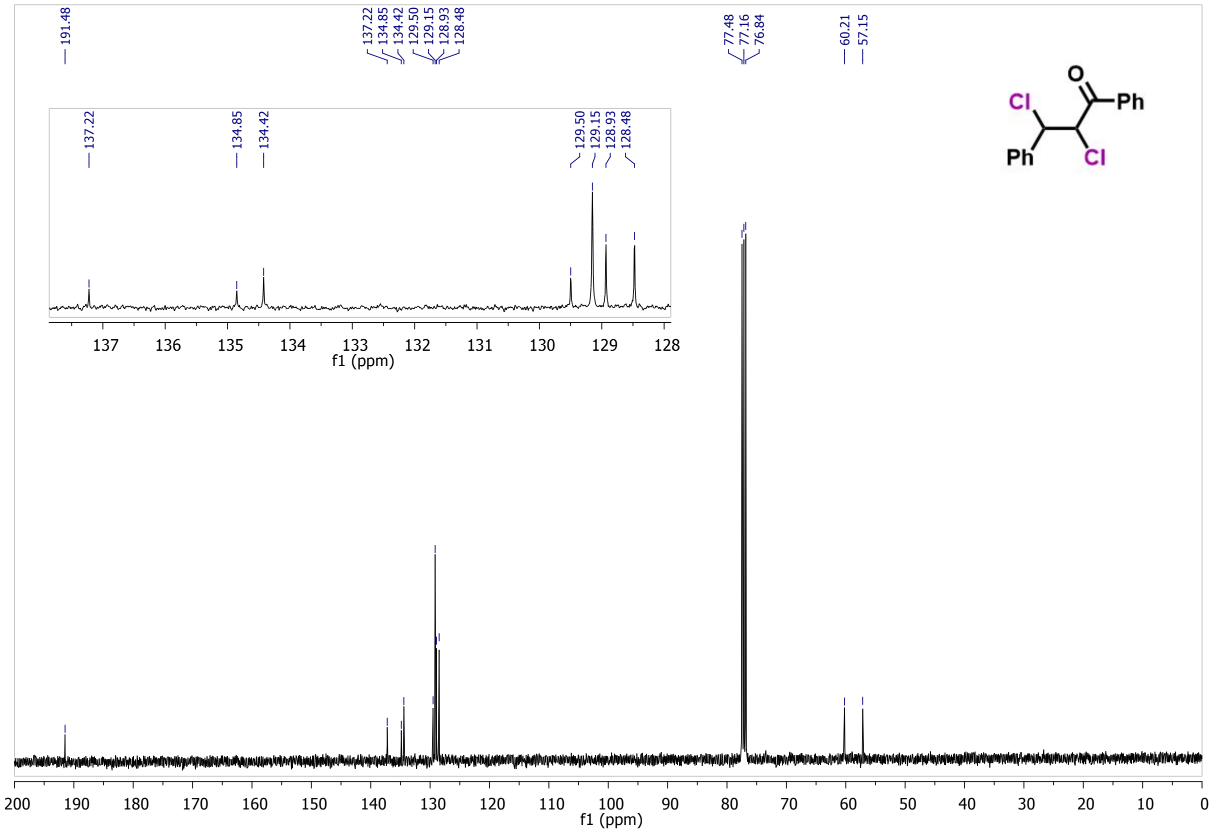

400 MHz ^{1}H-NMR (top) and 101 MHz ^{13}C-NMR (bottom) spectra of **2j** ($CDCl_3$)

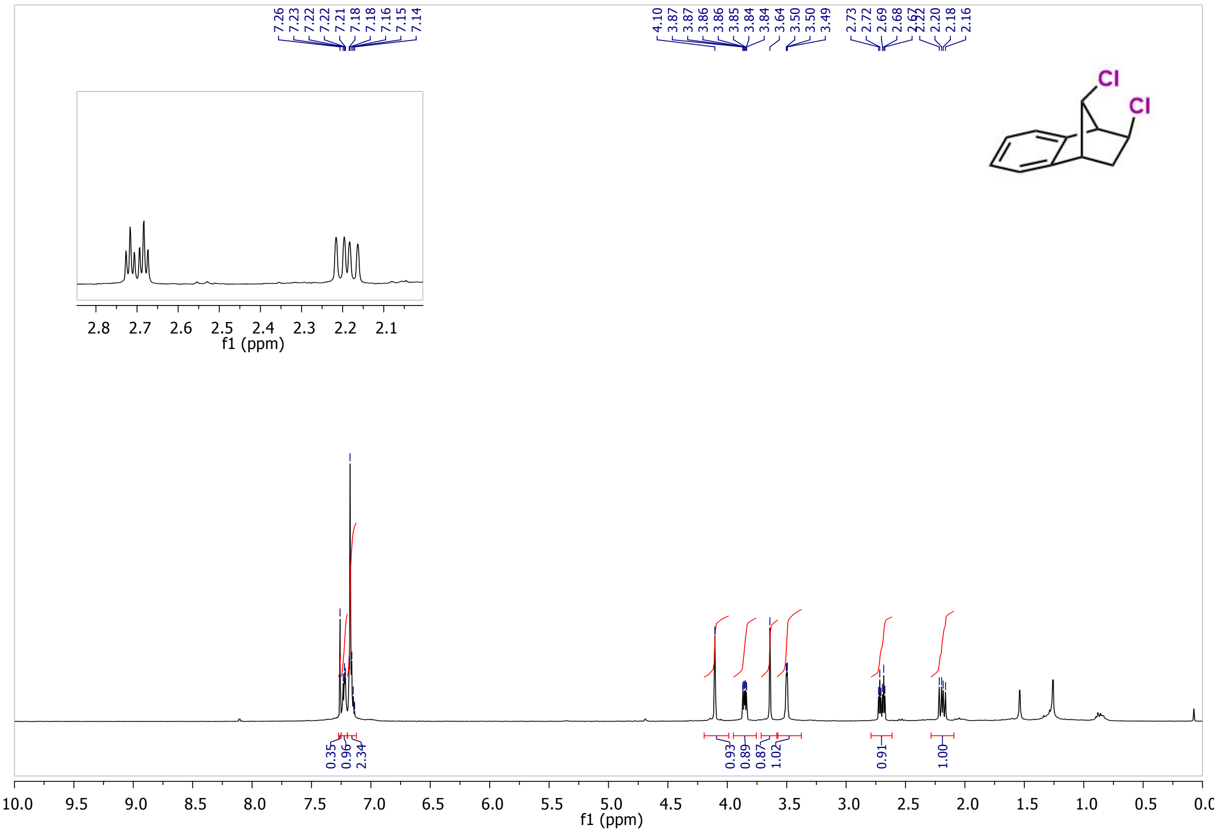

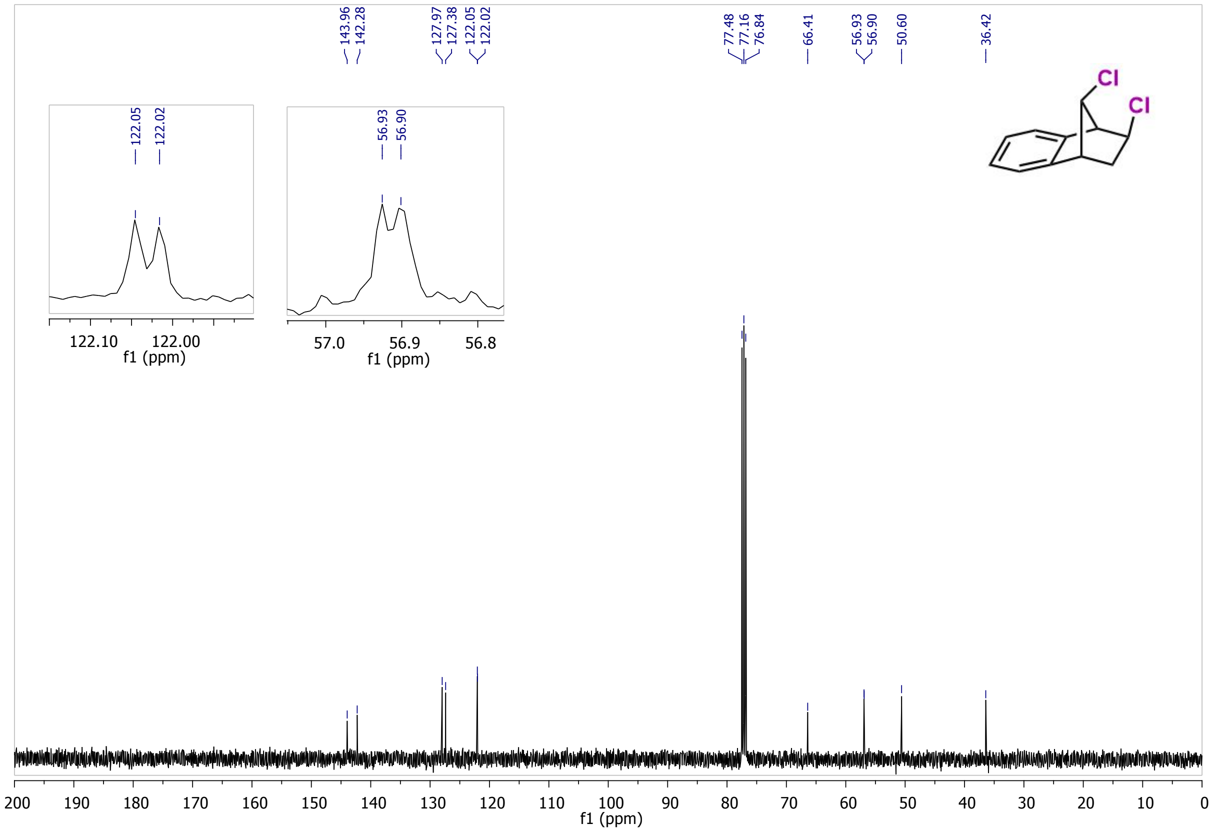

400 MHz ^{1}H-NMR (top) and 101 MHz ^{13}C-NMR (bottom) spectra of **2k** ($CDCl_3$)

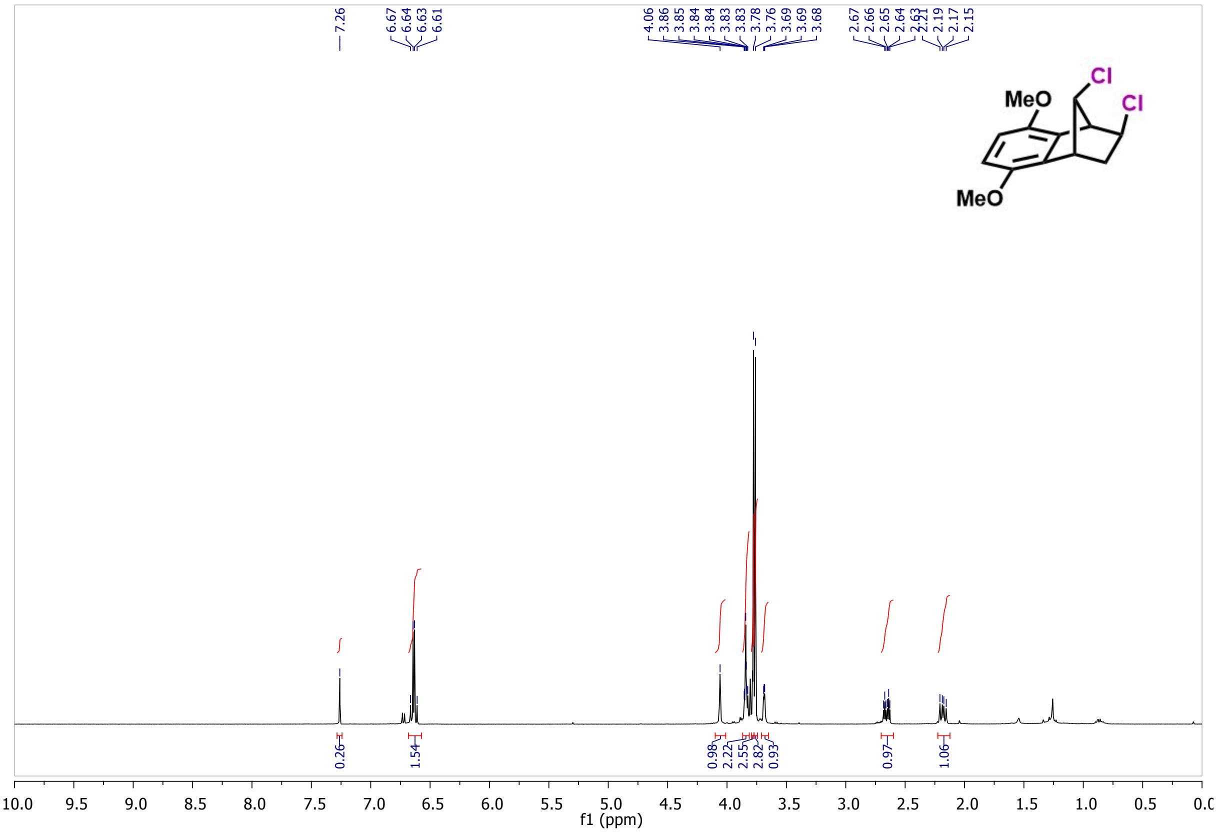

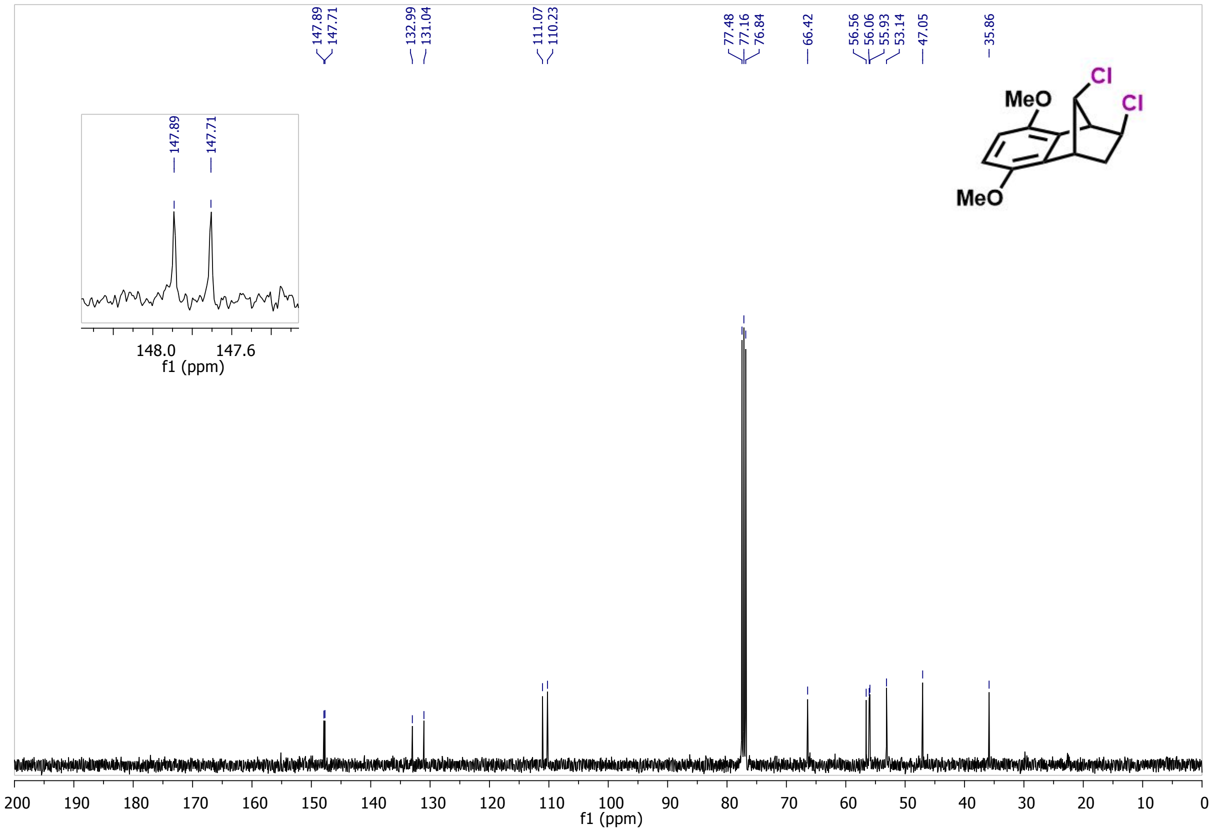

400 MHz ^{1}H-NMR (top) and 101 MHz ^{13}C-NMR (bottom) spectra of **2l** ($CDCl_3$)

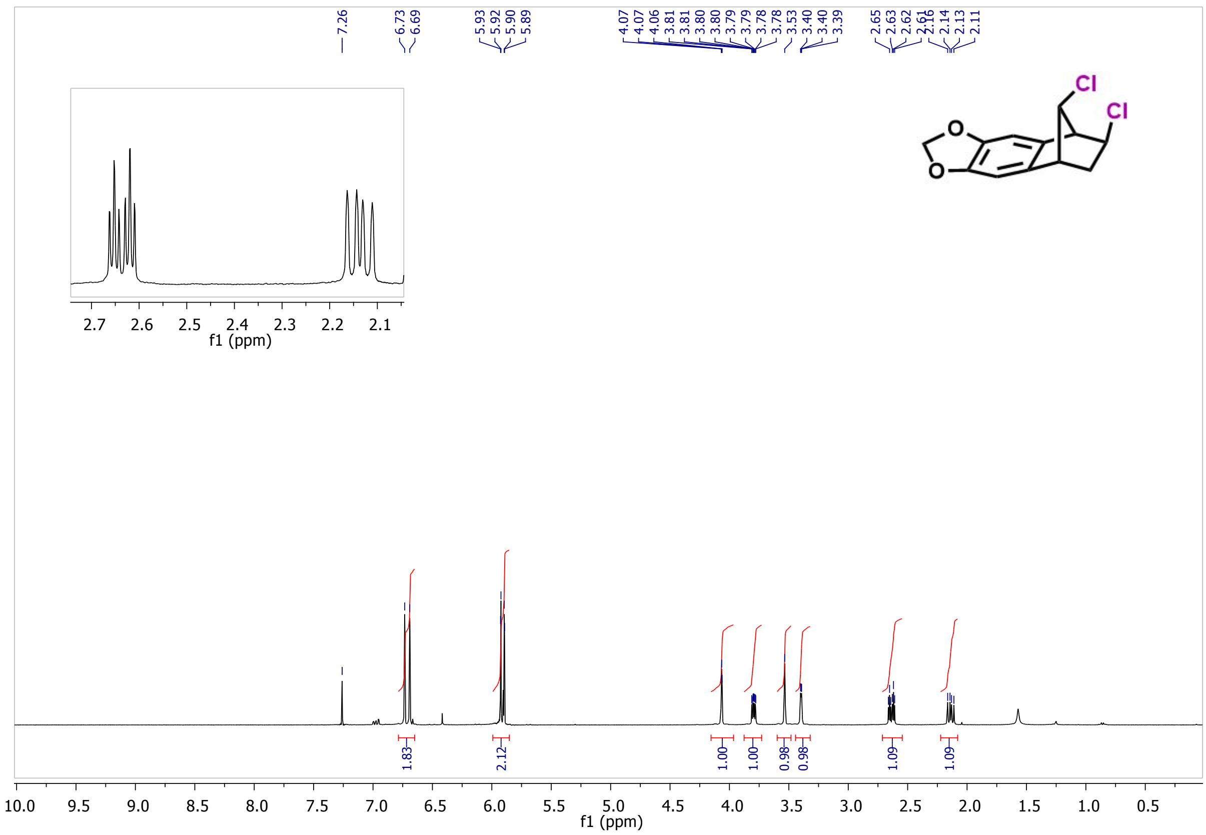

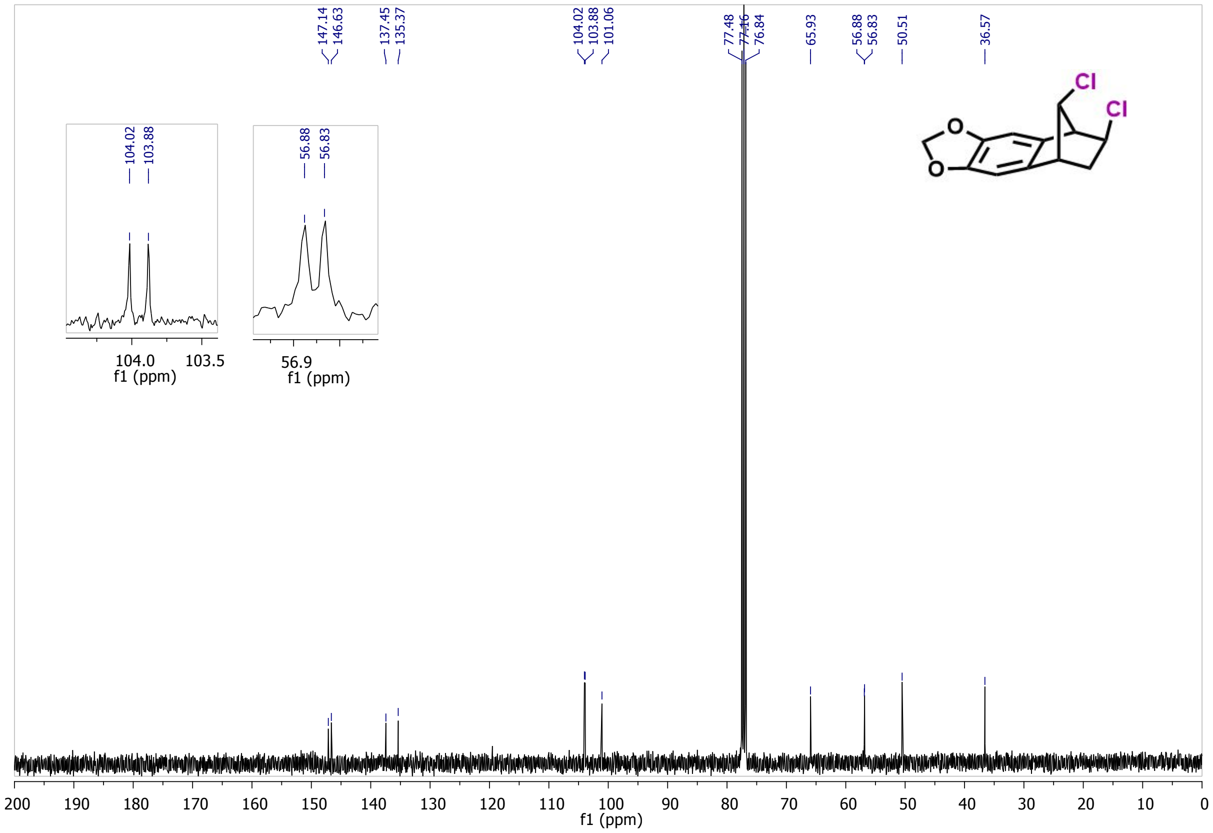

400 MHz ^{1}H-NMR (top) and 101 MHz ^{13}C-NMR (bottom) spectra of **2m** ($CDCl_3$)

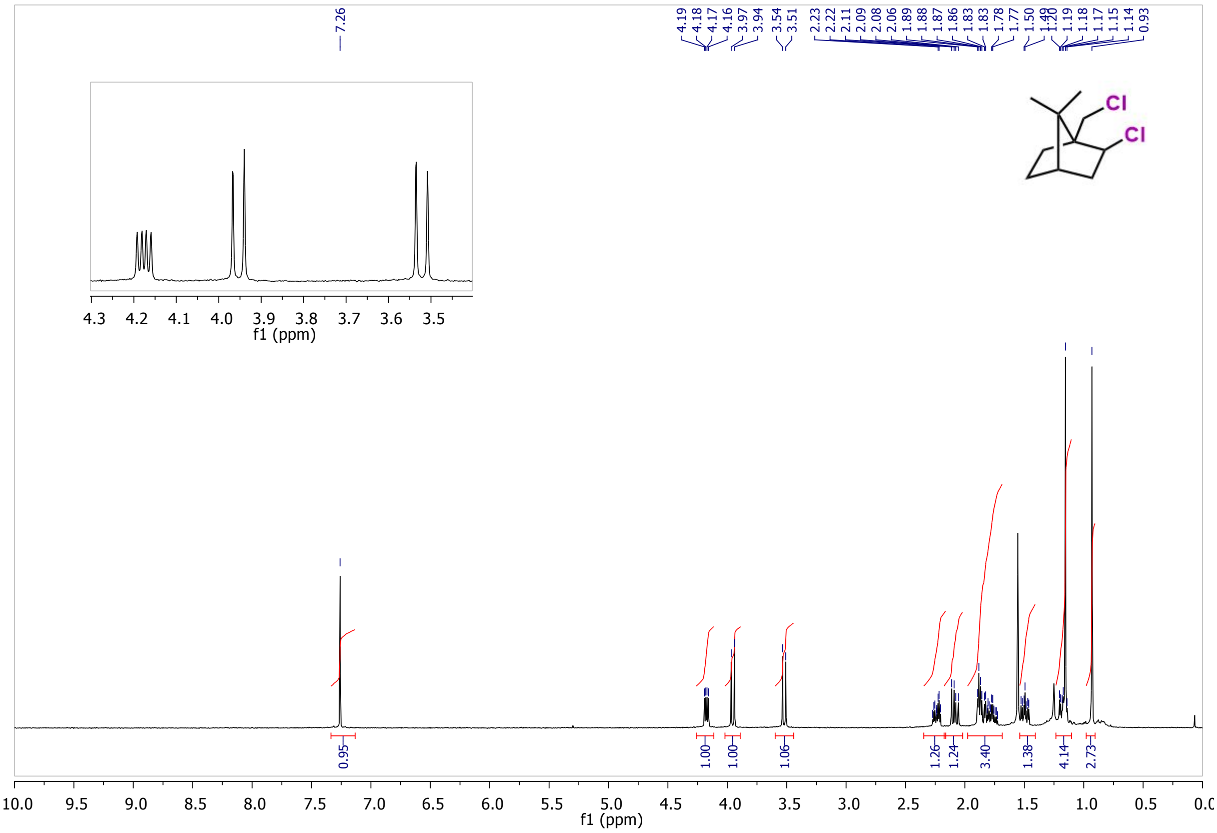

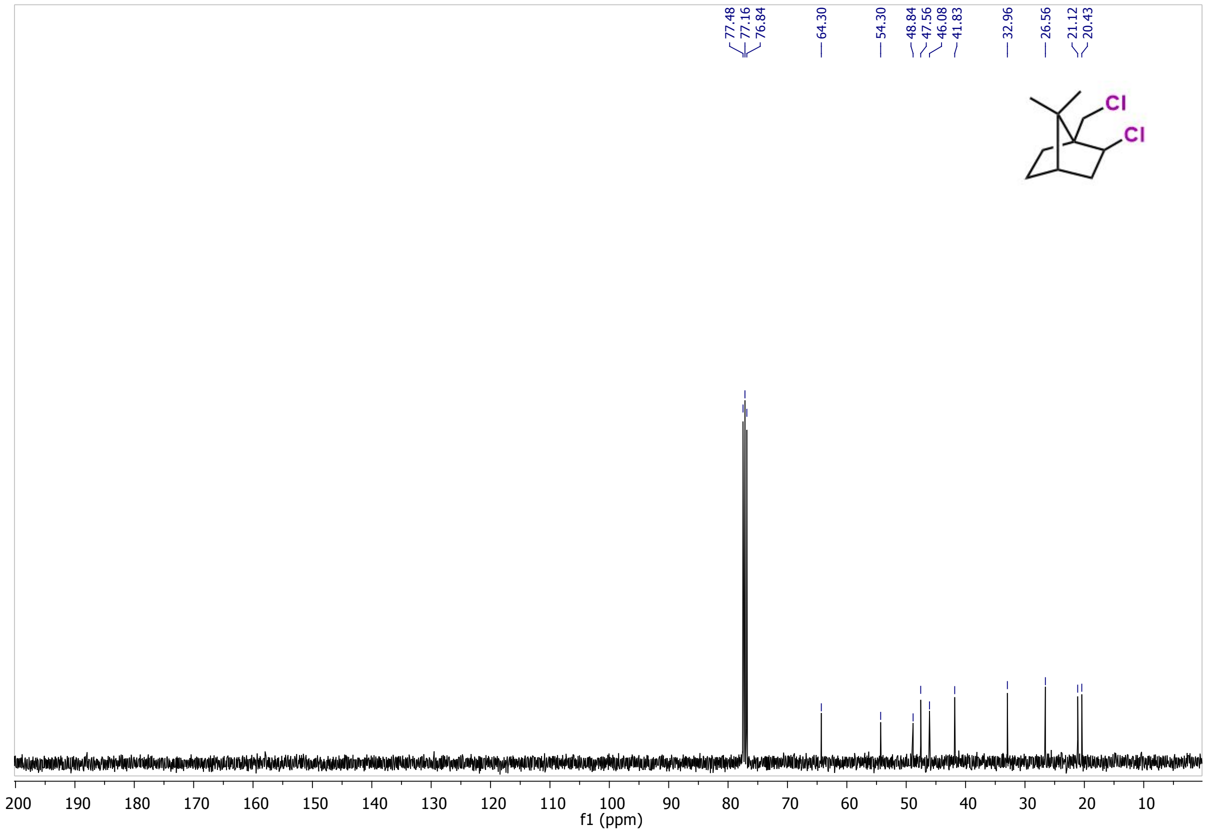

400 MHz ^{1}H-NMR (top) and 101 MHz ^{13}C-NMR (bottom) spectra of **2n** ($CDCl_3$)

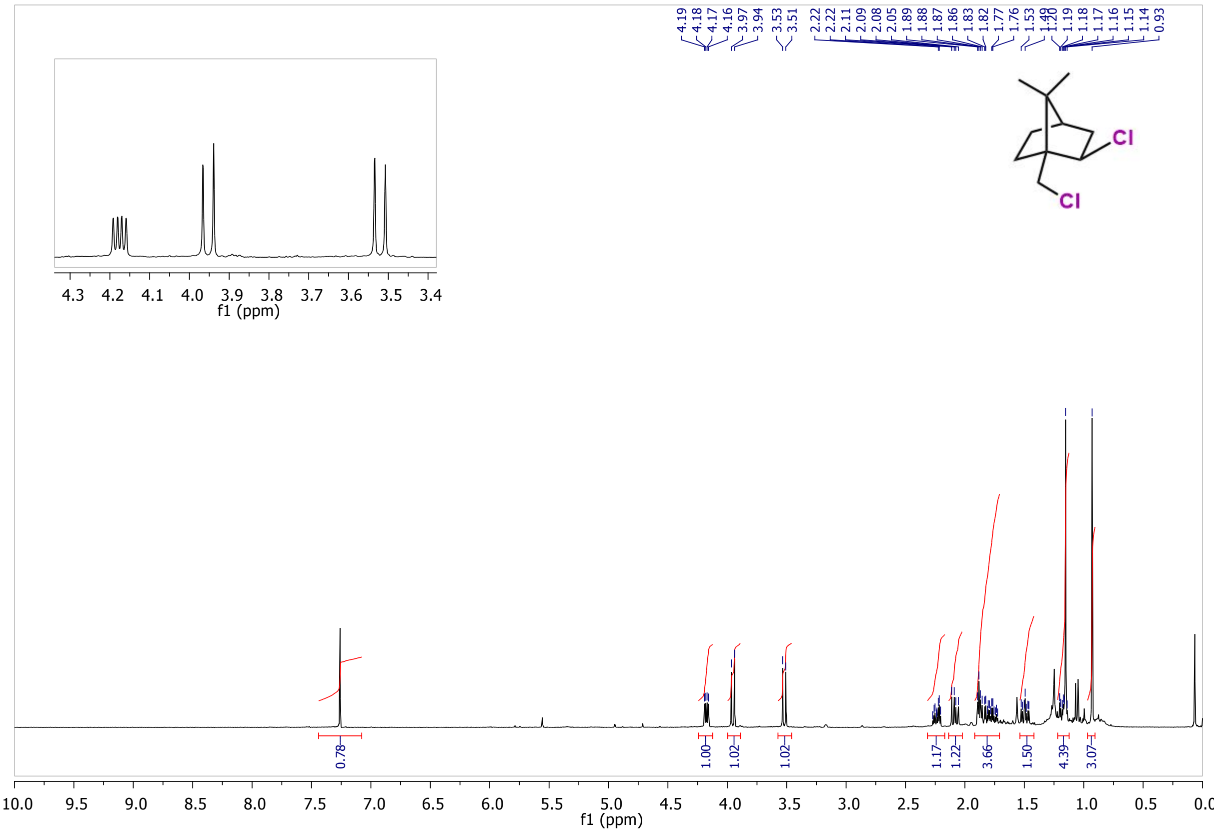

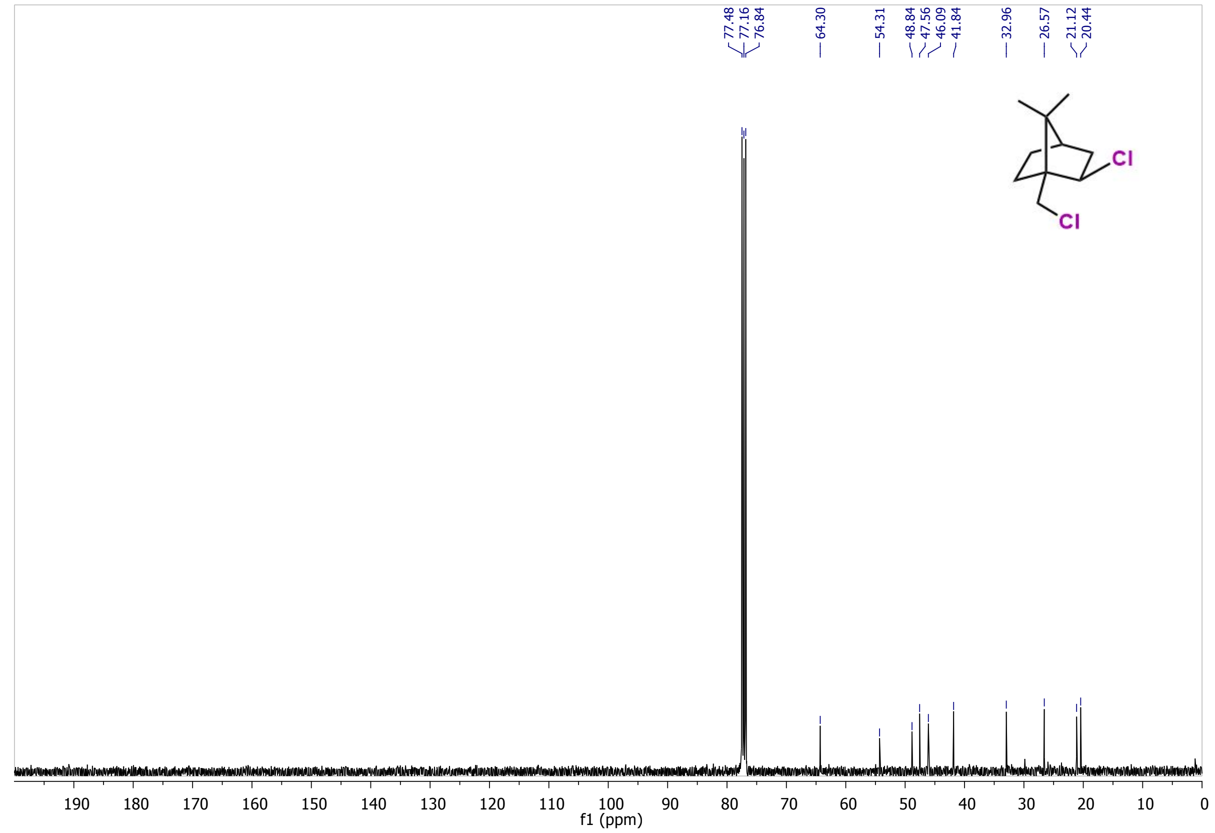

400 MHz ^{1}H-NMR (top) and 101 MHz ^{13}C-NMR (bottom) spectra of **2o** ($CDCl_3$)

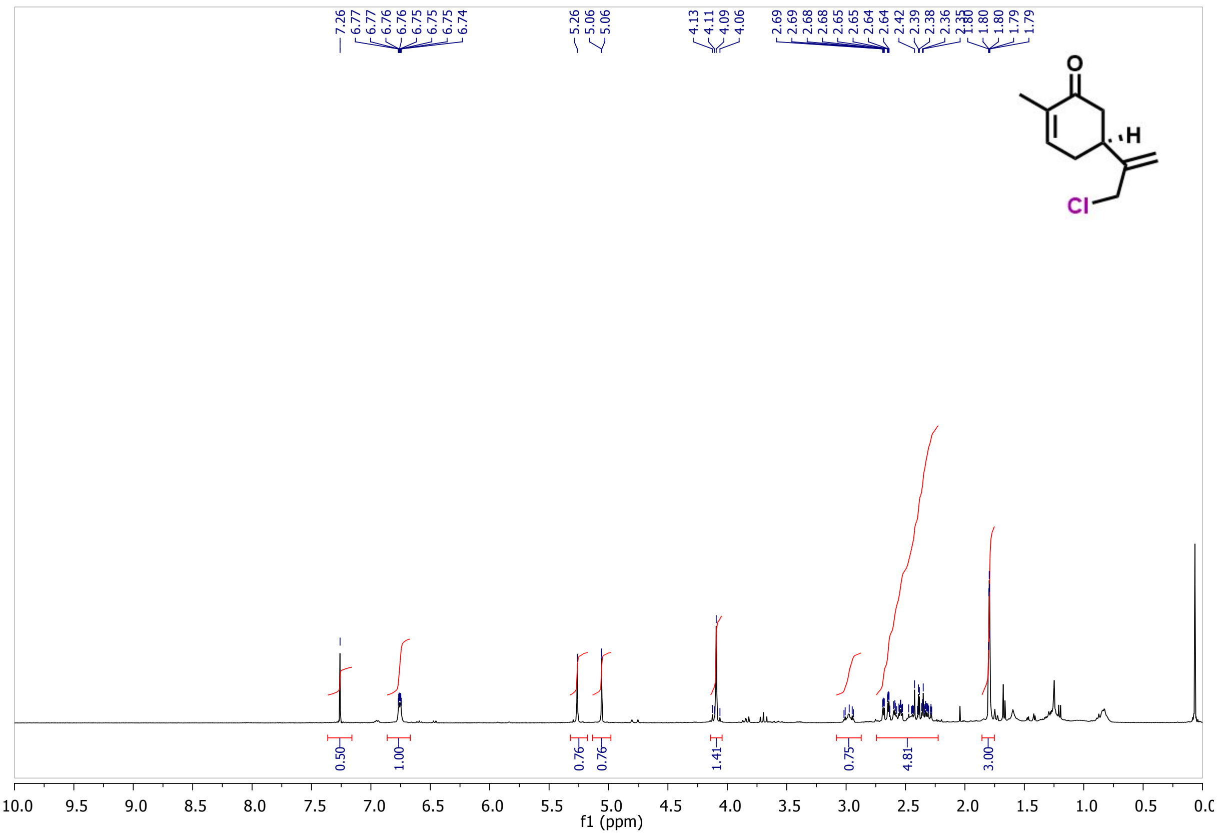

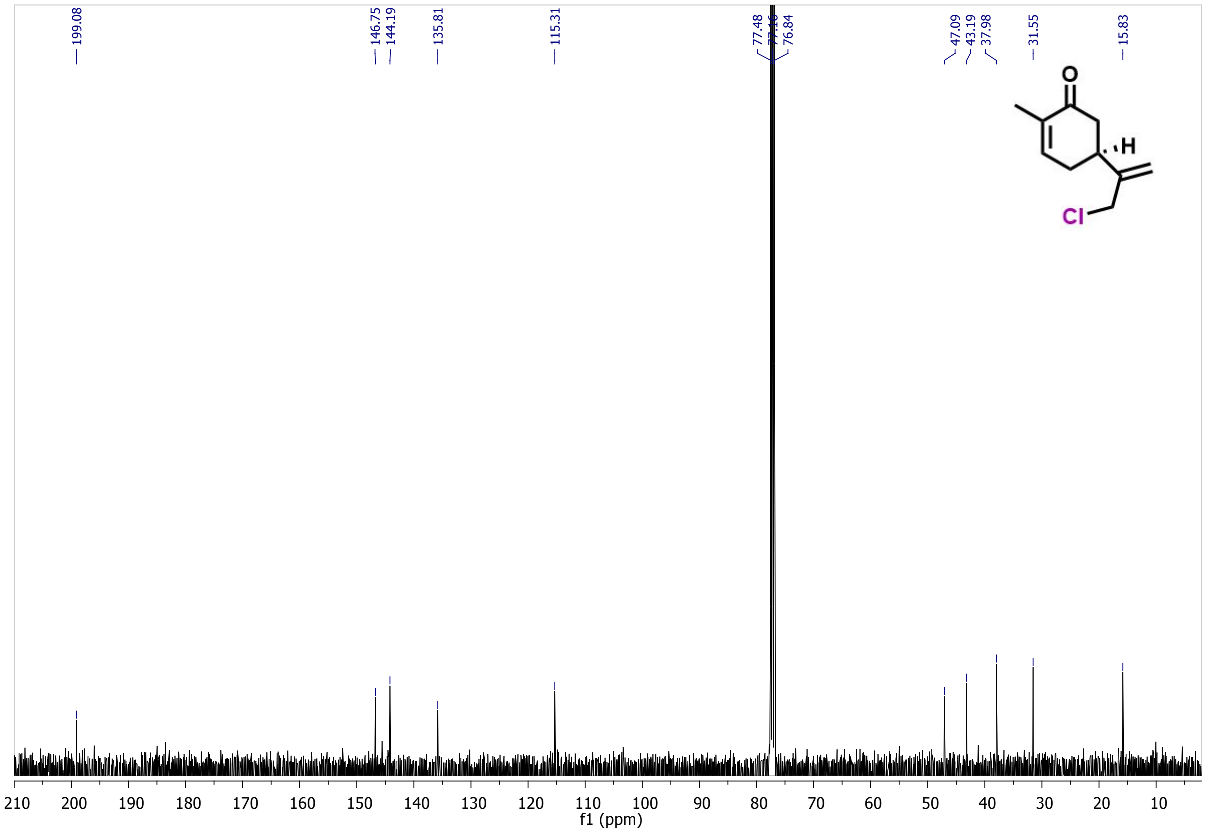

400 MHz ^{1}H-NMR (top) and 101 MHz ^{13}C-NMR (bottom) spectra of **2p** ($CDCl_3$)

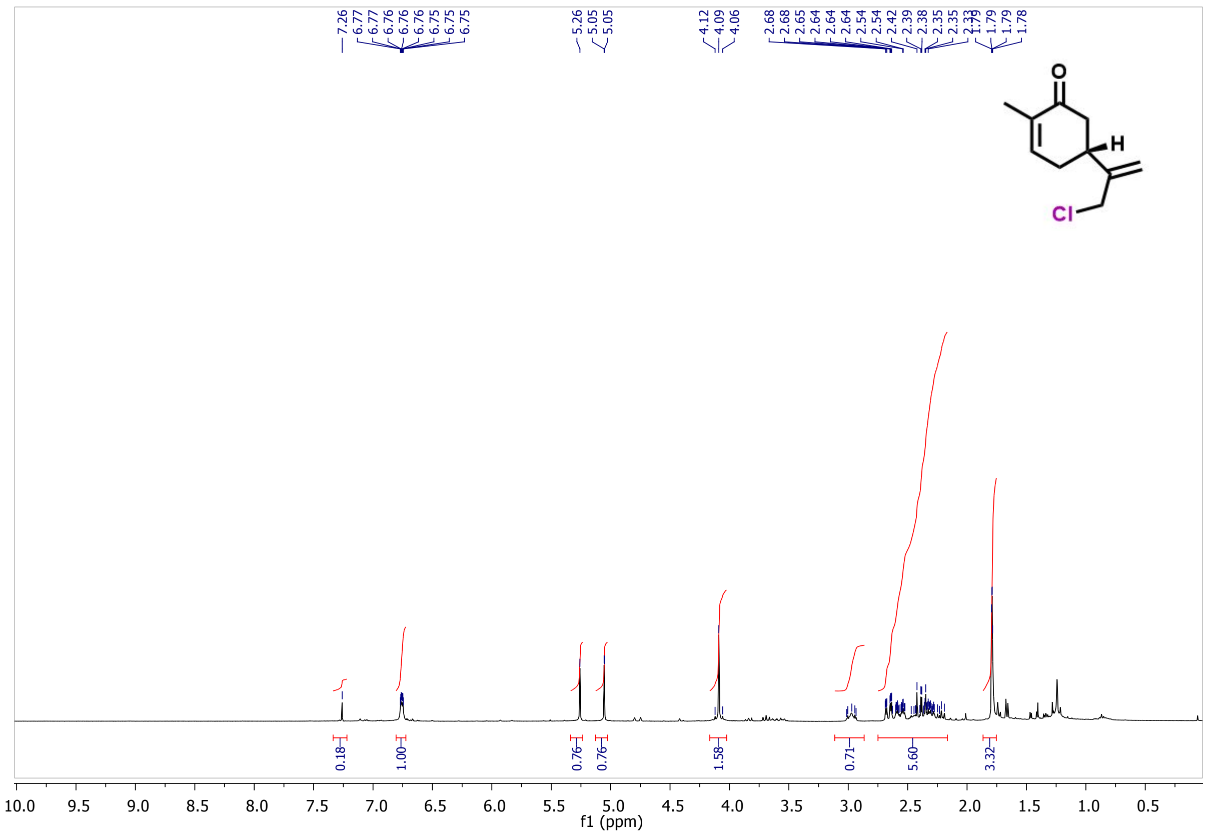

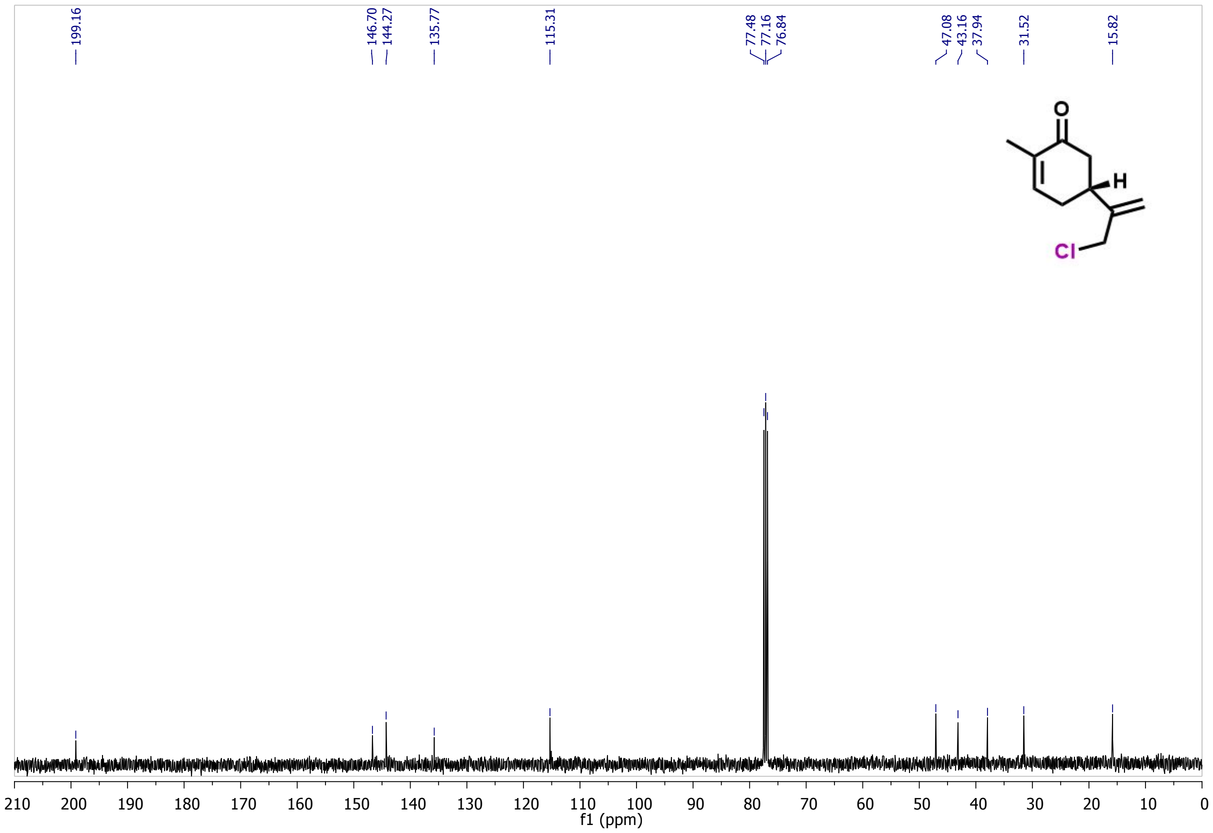

400 MHz ^{1}H-NMR (top) and 101 MHz ^{13}C-NMR (bottom) spectra of **2q** ($CDCl_3$)

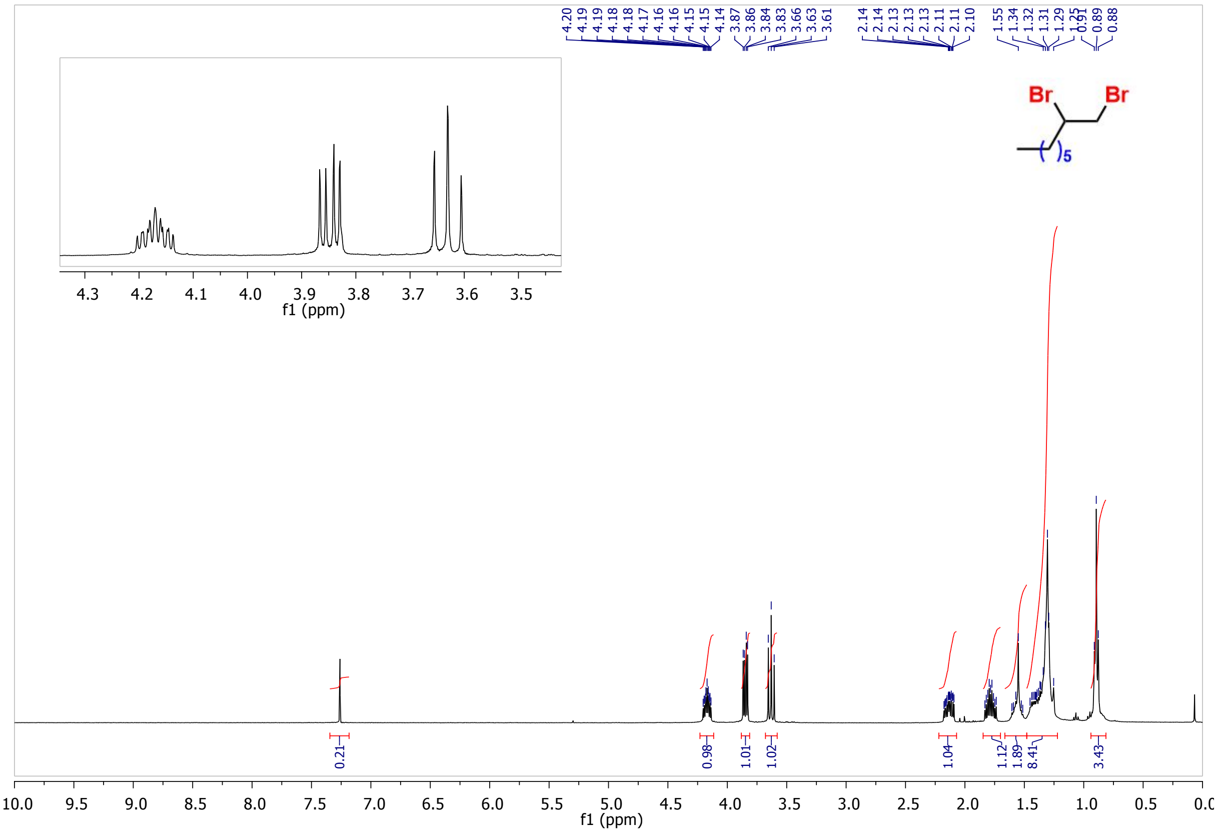

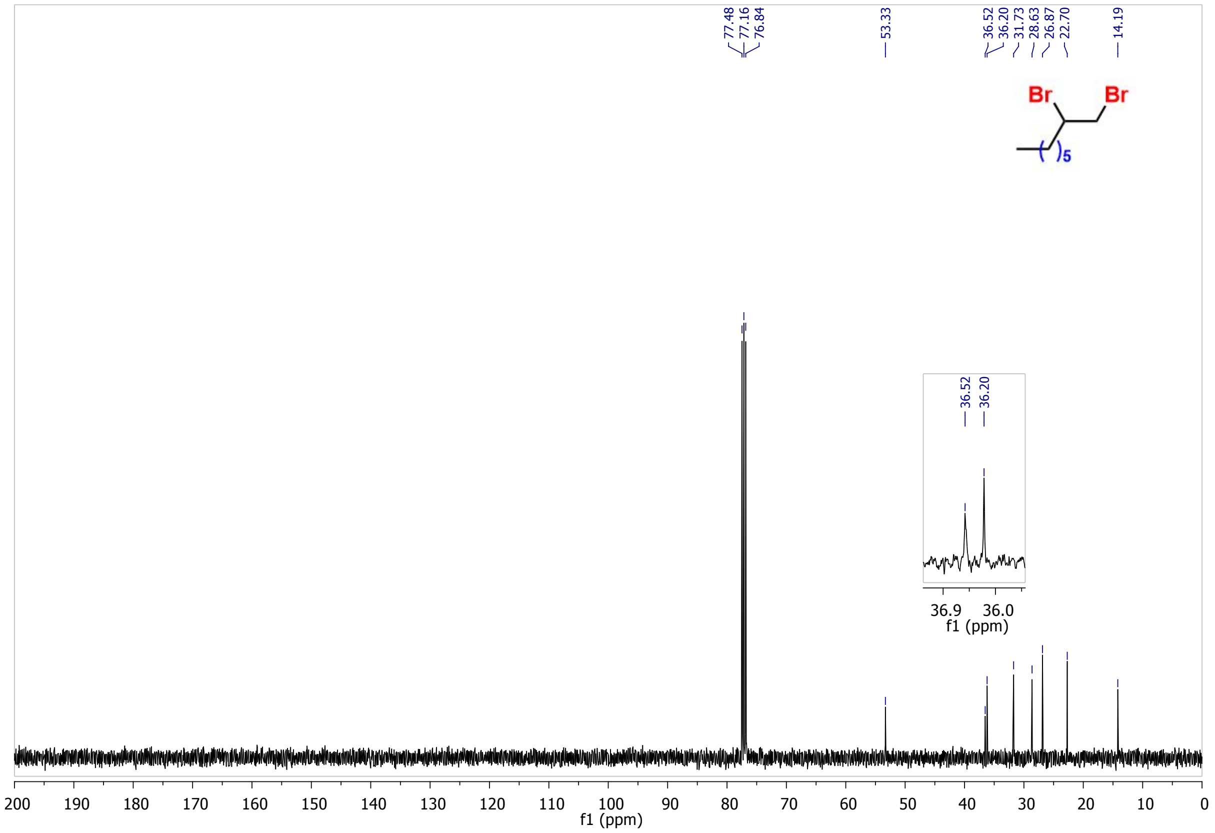

400 MHz ^{1}H-NMR (top) and 101 MHz ^{13}C-NMR (bottom) spectra of **3a** ($CDCl_3$)

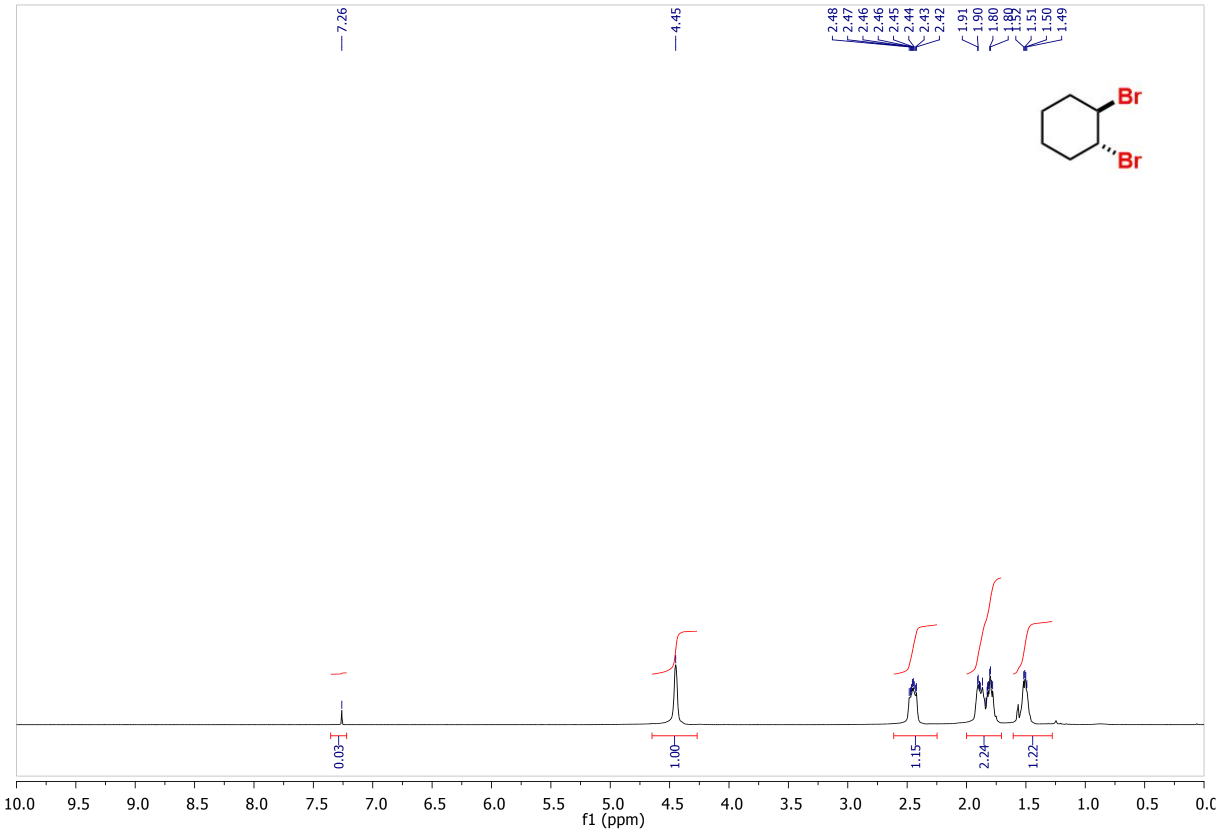

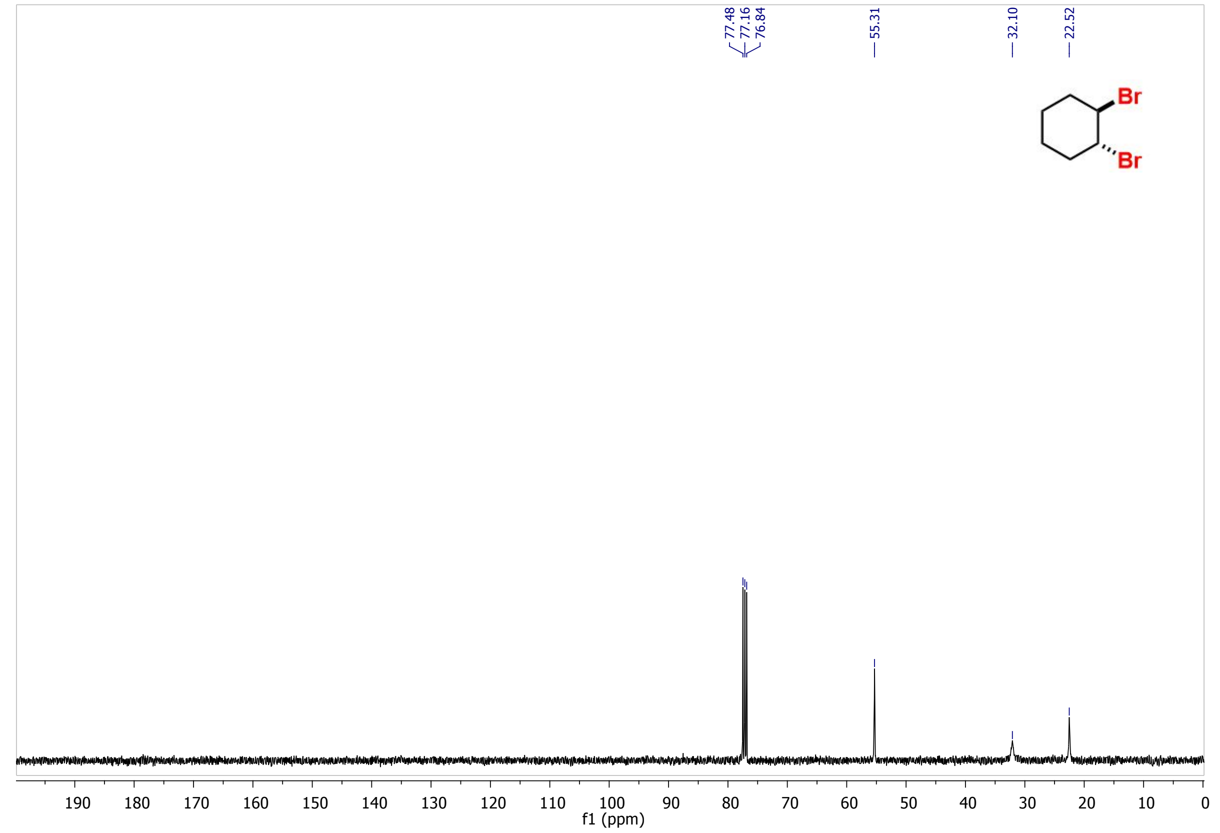

400 MHz ^{1}H-NMR (top) and 101 MHz ^{13}C-NMR (bottom) spectra of **3b** ($CDCl_3$)

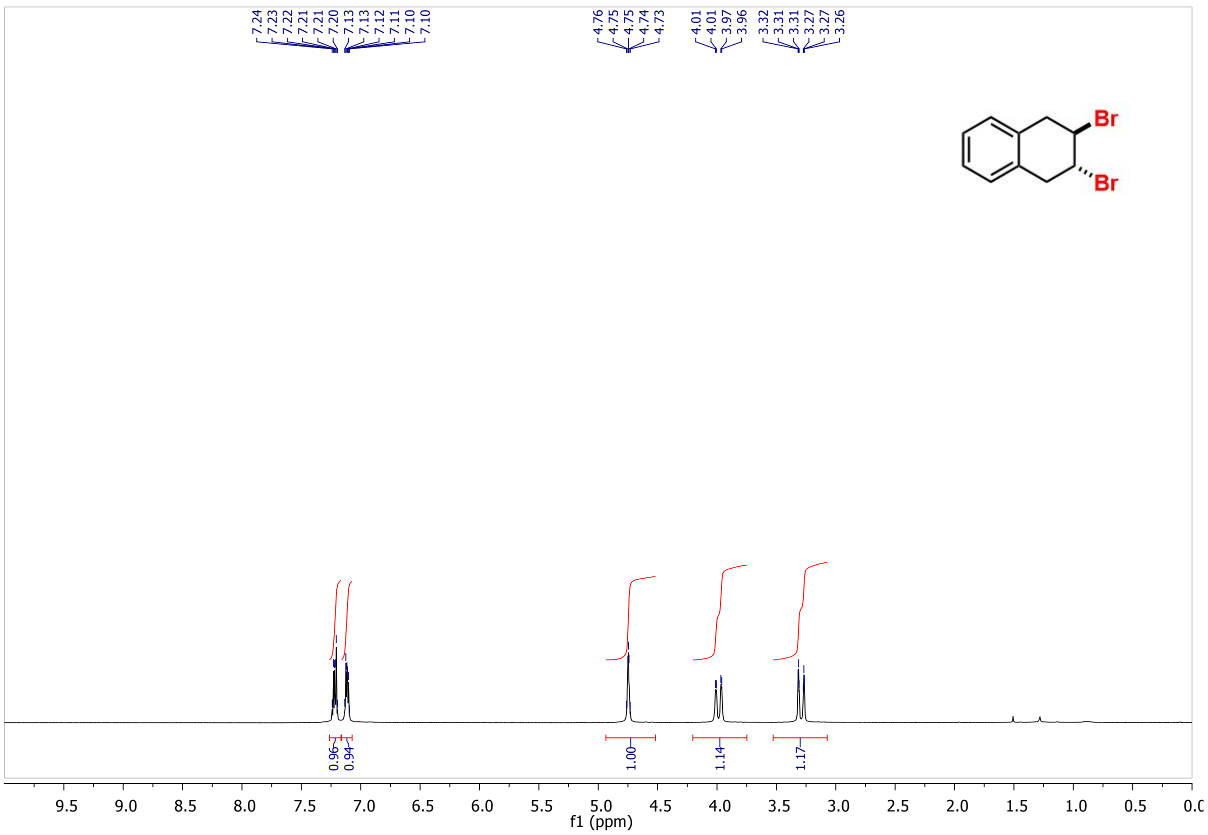

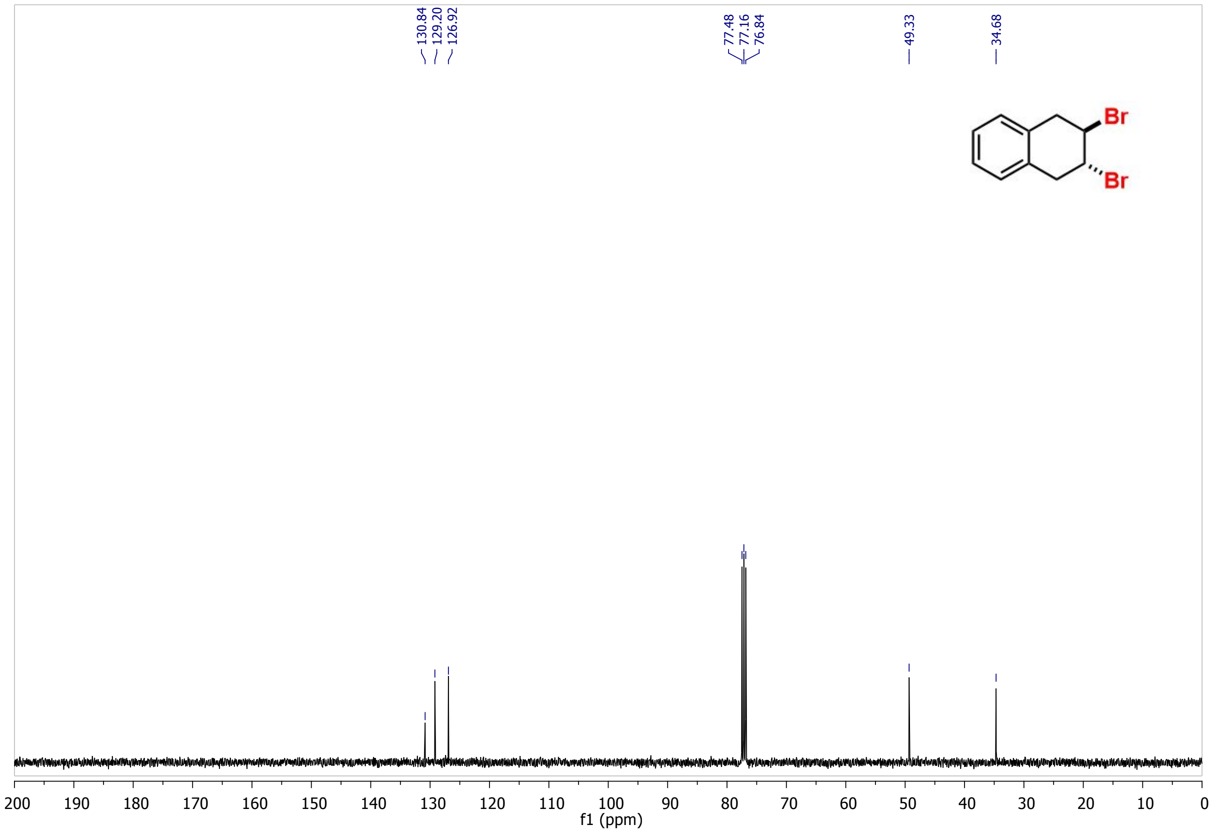

400 MHz ^{1}H-NMR (top) and 101 MHz ^{13}C-NMR (bottom) spectra of **3c** ($CDCl_3$)

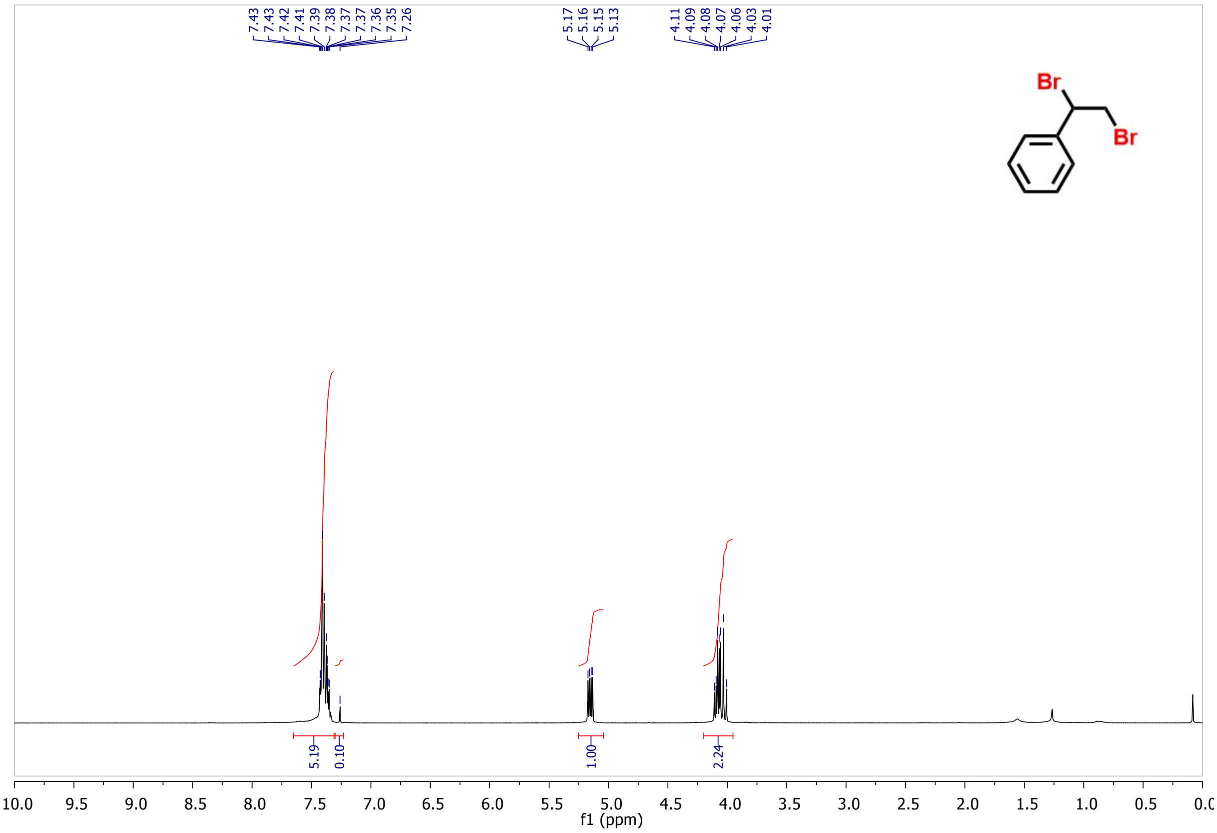

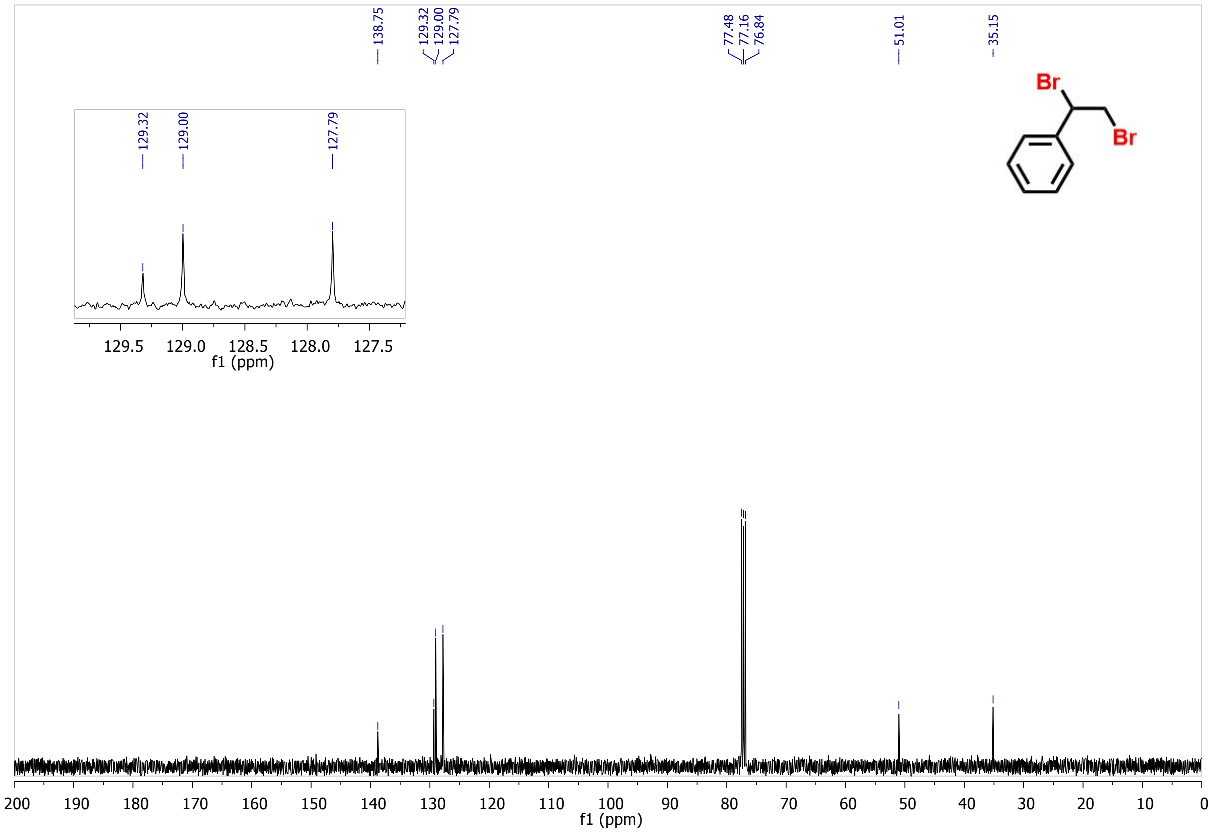

400 MHz ^{1}H-NMR (top) and 101 MHz ^{13}C-NMR (bottom) spectra of **3d** ($CDCl_3$)

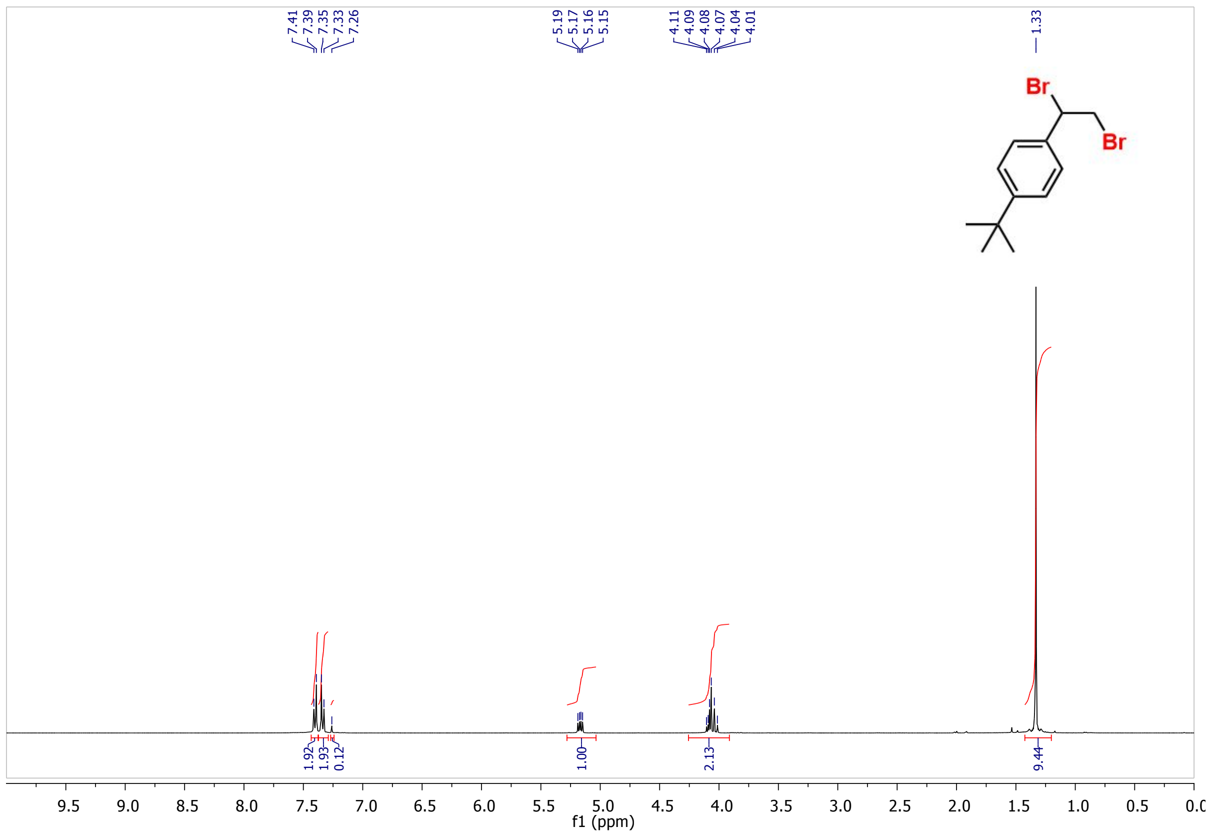

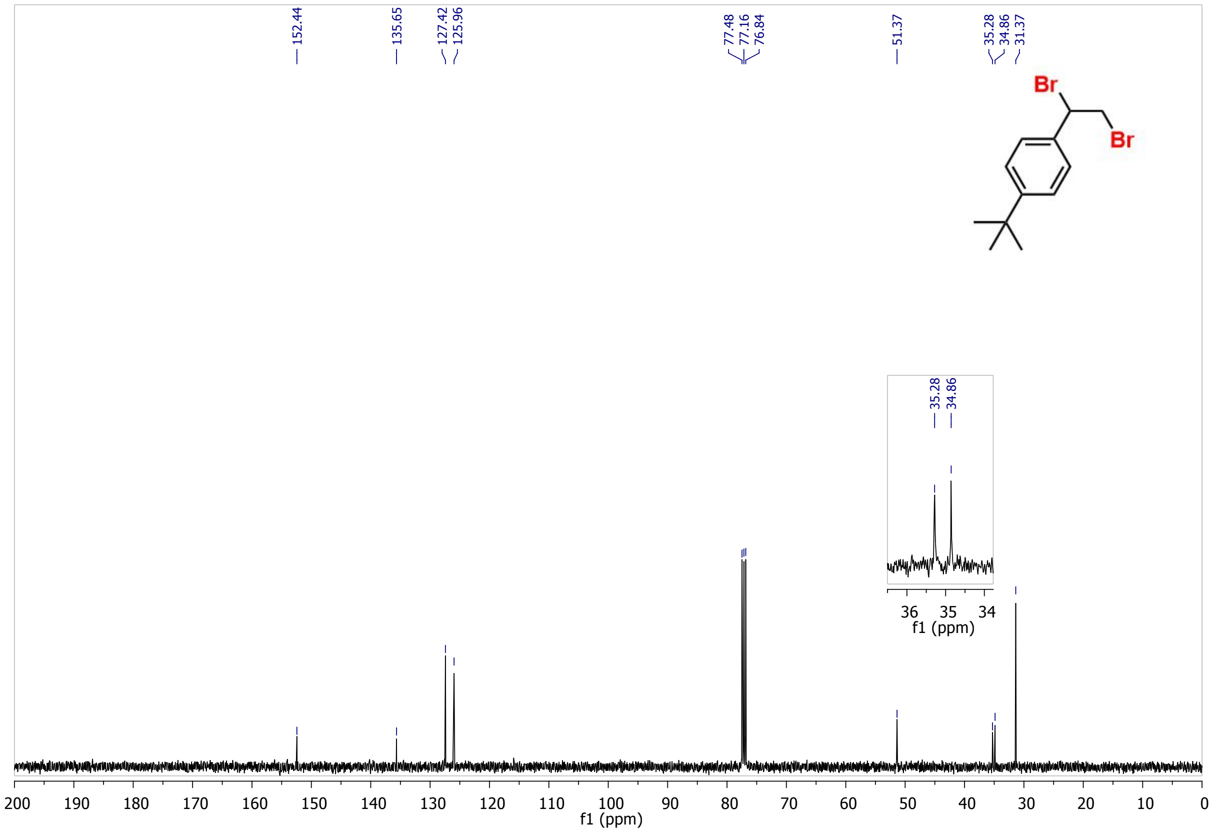

400 MHz ^{1}H-NMR (top) and 101 MHz ^{13}C-NMR (bottom) spectra of **3e** ($CDCl_3$)

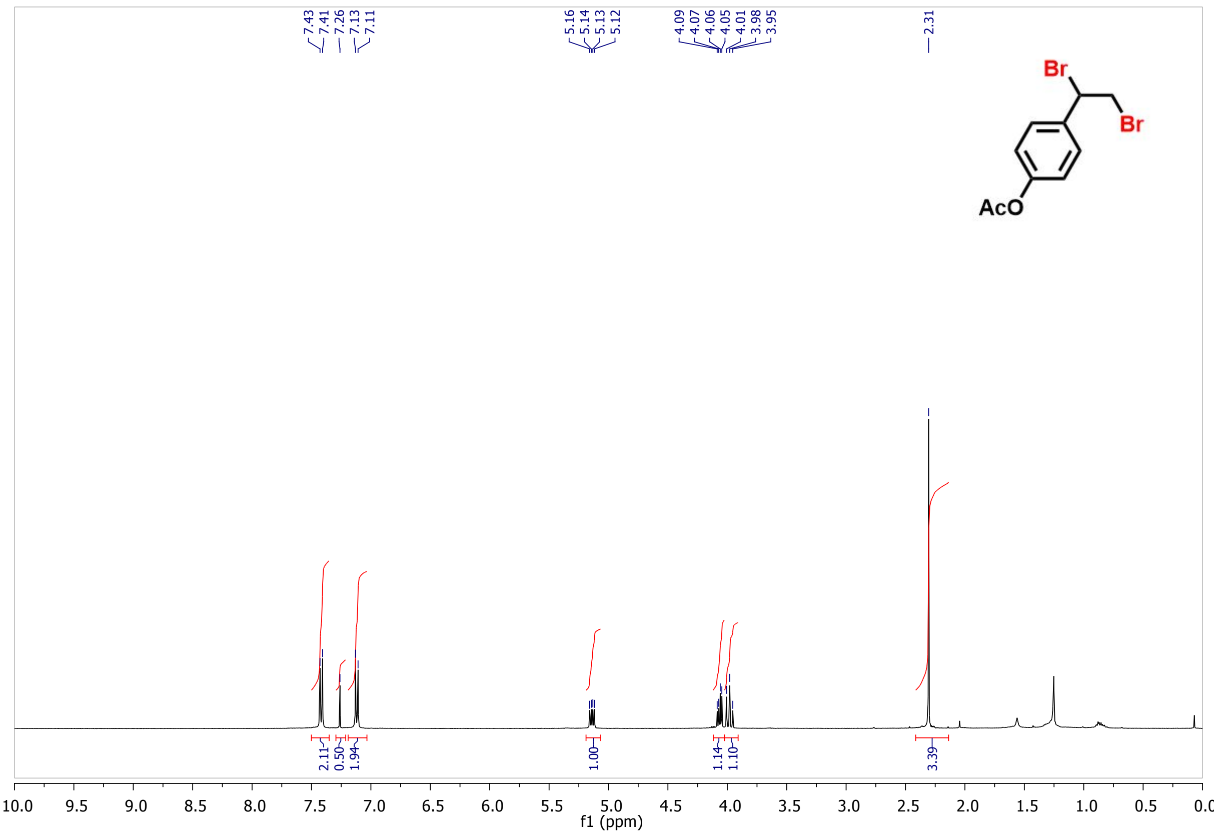

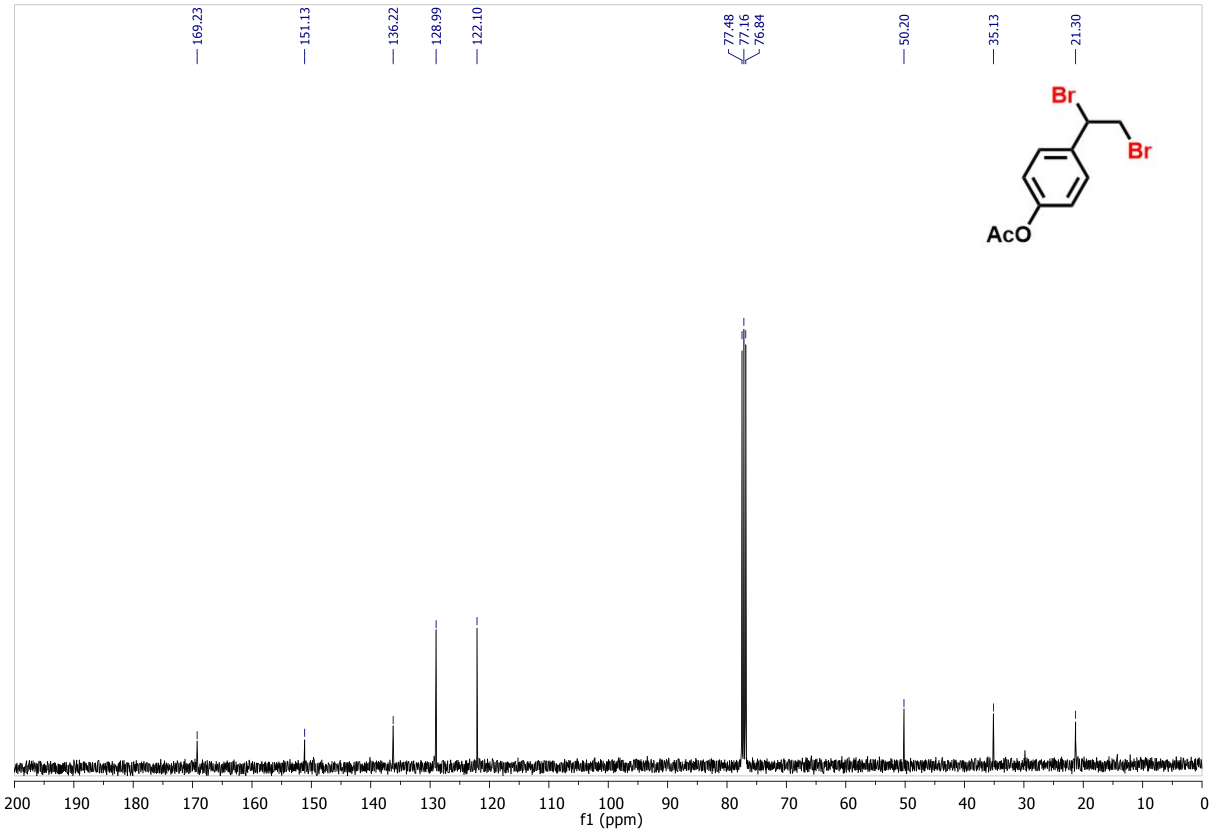

400 MHz ^{1}H-NMR (top) and 101 MHz ^{13}C-NMR (bottom) spectra of **3f** ($CDCl_3$)

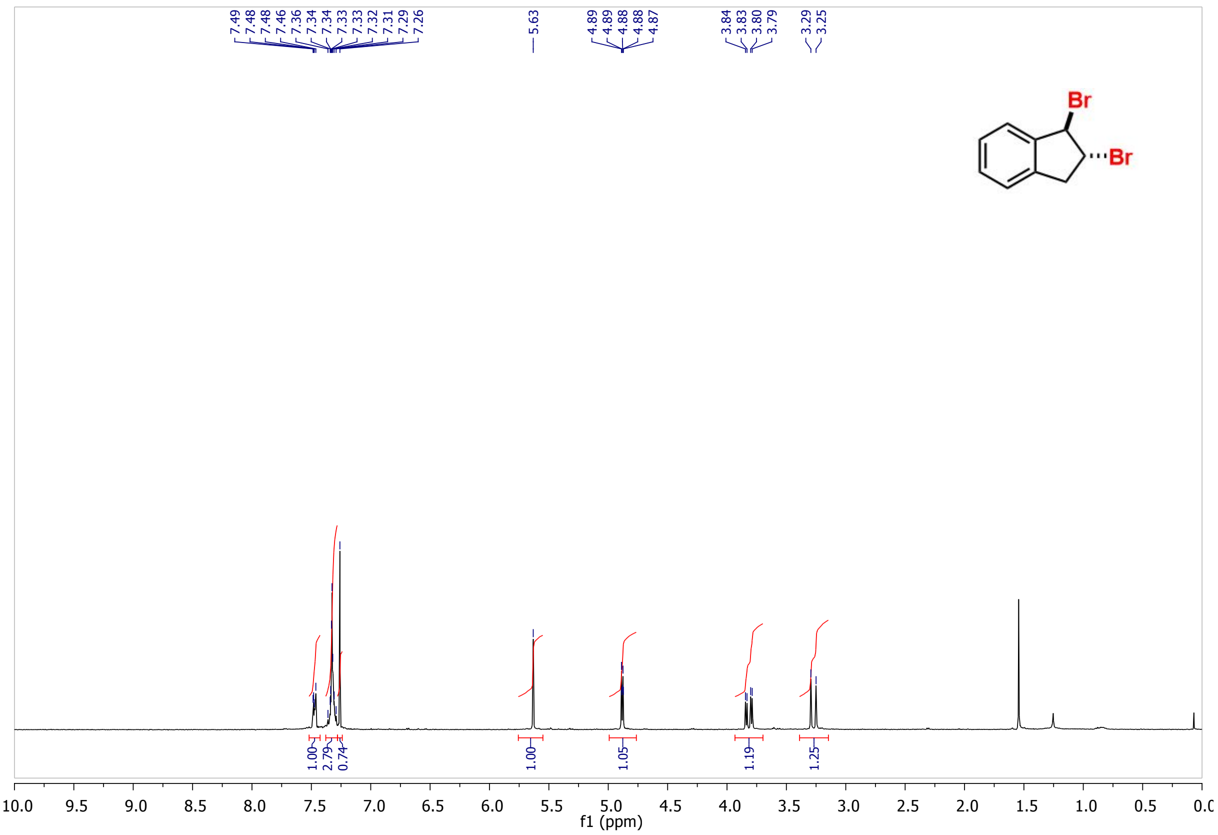

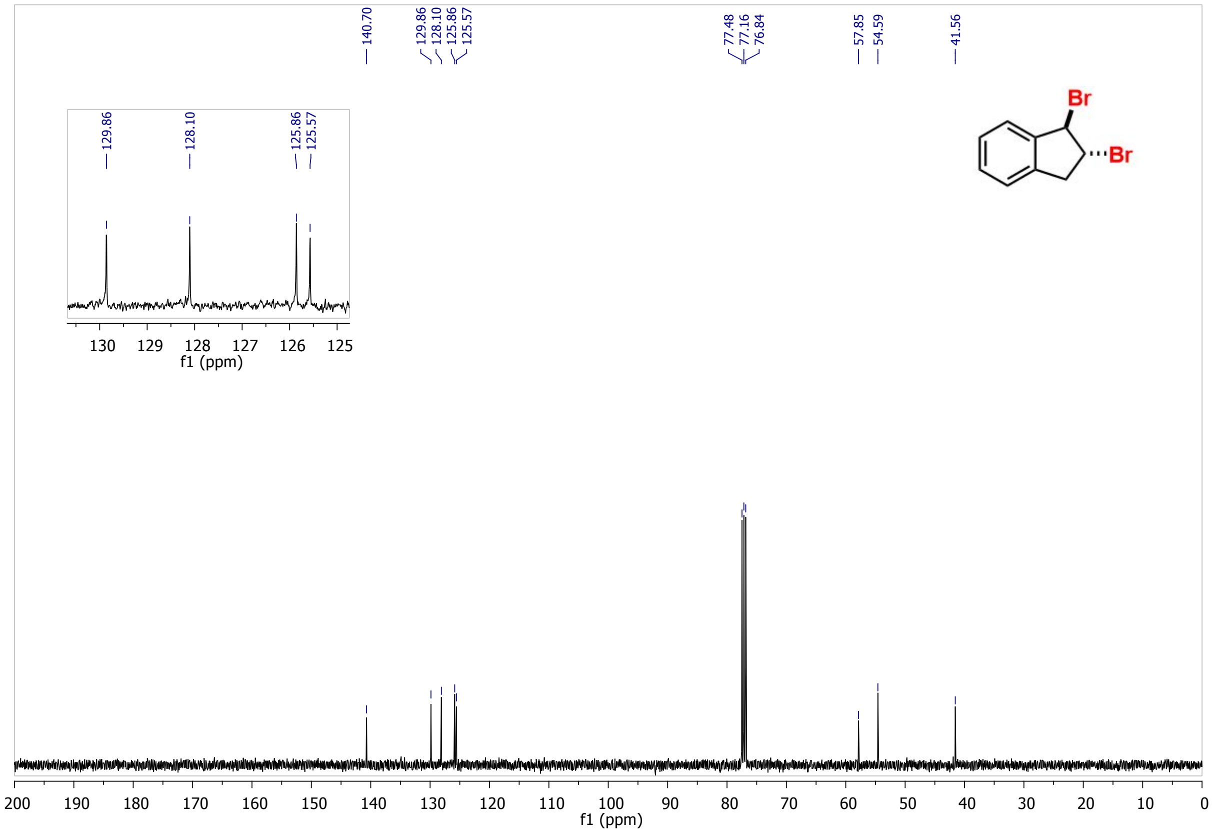

400 MHz ^{1}H-NMR (top) and 101 MHz ^{13}C-NMR (bottom) spectra of **3g** ($CDCl_3$)

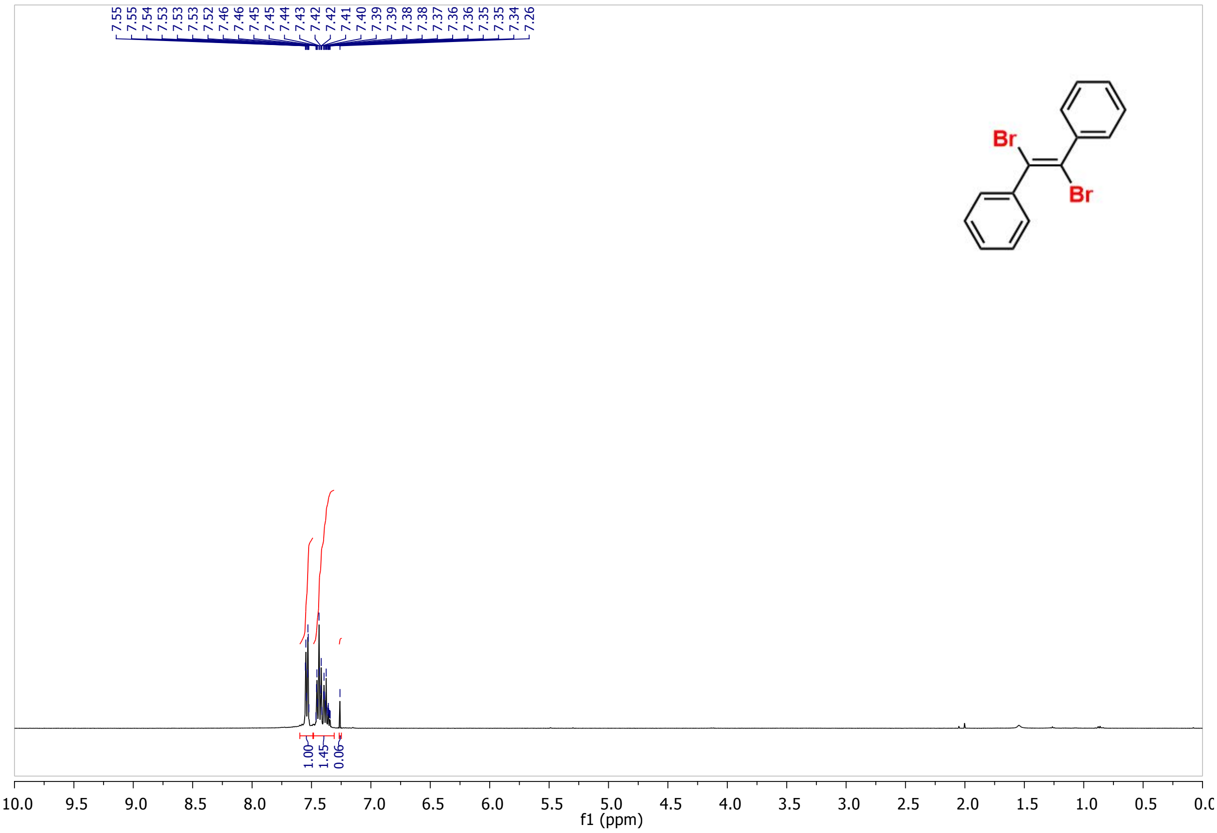

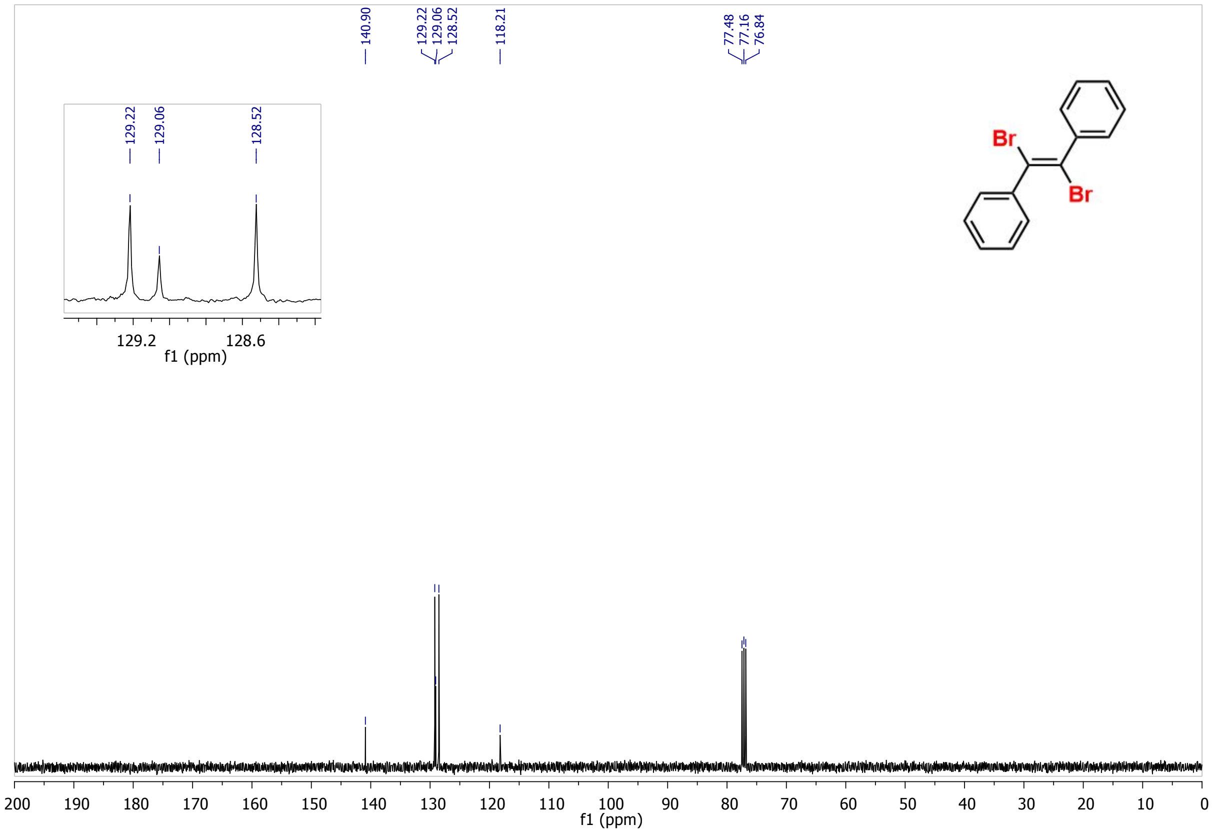

400 MHz ^{1}H-NMR (top) and 101 MHz ^{13}C-NMR (bottom) spectra of **3h** ($CDCl_3$)

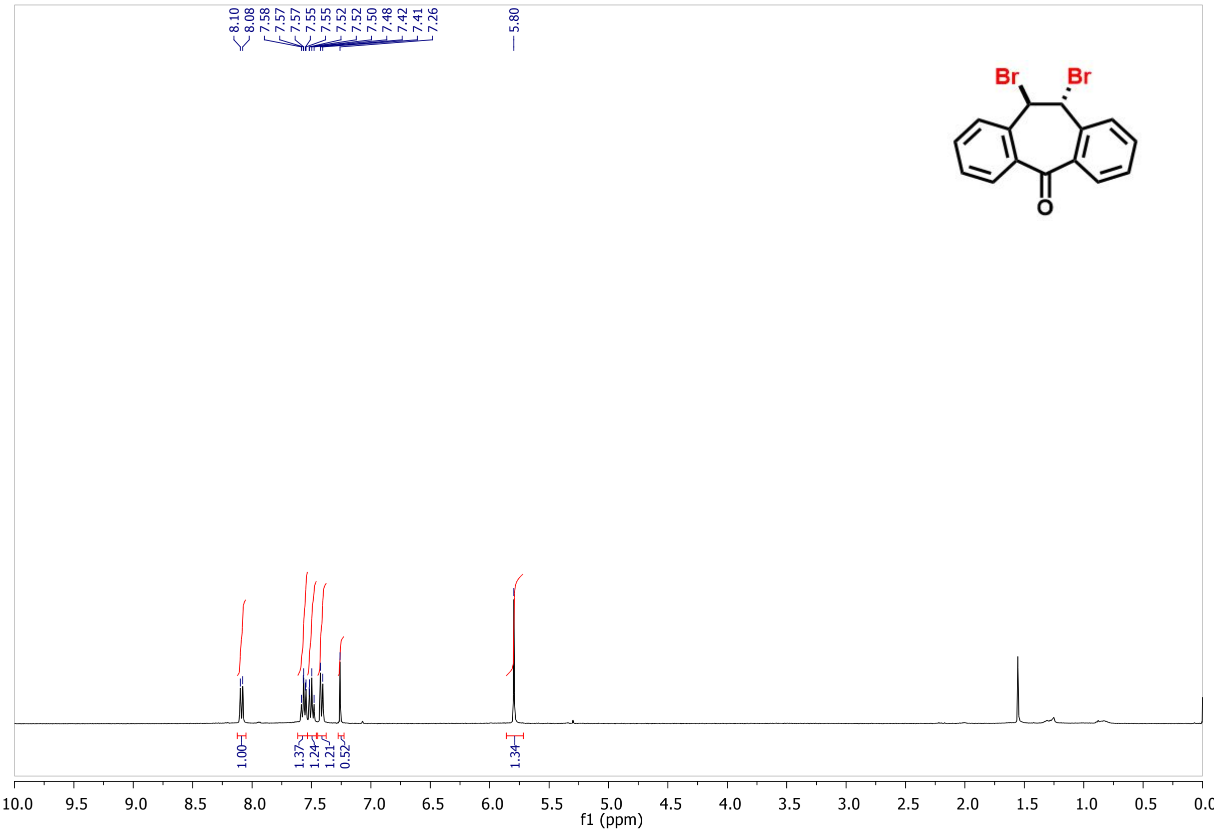

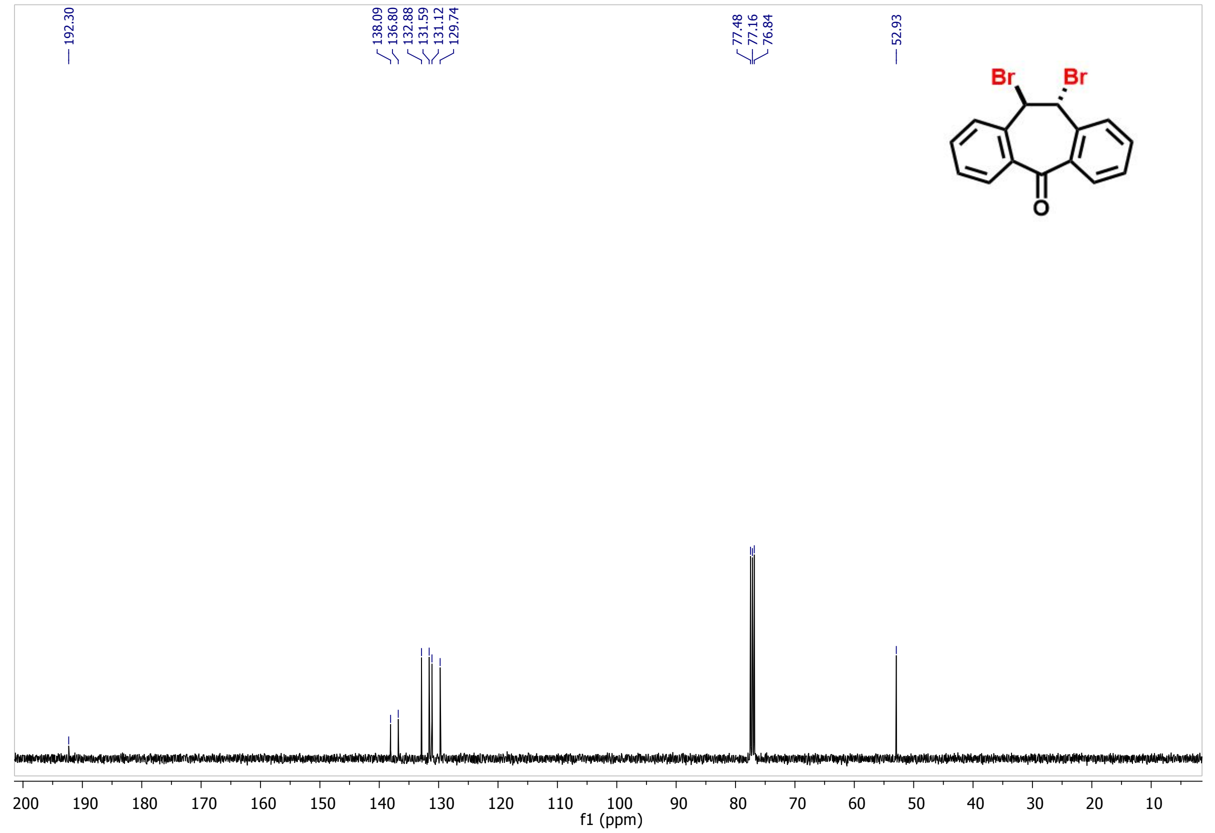

400 MHz ^{1}H-NMR (top) and 101 MHz ^{13}C-NMR (bottom) spectra of **3i** ($CDCl_3$)

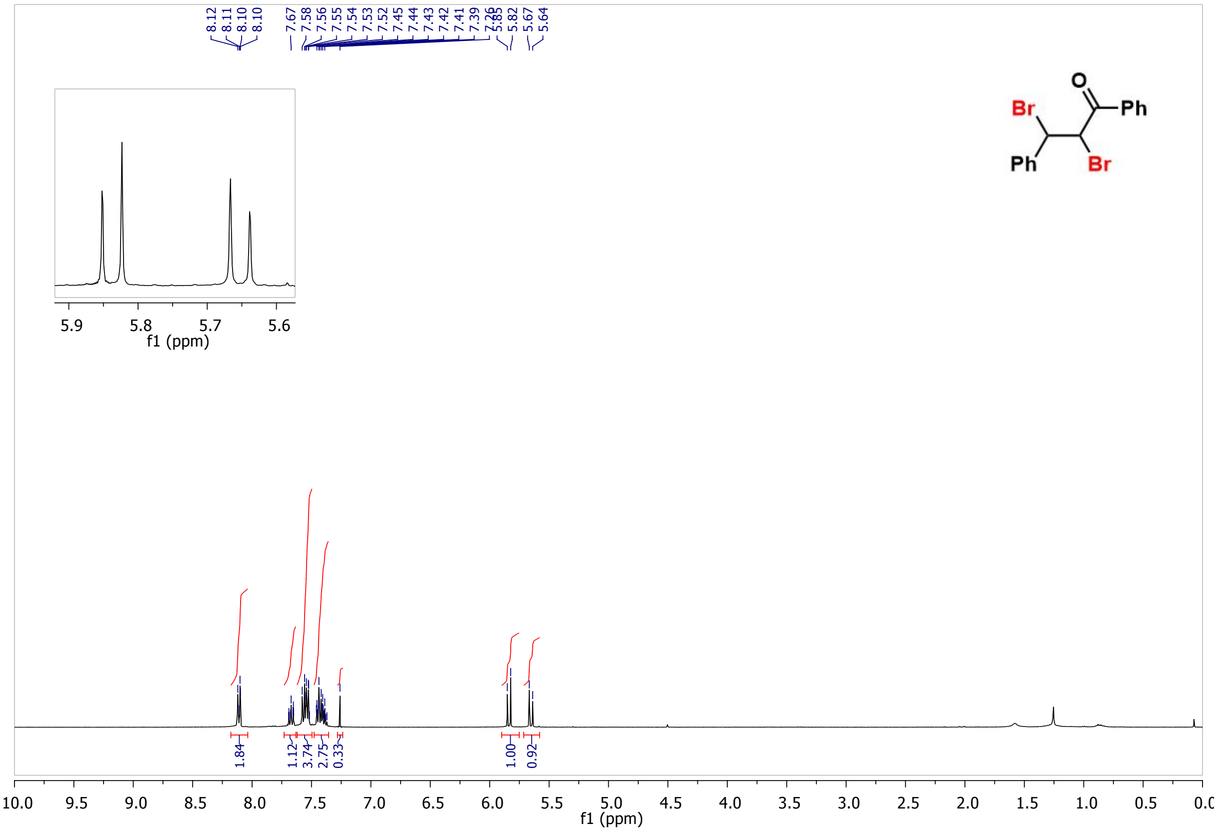

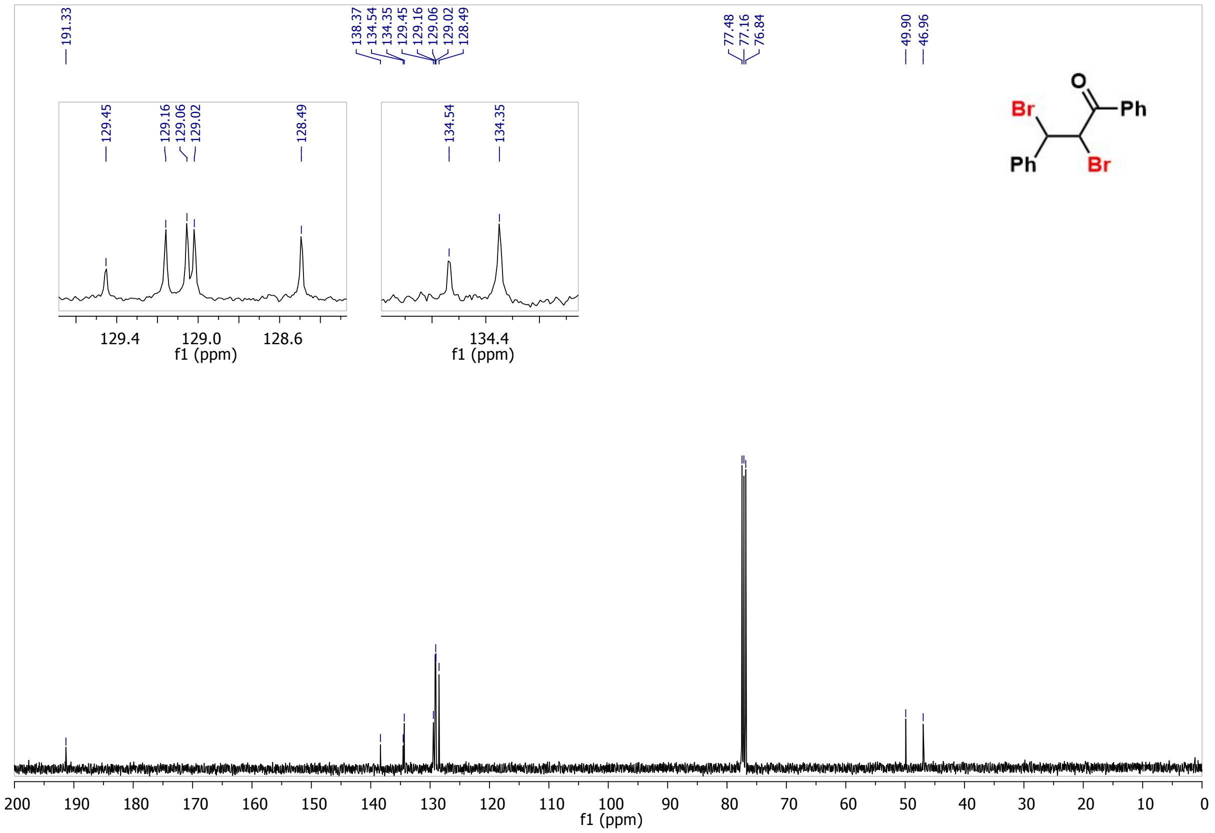

400 MHz ^{1}H-NMR (top) and 101 MHz ^{13}C-NMR (bottom) spectra of **3j** ($CDCl_3$)

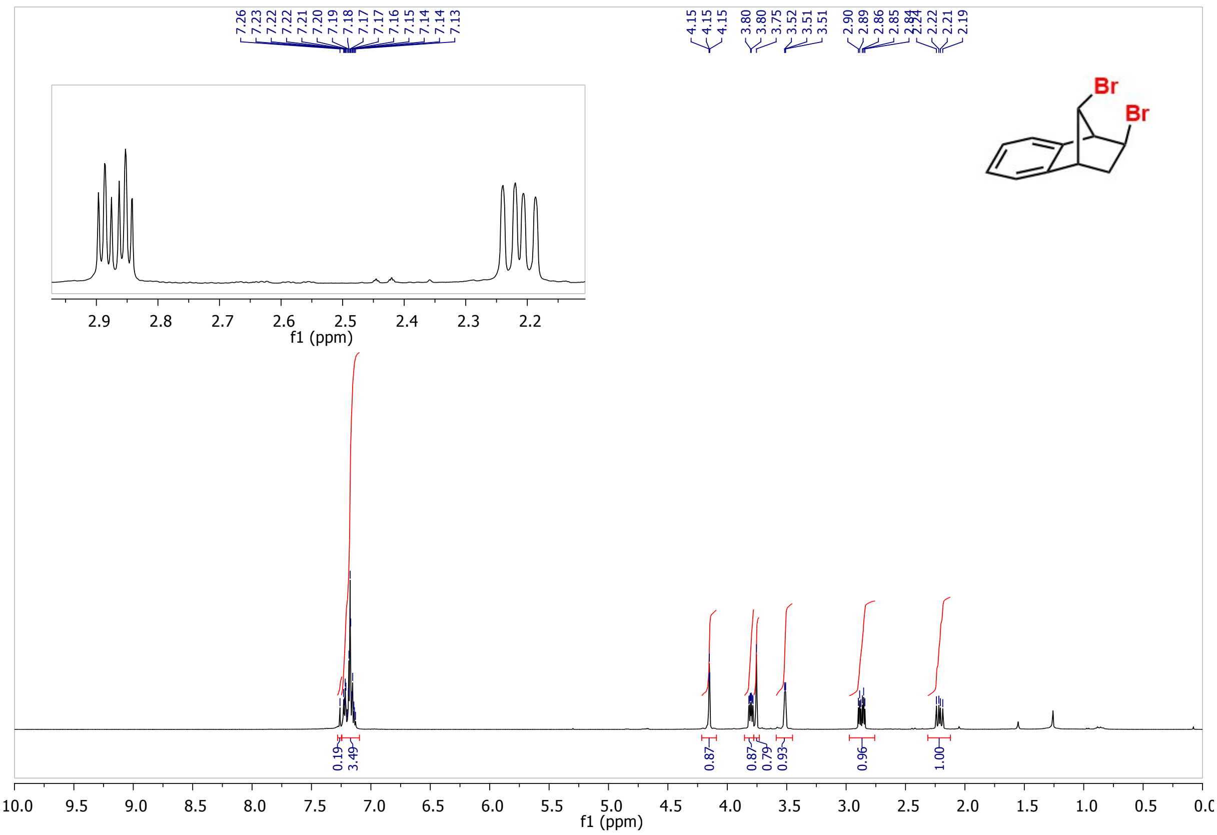

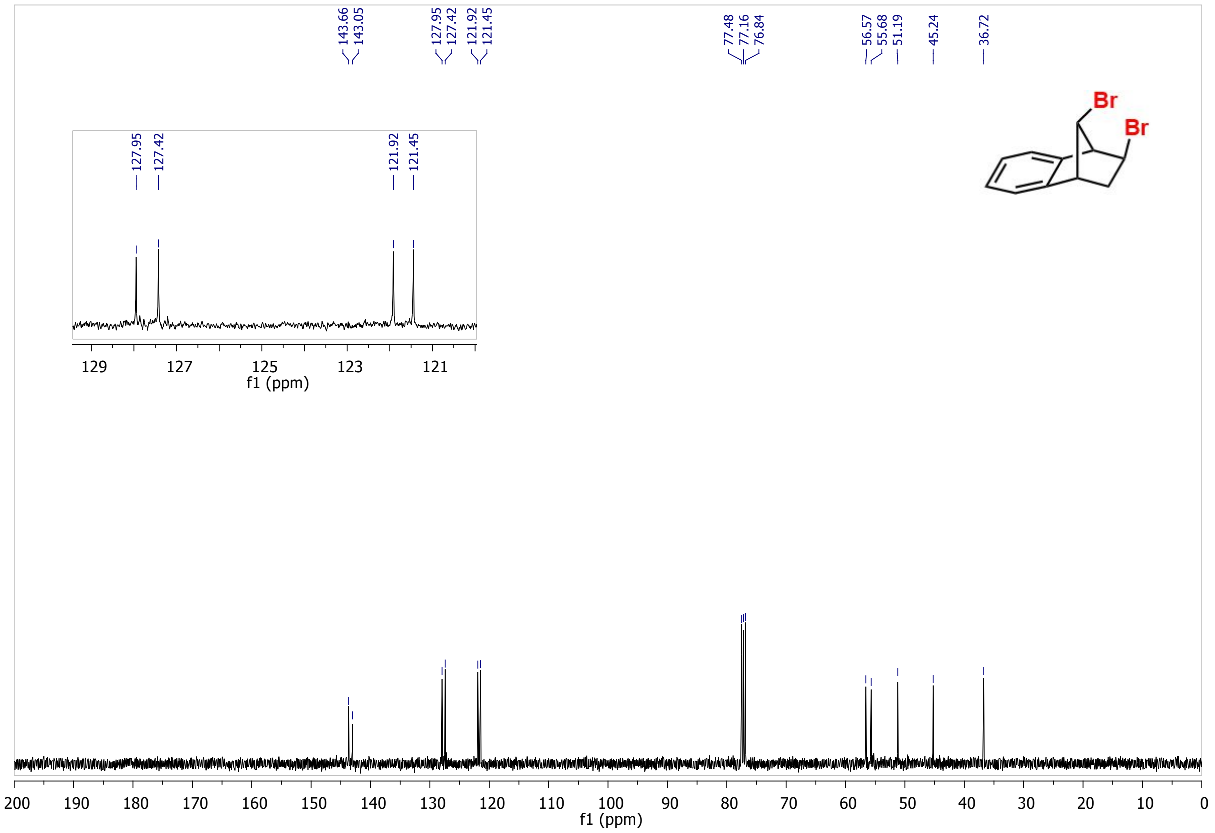

400 MHz ^{1}H-NMR (top) and 101 MHz ^{13}C-NMR (bottom) spectra of **3k** ($CDCl_3$)

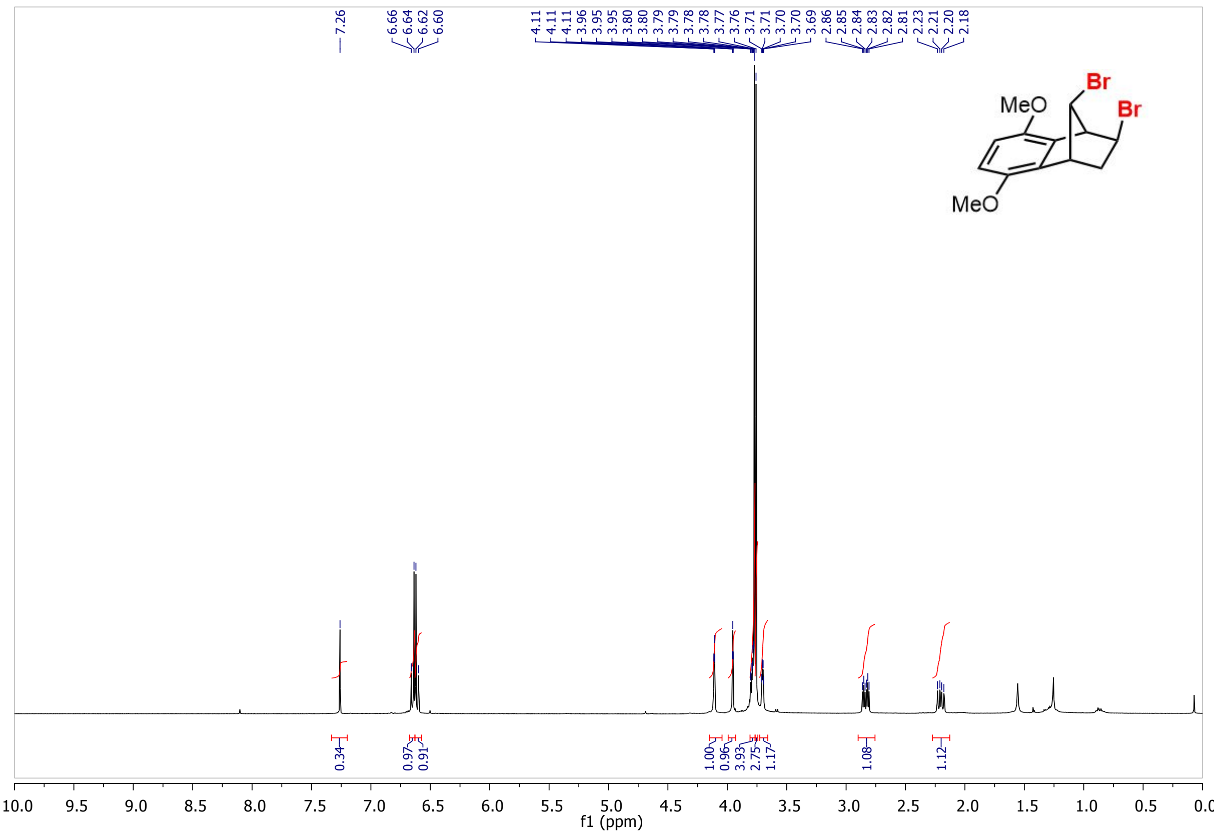

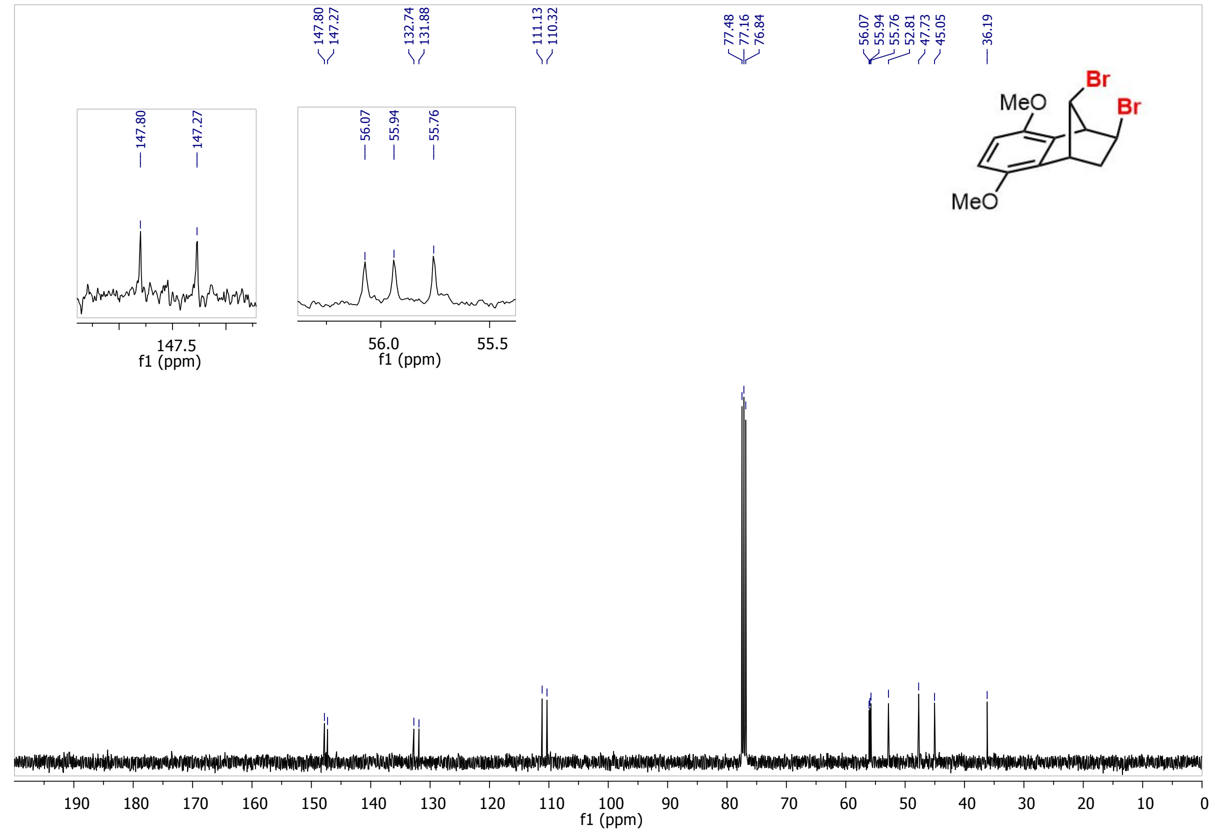

400 MHz ^{1}H-NMR (top) and 101 MHz ^{13}C-NMR (bottom) spectra of **3l** ($CDCl_3$)

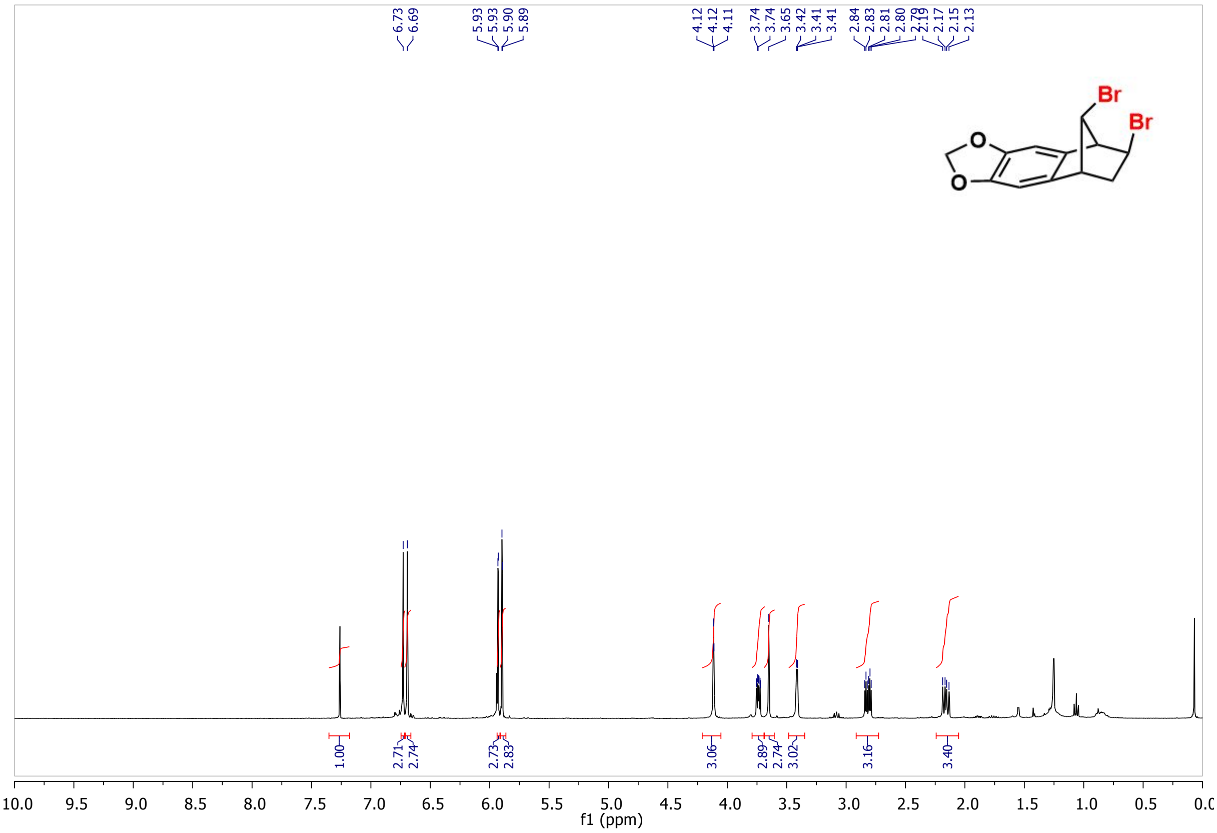

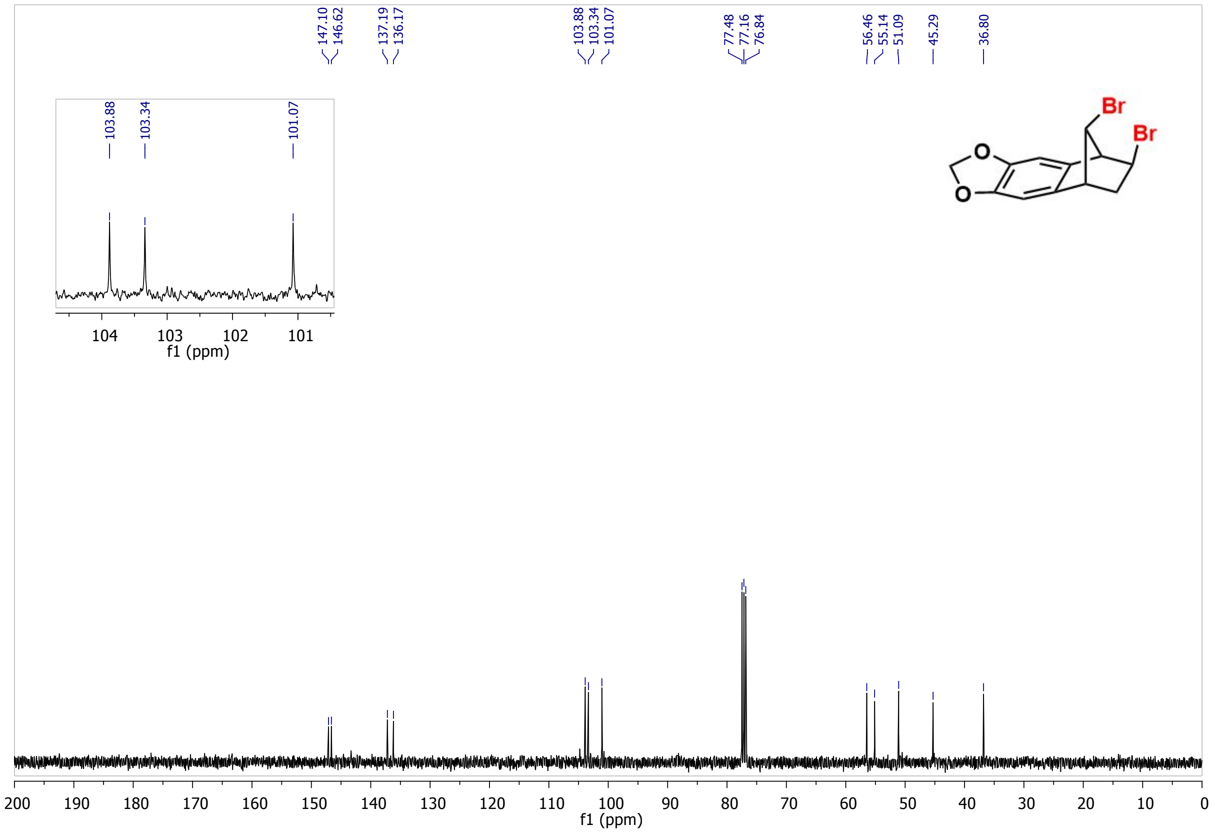

400 MHz ^{1}H-NMR (top) and 101 MHz ^{13}C-NMR (bottom) spectra of **3m** ($CDCl_3$)

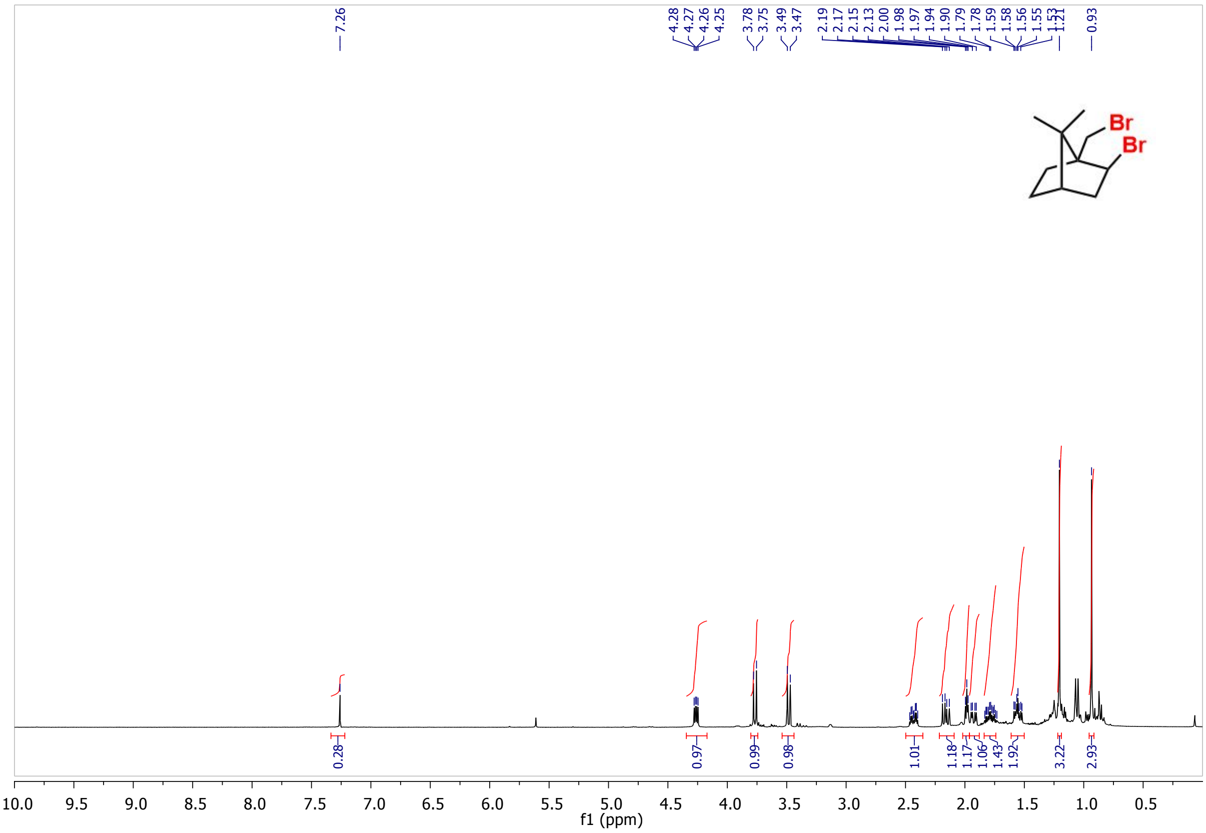

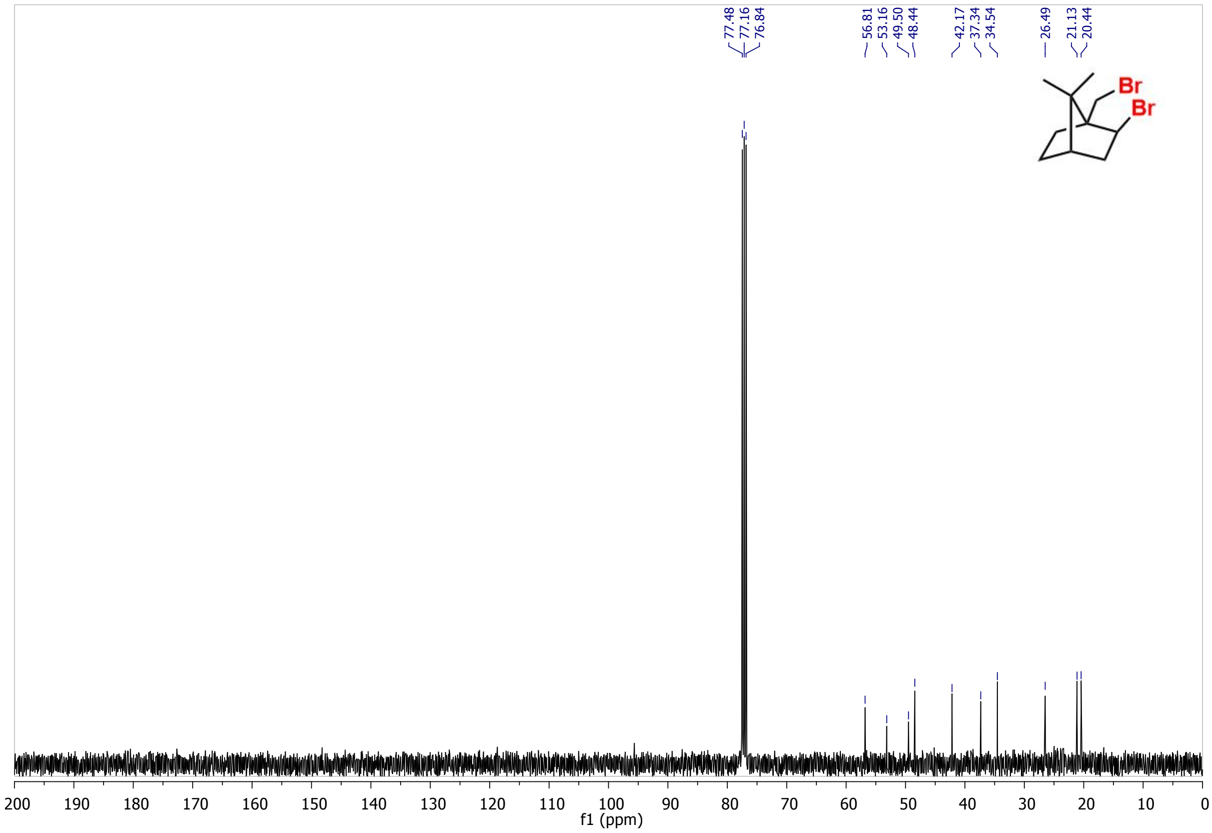

400 MHz ^{1}H-NMR (top) and 101 MHz ^{13}C-NMR (bottom) spectra of **3n** ($CDCl_3$)

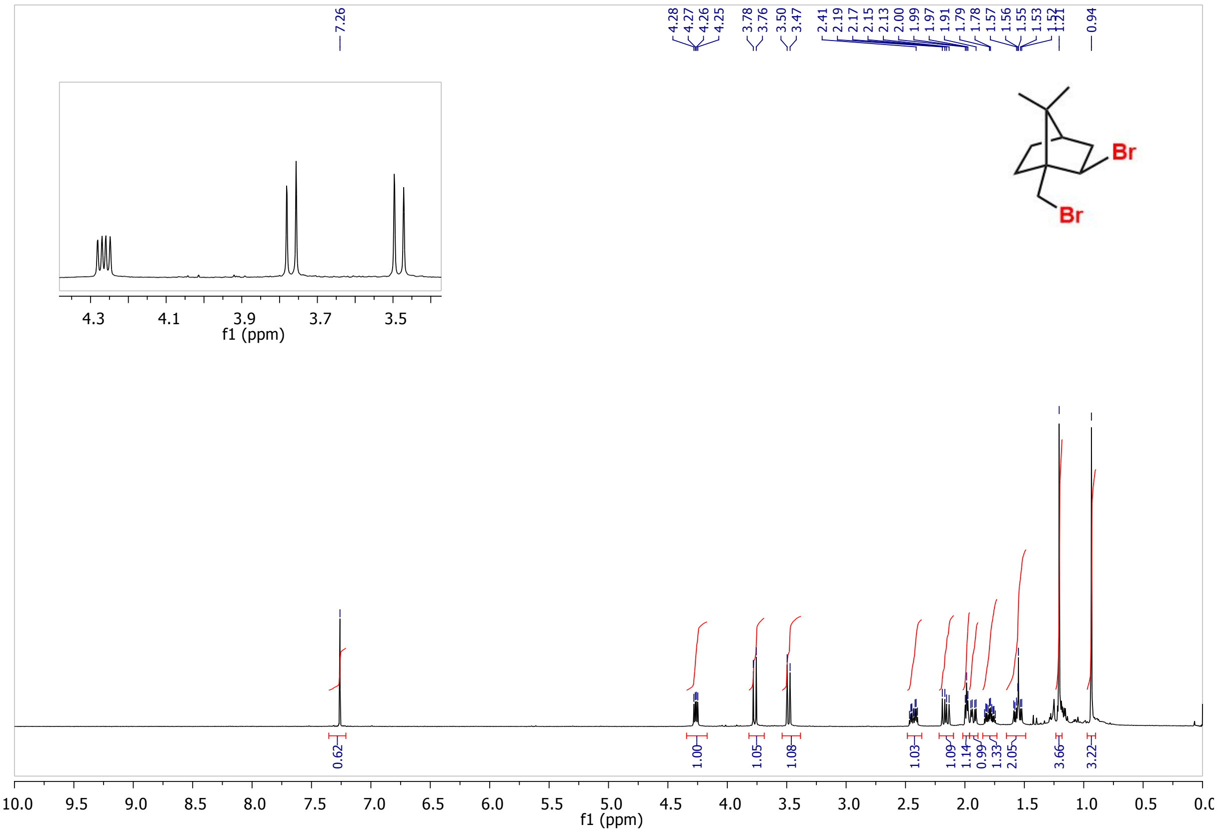

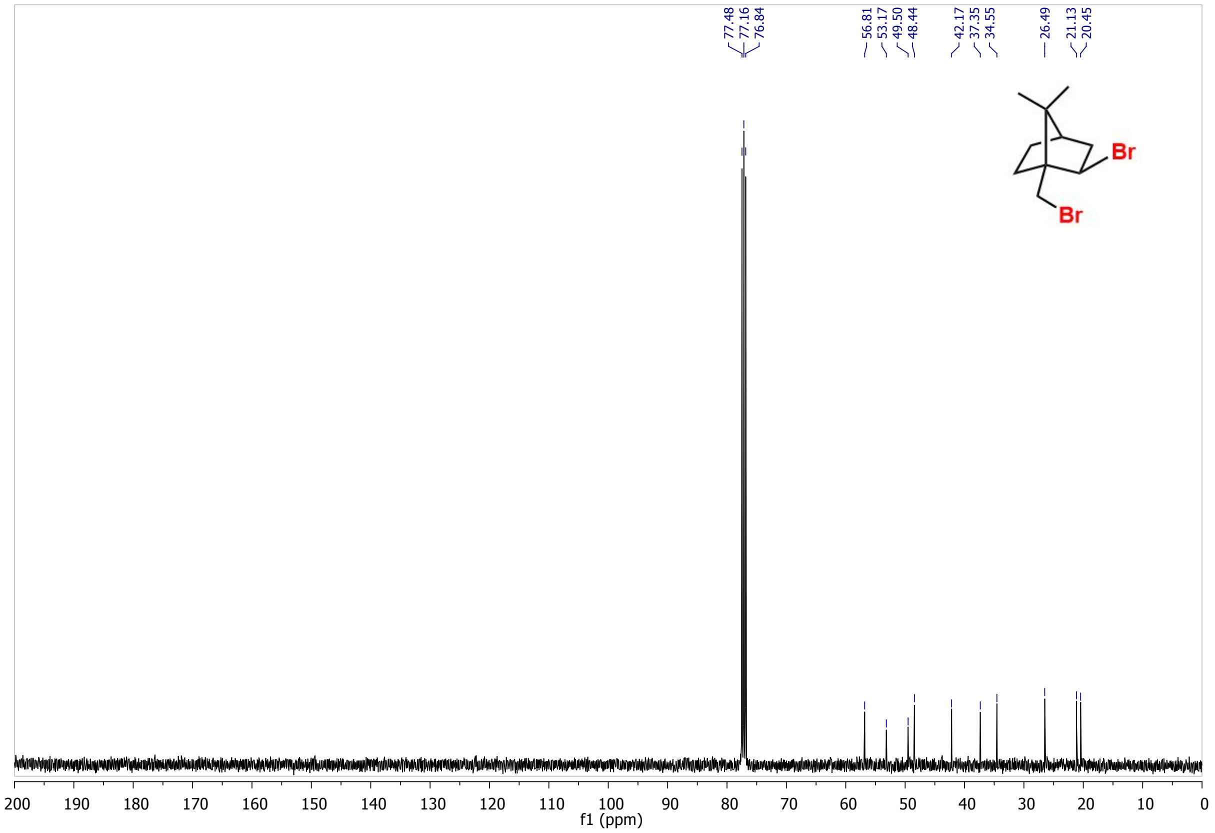

400 MHz ^{1}H-NMR (top) and 101 MHz ^{13}C-NMR (bottom) spectra of **3o** ($CDCl_3$)

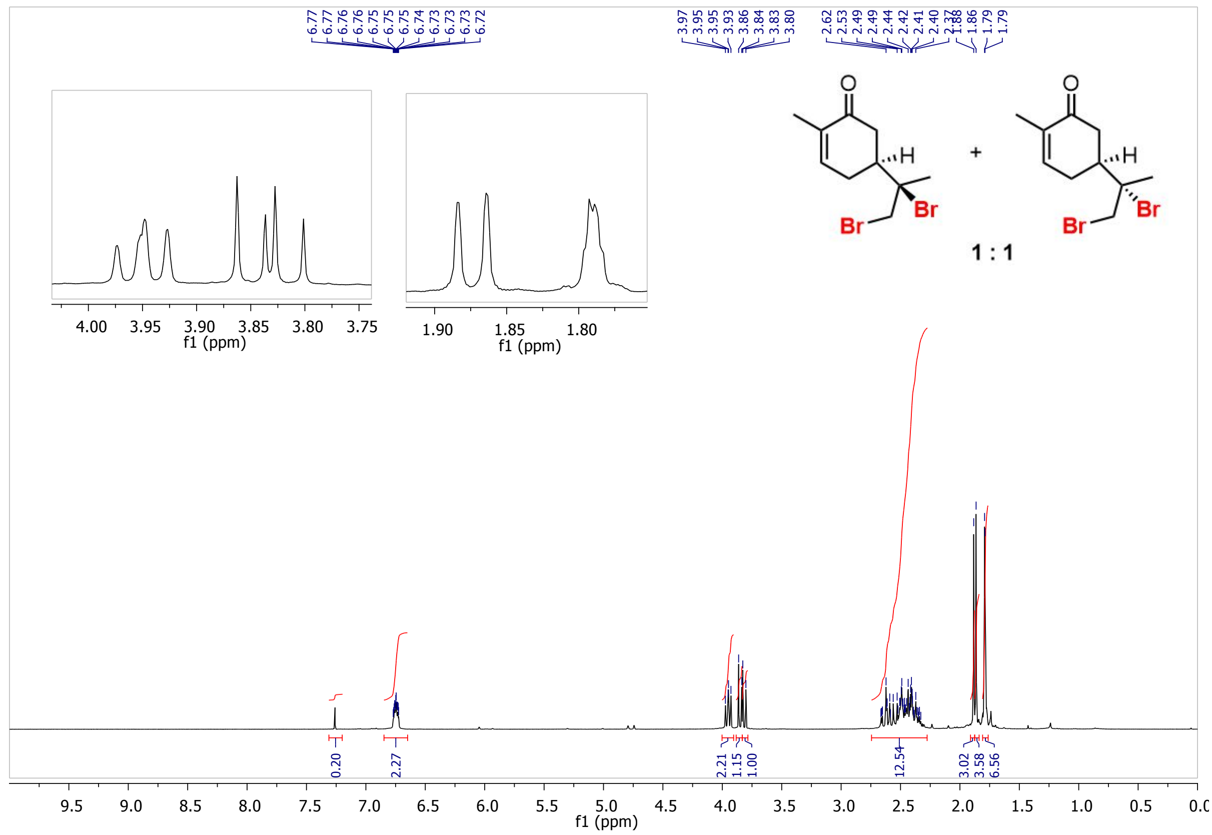

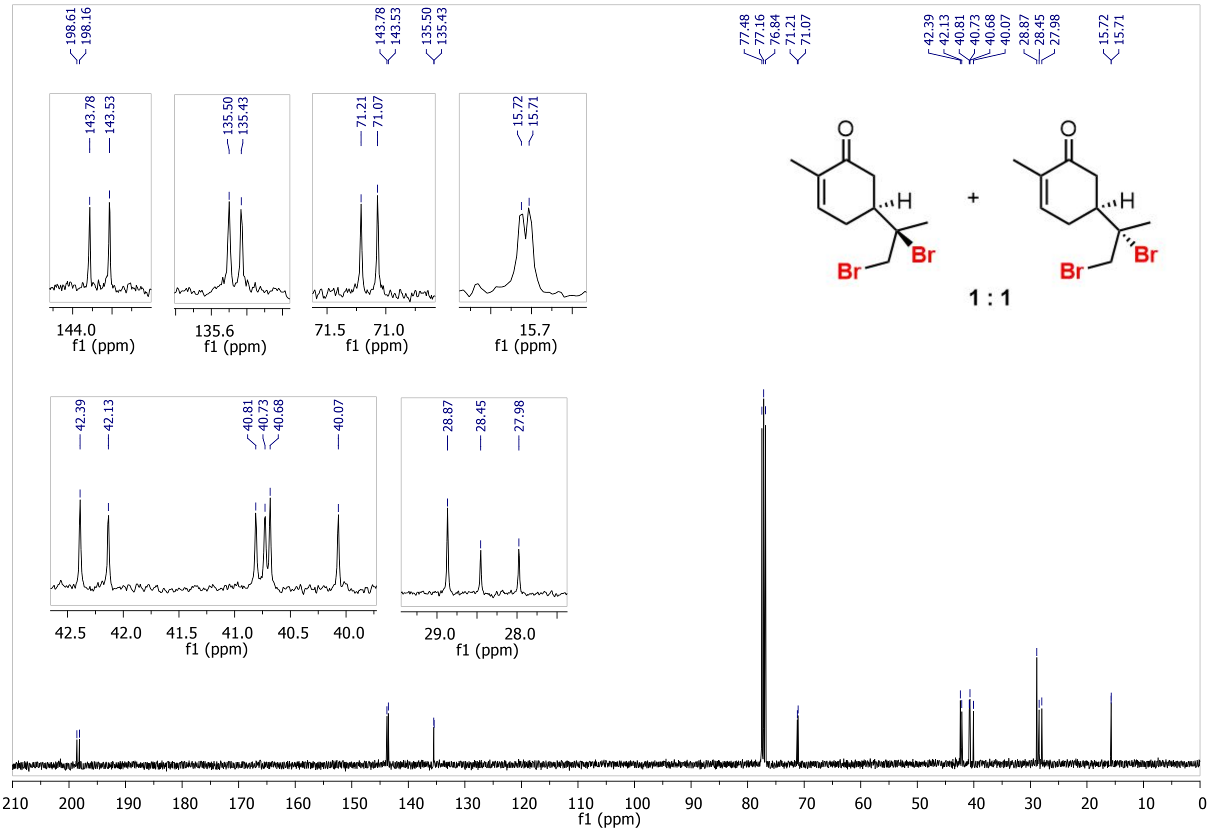

400 MHz ^{1}H-NMR (top) and 101 MHz ^{13}C-NMR (bottom) spectra of mixture of **3p** and **3q** ($CDCl_3$)

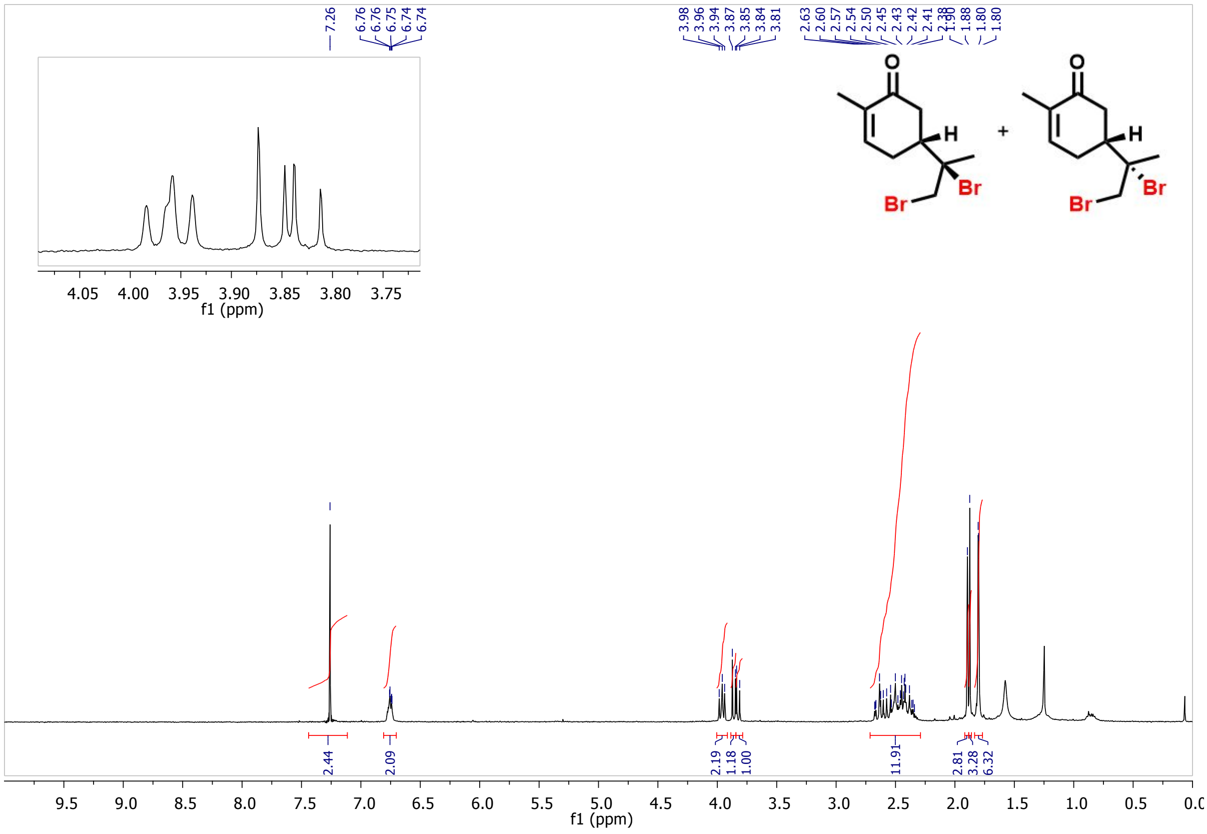

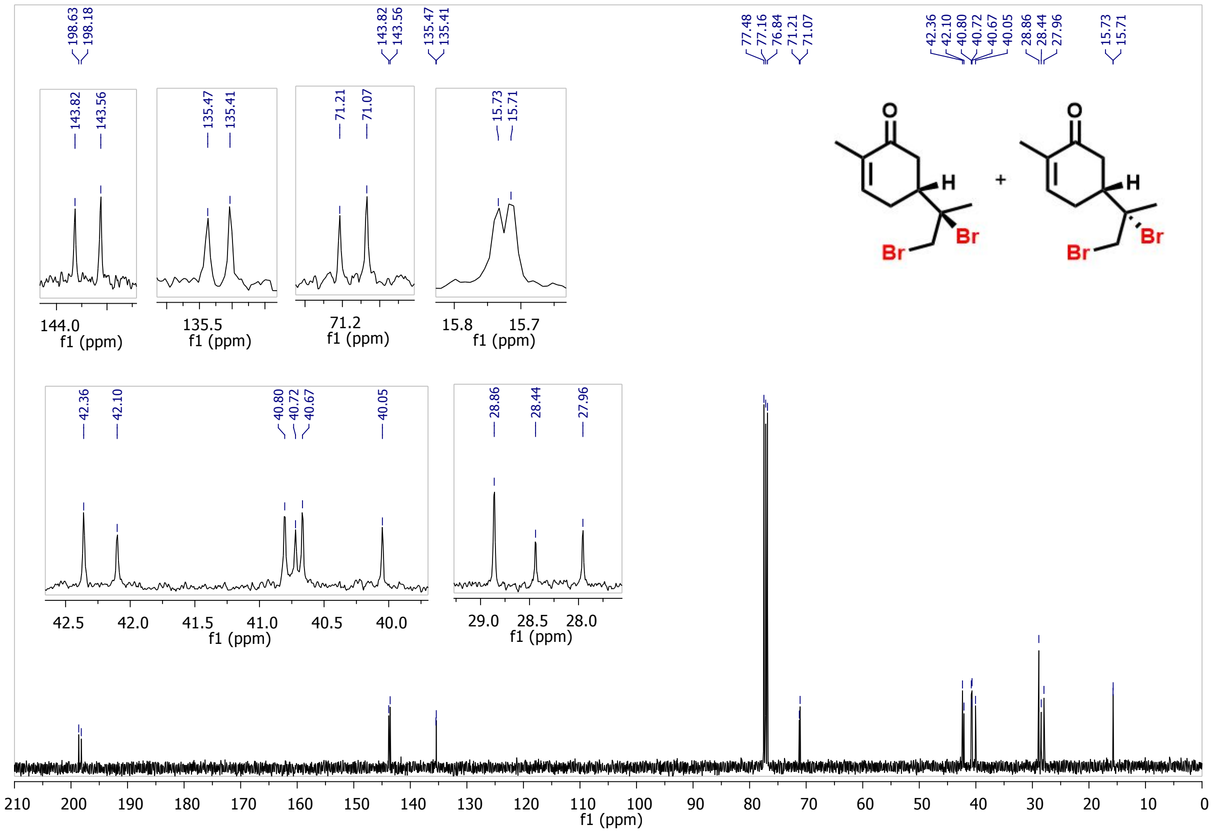

400 MHz ^{1}H-NMR (top) and 101 MHz ^{13}C-NMR (bottom) spectra of mixture of **3r** and **3s** ($CDCl_3$)

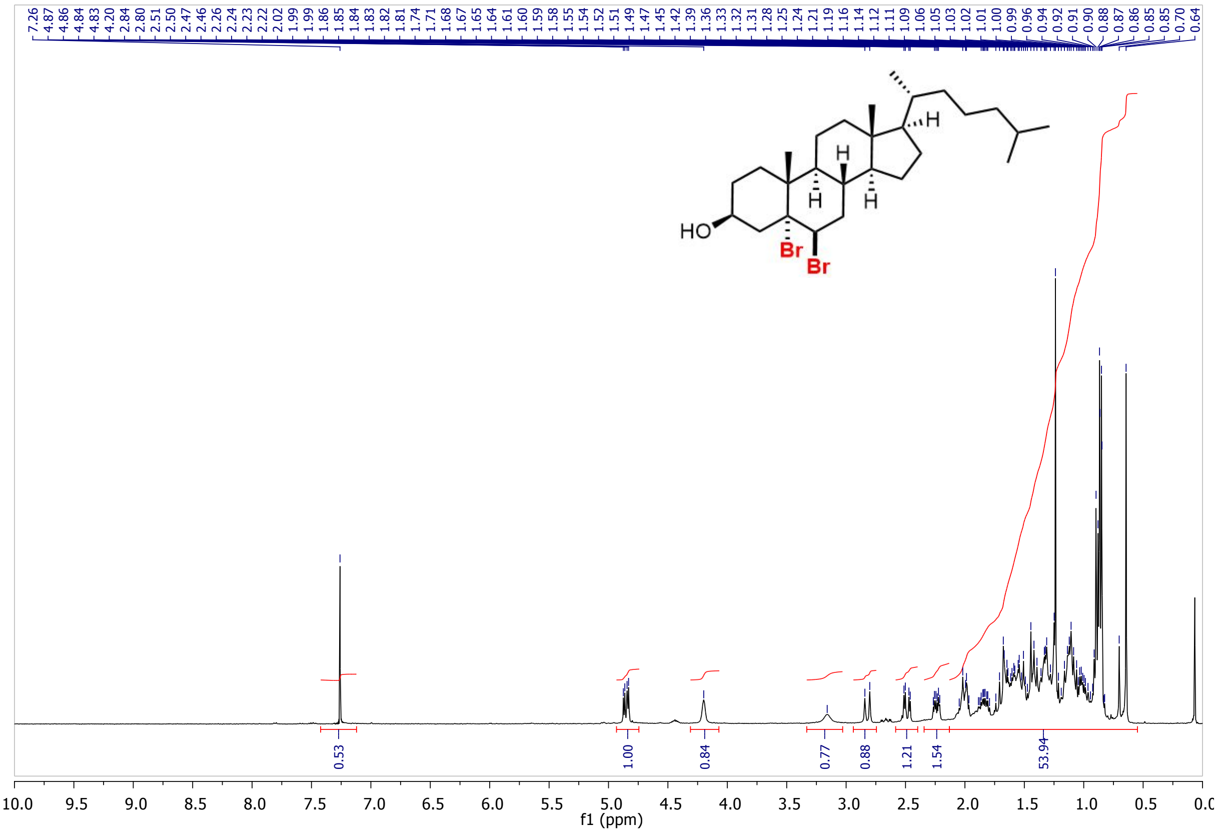

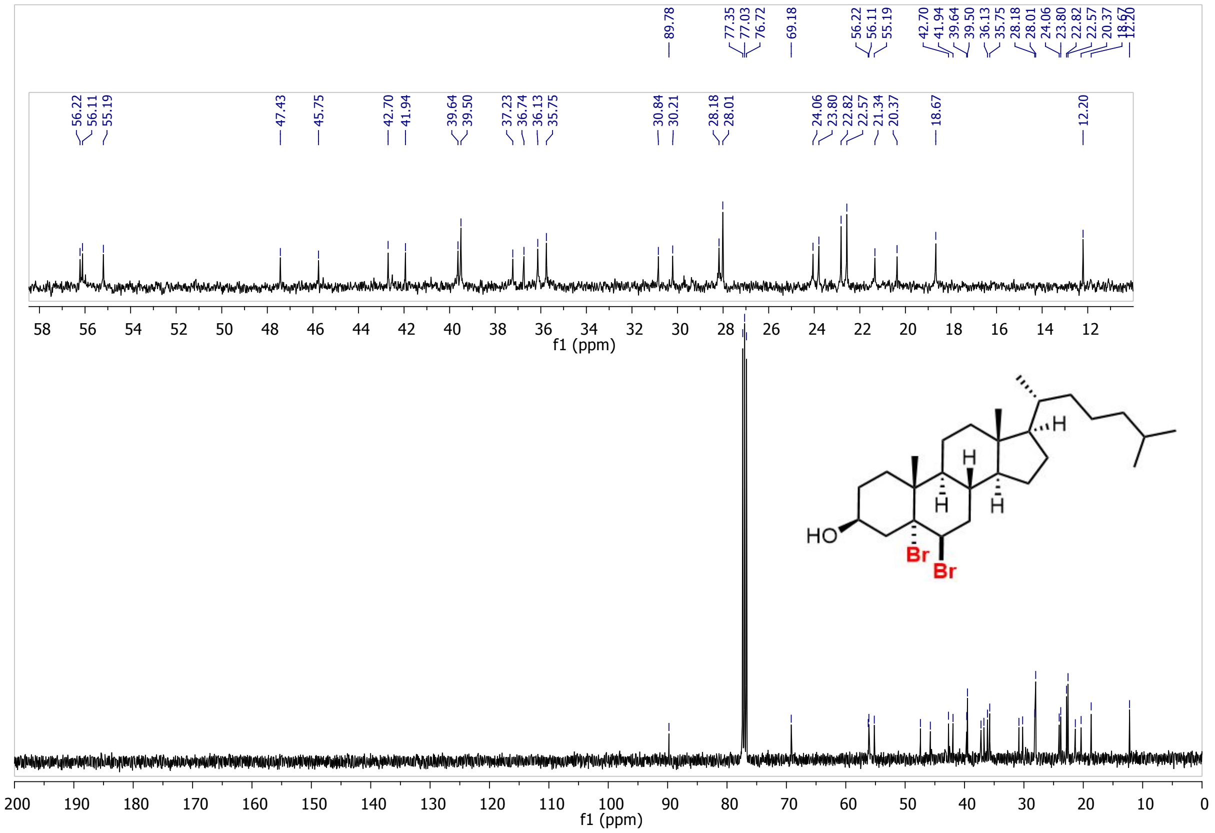

400 MHz ^{1}H-NMR (top) and 101 MHz ^{13}C-NMR (bottom) spectra of **3t** ($CDCl_3$)

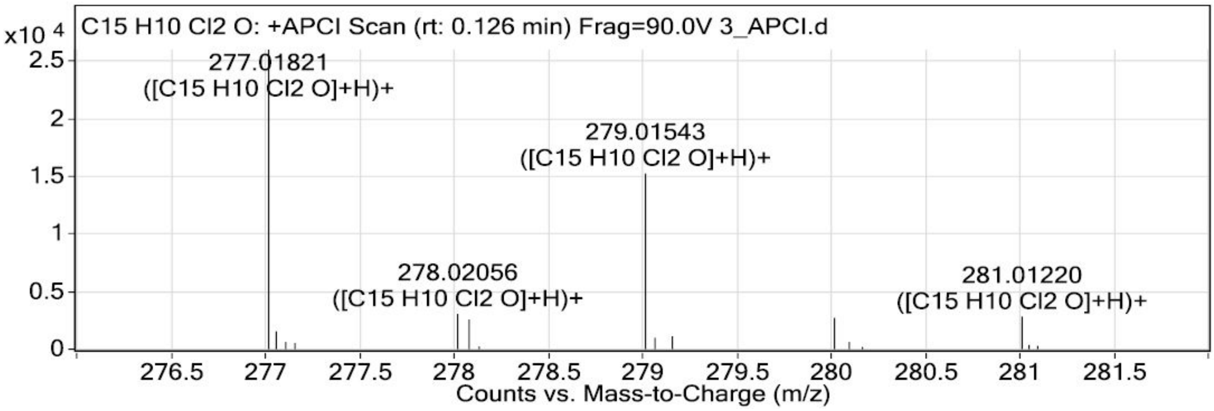

HRMS spectrum of **2i**.

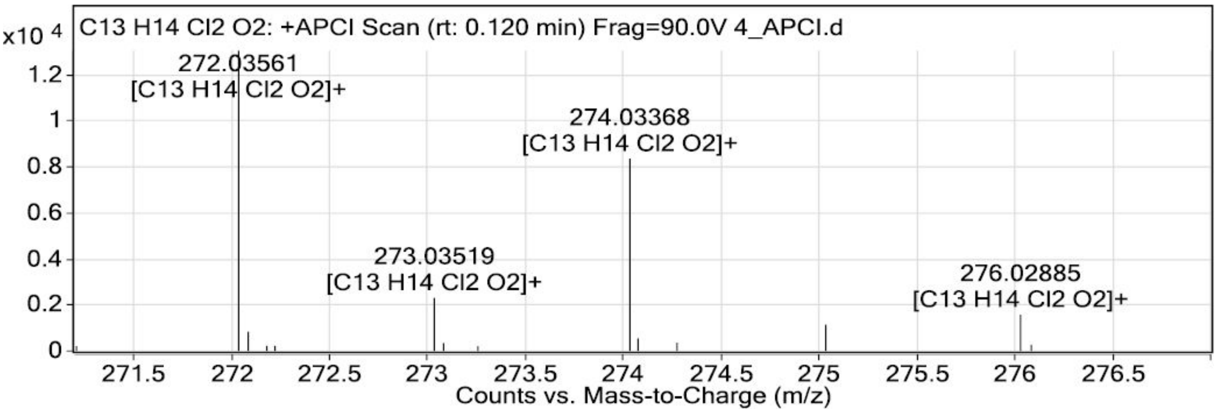

HRMS spectrum of **2l**.

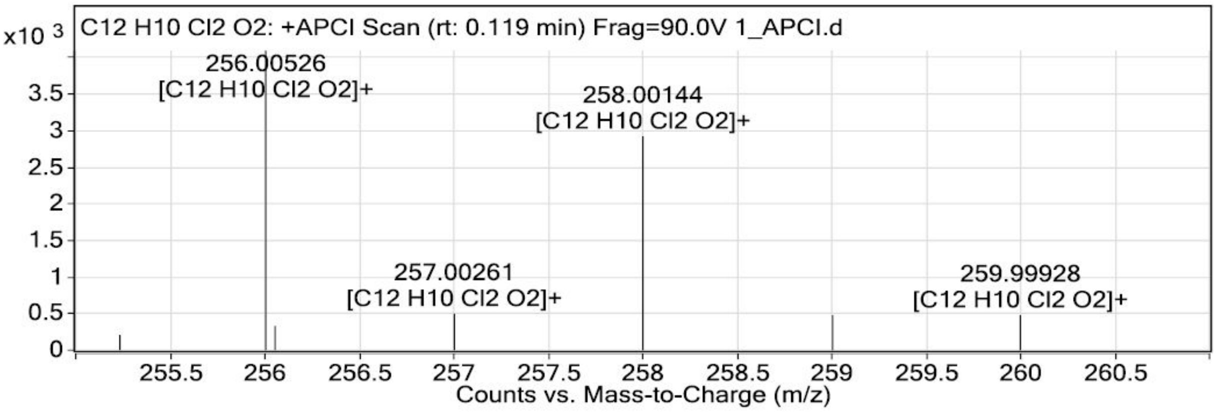

HRMS spectrum of **2m**.

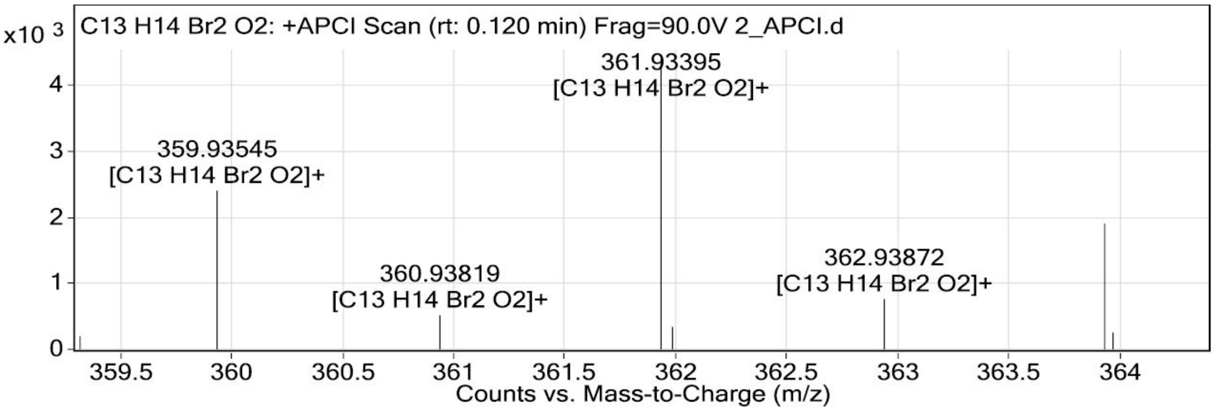

HRMS spectrum of **3l**.

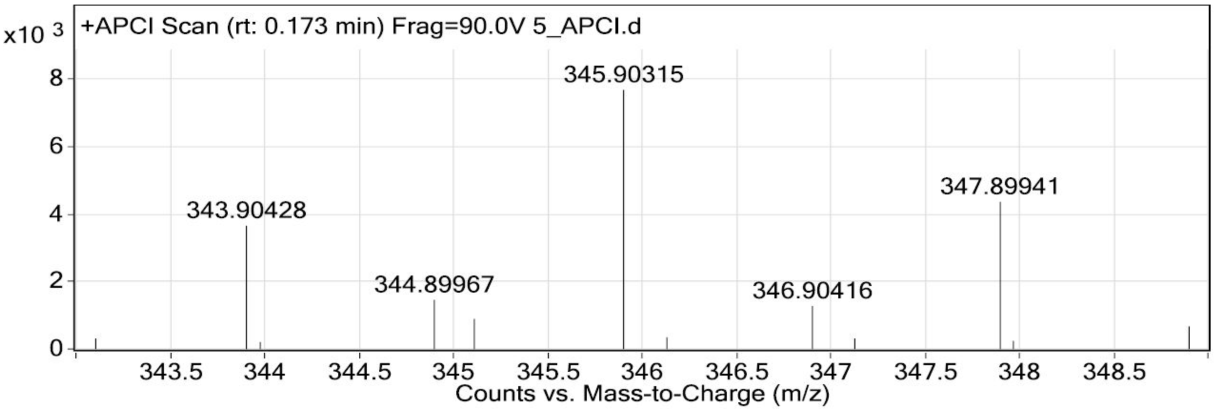

HRMS spectrum of **3m**.
